# Nutritional supplements improve cardiovascular risk factors in overweight and obese patients: A Bayesian network meta-analysis

**DOI:** 10.3389/fnut.2023.1140019

**Published:** 2023-03-30

**Authors:** Zengli Yu, Danyang Zhao, Xinxin Liu

**Affiliations:** Department of Nutrition and Hygiene, College of Public Health, Zhengzhou University, Zhengzhou, Henan, China

**Keywords:** obesity, nutritional supplement, cardiovascular risk factors, body composition, probiotics, Bayesian network meta-analysis

## Abstract

**Background:**

Overweight and obesity are considered as one of the major risk factors for cardiovascular diseases (CVD). At present, many studies have proved that multiple nutritional supplements play an active role in metabolic diseases. However, the comparative efficacy of different nutritional supplements in improving indicators of cardiometabolic risk in obese and overweight patients is uncertain.

**Methods:**

Cochrane Library, PubMed, Embase, and Web of Science were searched for the period from January 1990 to March 2022. A random-effect model was built in the Bayesian network meta-analysis. The surface under the cumulative ranking analysis (SUCRA) and clustering rank analysis was performed for ranking the effects.

**Results:**

The study included 65 RCTs with 4,241 patients. In terms of glucose control, probiotic was more conductive to improve FBG (MD: −0.90; 95%CrI: −1.41 to −0.38), FINS (MD: −2.05; 95%CrI: −4.27 to −0.02), HOMA-IR (MD: −2.59; 95%CI −3.42 to −1.76). Probiotic (MD: −11.15, 95%CrI −22.16 to −1.26), omega-3 (MD: −9.45; 95%CrI: −20.69 to −0.93), VD (MD: −17.86; 95%CrI: −35.53 to −0.27), and probiotic +omega-3 (MD: 5.24; 95%CrI: 0.78 to 9.63) were beneficial to the improvement of TGs, TC and HDL-C, respectively. The SUCRA revealed that probiotic might be the best intervention to reduce FBG, FINS, HOMA-IR; Simultaneously, α-lipoic acid, VD, and probiotic + omega-3 might be the best intervention to improve TGs, TC, and HDL-C, respectively. Cluster-rank results revealed probiotic had the best comprehensive improvement effect on glucose metabolism, and probiotic + omega-3 may have a better comprehensive improvement effect on lipid metabolism (cluster-rank value for FBG and FINS: 3290.50 and for TGs and HDL-C: 2117.61).

**Conclusion:**

Nutritional supplementation is effective on CVD risk factors in overweight and obese patients. Probiotic supplementation might be the best intervention for blood glucose control; VD, probiotic + omega-3 have a better impact on improving lipid metabolism. Further studies are required to verify the current findings.

## 1. Introduction

The World Health Organization (WHO) defines overweight and obesity as abnormal or excessive fat accumulation that may damage health (1). Obesity is a threat to global population health in terms of prevalence and disease burden. In recent research, 2 billion people was diagnosed with overweight or obesity (2). In obese and overweight patients, the active metabolism of adipose tissue induces metabolic changes, such as increased production of reactive oxygen species, oxidative stress and inflammation, leading to type 2 diabetes mellitus (T2DM), arterial hypertension and dyslipidemia, which are the most important precursor risk factors for cardiovascular diseases (CVD) (3). Cardiometabolic biomarkers, such as blood glucose, insulin resistance and lipid profiles, are important risk indicators of subclinical disease and a valuable tool for monitoring CVD (4–6). Therefore, improving the metabolic status of overweight and obese patients is an important preventive strategy to prevent the development of more serious metabolic diseases.

Since most of the drugs used to treat obesity have been withdrawn from the market due to improper use or side effects, lifestyle change and diet control are the safest and most cost-effective interventions for obese and overweight people to control their weight (7–9). Some nutrients not only have antioxidant, anti-inflammatory and immune-enhancing biological activities, but also have greater safety compared with drugs. Currently available nutritional supplements such as vitamins, minerals, fatty acids, and plant compounds have been shown to improve obesity by improving carbohydrate metabolism, increasing lipolysis or energy expenditure, and reducing hunger (10). Therefore, they have attracted extensive attention in the treatment of metabolic diseases. According to previous meta-analysis, resveratrol, Vitamin D (VD)/VD + calcium (Ca), probiotics, α-lipoic acid, omega-3, curcumin, and magnesium (Mg) were used to improve multiple comorbidities of metabolic disorders (11–17). While most RCTs and meta-analysis to date have proved the beneficial effect of nutritional supplements on metabolism diseases patients, limited data are available regarding their effects on other indicators of CVD risk, i.e., metabolic syndrome (MetS) (18), elevated blood pressure (19), endothelial function (20), and in other at-risk populations. Obesity, particularly intra-abdominal obesity, predisposes people to several modifiable risk factors of CVD and T2DM, i.e., cardiometabolic risk (21). Furthermore, it is difficult to determine the comprehensive efficiency of different nutritional strategies using pair-wise meta-analysis.

The effect of different nutritional supplements for overweight and obesity patients on cardiovascular risk factors, as well as which intervention is most effective, remain to be verified. Therefore, in this study, we aimed to conduct systematic review and network meta-analysis (NMA) by comparing the adjuvant therapy of different nutritional supplements for overweight and obese adults, so as to provide reference for clinical practice.

## 2. Methods

This systematic review was prepared according to the preferred Reporting Items for Systematic Review and Meta-Analysis (PRISMA) (22) as well as the PRISMA extension statement for network meta-analysis (23) (Supplementary File 1) and was registered at the international Prospective Register of Systematic Reviews (CRD42022371086).

### 2.1. Search strategy

Two independent researchers searched PubMed, EMBASE, Web of Science and Cochrane Library from the inception of each database to March 20, 2022, and the search strategy was based on the standards established by the Cochrane Collaboration. The search was limited to human subjects’ studies and English language publications. We use both medical subject heading (MeSH) and extensive free-text keywords, and search terms included: random*, adults, obesity, overweight, supplementation, nutrition, resveratrol, Vitamin D, probiotics, α-lipoic acid, omega-3, curcumin, magnesium. The search strategy is shown in Supplementary File 2.

### 2.2. Eligibility criteria

In this network meta-analysis, randomized controlled trials (RCTs) which fulfilled the following criteria for participants, interventions, comparisons, outcomes, and study design (PICOS) were included: (1) Participants: We included studies of overweight or obese adults and excluded studies of other cardiovascular diseases (i.e., type 2 diabetes, insulin resistance, non-alcoholic fatty liver disease, hyperlipemia, hypertension), children, adolescents or pregnant women. Overweight and obesity are defined as body mass index (BMI) ≥ 25 and 30 kg/m^2^, respectively. (2) Intervention: The intervention group used at least one of the following seven nutrition supplements: resveratrol, VD, probiotics, α-lipoic acid, omega-3, curcumin, Mg. The duration was at least 4 weeks. (3) Comparisons: Control, including groups that received placebo or those who received any nutrition supplements on the basis of nutritional treatment or maintaining the usual diet. (4) Outcomes: The parameters in the research results include at least two of the following parameters: cardiovascular risk factors [including systolic blood pressure (SBP), diastolic blood pressure (DBP), fasting blood glucose (FBG), fasting insulin level (FINS), homeostatic model assessment of insulin resistance (HOMA-IR), hemoglobin A1c (HbA1c), triglycerides (TGs), total cholesterol (TC), high-density lipoprotein cholesterol (HDL-C) and low-density lipoprotein cholesterol (LDL-C)] and body composition [including Weight, Waist circumference (WC), and BMI]. (5) Study design: Parallel or cross-over design.

### 2.3. Data extraction

Two researchers (D.Z. and Z.Y.) independently screened and assessed the titles and abstracts according to the prespecified criteria. The full texts of articles that potentially met the eligibility criteria were reviewed and data extracted using the same standardized data extraction methods. If more than one article from the same study was found, only the article with more detailed information was selected to avoid data duplication. The data was independently extracted and cross-checked by two researchers (D.Z. and Z.Y.), and any disagreement was resolved by the judgment of the third researcher (X.L.).

Information about study design was extracted, including study-level characteristics (i.e., first author name, year of publication, and geographic location), participant-level characteristics (i.e., age, proportion of male participants, and diet control or daily exercise), program-level characteristics (i.e., study design, sample size in each group, type and dose of nutritional supplementation, and outcome data). We extracted the preintervention/postintervention (pre/post) change data to conduct this NMA. Regarding the RCTs with multiple time points, only the last time point was considered and intermediary time points were omitted.

### 2.4. Quality assessment

Assessment of risk of bias in randomized trials was performed using the Cochrane Risk of Bias Tool for RCTs (24) by two investigators independently (Z.Y. and D.Z.), and studies were assessed from the following seven domains: random sequence generation, allocation concealment, blinding of participants and personnel, blinding of outcome assessment, incomplete outcome data, selective reporting, and other bias. Each domain was classified as low risk of bias, high risk of bias, or unclear risk of bias. Any disagreement was resolved by discussions with the third author (X.L.).

### 2.5. Data synthesis and statistical analyses

For continuous data, mean and standard deviation (SD) were extracted. For studies presenting median and interquartile range, mean was estimated by (first quartile + third quartile)/2, and SD was estimated by (third quartile − first quartile)/1.35 (25). For studies presenting 95% confidence intervals (CIs), standard error (SE) was estimated by (upper limit − lower limit)/3.92 and SD was calculated as SE × √n (26). After data extraction, we unified the unit of the outcomes previously reported, and the FBG, FINS, and the lipid markers levels (i.e., TGs, TC, HDL-C, LDL-C) were encoded in mmol/L, μIU/mL, and mg/dL, respectively.

#### 2.5.1. Pair-wise meta-analysis

First, we performed a pairwise meta-analysis for every intervention comparison. Continuous data were analyzed using Weighted mean differences (WMDs) and 95% CIs to express the effect size and *I^2^* statistic and *Q* test were used to assess the heterogeneity of the treatment effect which was deemed significant when *P* was <0.05 or *I^2^* was more than 50%. In this analysis, heterogeneity was present, thus, all results were reported using the random-effect model.

#### 2.5.2. Network meta-analysis

Second, network meta-analysis was performed using a random effects model based on the Bayesian framework and this model using the Markov-chain Monte Carlo (MCMC) method to obtain the non-informative uniform and normal prior distributions (27). Four iteration chains, with 50,000 iterations per chain, were set to fit the model and calculate the posterior distributions of model parameters. The thinning interval was set at 10 and the burn-ins at 1,000 for each chain. In this NMA, mean differences (MDs) with 95% credible intervals (CrIs) were generated from the posterior distribution medians, which did not contain 0 indicating significant differences between interventions. Deviance information criterion (DIC) was obtained from consistency and inconsistency models for each endpoint and difference between each pair of DICs (dDIC) were calculated to assess global inconsistency. A value of dDIC < 10 was deemed to have no appreciable global inconsistency. A node-split model was used to check the consistency assumption of direct evidence and indirect evidence with *p* < 0.05 indicating significant local inconsistency (28). The consistency model was adopted only if global inconsistency tests and node-split tests both reported no significant inconsistency. We performed meta-regression analysis to evaluate the potential impact of confounding factors (e.g., age, life style, proportion of male, total number of participants and intervention duration) on the model based on non-negligible differences in participant baseline characteristics (29). Surface under the cumulative ranking curve analysis (SUCRA) derived from posterior probabilities was used to rank the relative efficacy of interventions with larger SUCRA value indicating better interventions (30). Clustered-ranking plots were used for the determination of the most comprehensive intervention choice.

Stata software (version 12.0, StataCorp, College Station, TX) were used to produce the network evidence relationship plot and comparison-adjusted funnel plots. R software (version 3.6.2, MathSoftCorp, AT&T Bell Laboratories) with GeMTC (version 0.8-8) and JAGS packages (version 4.1.0, https://sourceforge.net/projects/mcmc-jags/files/) was used to perform the pairwise and network meta-analysis.

## 3. Results

### 3.1. Literature selection and study characteristics

**Figure 1:**
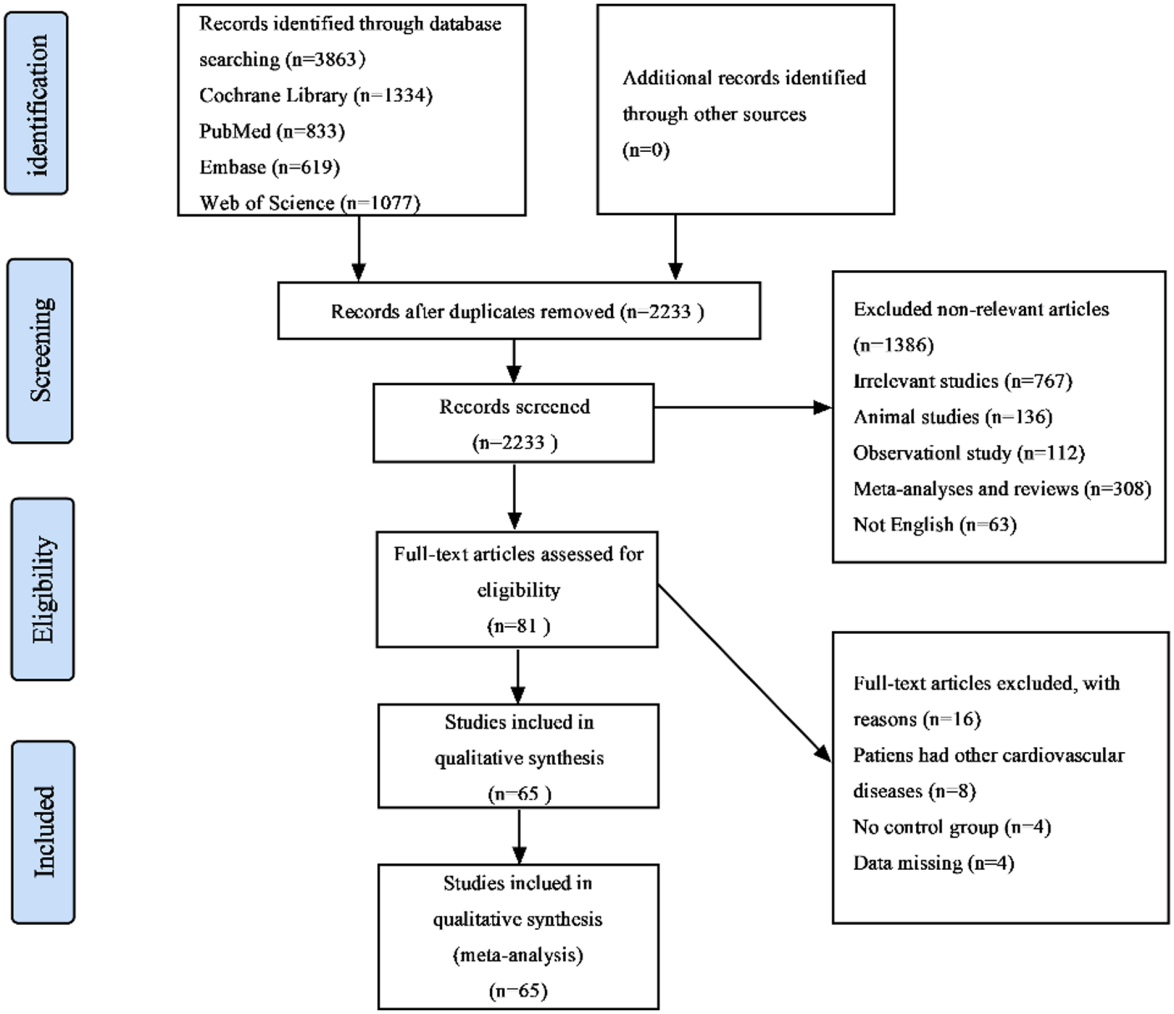
PRISMA flow diagram of selection of studies from search to final inclusion.

Of the 3,863 publications retrieved *via* literature search, 2,233 records left after removing duplicates. After reviewing the title and abstract, 81 studies were selected for further review. Then 16 studies were excluded (8 included patients with other cardiovascular diseases, 4 were without control group, and 4 did not meet our inclusion criteria). Finally, a total of 65 studies (31–95) and 4,241 obesity or overweight patients were ultimately included in this NMA, with 2,395 in the experimental group and 1,846 in the control group. The detailed selection process is described in Figure 1. All 65 included studies consist of 55 two-arms, 3 three-arms and 8 four-arms and were published between 2005 and 2022. The intervention duration of all studies was more than 4 weeks. The average age of the participants was 43.1 years and the percentage of male patients was about 40.7%. the average BMI of subjects is more than 30 kg/m^2^. Table 1 details the study characteristics.

**Table 1:** Characteristics of studies included in the network meta-analysis.

Row	Study	Year	Country	Sample size		Age mean (SD)/range	Male (%)	Intervention	Exercise	Diet control	Duration (week)	Outcomes
				T	C							
1	Arzola-Paniagua et al. (31)	2016	Mexico	15	24	33.7 (11.9)	15.4	Placebo vs. Resveratrol (300 mg) q.d.	NO	NO	24	a,b,c,d,e,j
						38.8 (9.6)						
2	Batista-Jorge et al. (32)	2020	Brazil	13	9	30–60	100	Placebo vs. Resveratrol (250 mg) q.d.	Yes	Yes	12	a,b,c,e,g,j,k,l,m
3	Kantartzis et al. (33)	2018	Germany	52	53	18–70	100	Placebo vs. Resveratrol (75 mg) b.i.d	NA	NA	12	d,f,g,h,i,j,k,l,m
4	Morten M (34)	2013	Denmark	11	14	44.7 (3.5)	100	Placebo vs. Resveratrol (500 mg) q.d.	No	No	4	d,e,f,g,j,k,l,m
						31.9 (2.9)						
5	Timmers et al. (35)	2011	Netherlands	11	11	52.5 (2.1)	100	Placebo vs. Resveratrol (150 mg) q.d.	No	No	4	d,e,h,i,j
						52.5 (2.1)						
6	Sanne M et al. (36)	2015	Italy	45	45	60.0 (7.0)	55.6	Placebo vs. Resveratrol (150 mg) q.d.	No	No	4	c,d,h,i,j,k
						60.0 (7.0)						
7	Wong et al. (37)	2013	Australia	15	13	61.0 (1.8)	42.9	Placebo vs. Resveratrol (75 mg) q.d.	No	No	6	a,h,i
						60.8 (1.8)						
8	Al-Bayyari et al. (38)	2018	Jordan	50	48	23.8 (0.6)	50	Placebo vs. VD (50,000 IU) q.w.	No	No	8	a,c
						23.3 (0.8)						
9	Carrillo et al. (39)	2013	America	10	13	26.2 (4.7)	47.8	Placebo vs. VD (4,000 IU) + Ca (500 mg) q.w.	Yes	No	12	d,e,f
						26.0 (4.5)						
10	Chandler et al. (40)	2014	America	81/83	81	51.1 (12.4)	NA	Placebo vs. VD (1,000 IU) vs. VD (2000 IU) q.d.	No	Yes	24	a,c
						50.3 (11.0)						
						51.3 (10.8)						
						50.7 (10.3)						
11	Cheshmazar et al. (41)	2020	Iran	30	25	38.3 (9.3)	33.9	Placebo vs. VD (2,000 IU) q.d.	Yes	Yes	8	a,b,c,d,j,k,l,m
						36.8 (7.7)						
12	Ebadi et al. (42)	2021	Iran	32	32	39.6 (12.7)	32.8	Placebo vs. VD (50,000 IU) q.w.	NA	NA	8	d,e,f,j,k,l,m
						37.0 (10.6)						
13	Farag et al. (43)	2018	Iran	21	25	40.4 (5.9)	56.5	Placebo vs. VD (200 IU) q.d.	Yes	No	12	a,b,c,d,h,i,j,k,l,m
						42.6 (5.6)						
14	Hajipoor (44)	2020	Iran	28/30/30	31	40.9 (6.8)	40	Placebo vs. VD (1,000 IU) vs. Probiotics (4 × 10^7^ CFU) vs. VD (1,000 IU) + Probiotics (4 × 10^7^ CFU) q.d.	Yes	Yes	10	a,b,c,j,k,l,m
						48.4 (9.7)						
						36.4 (21.1)						
						35.37 (11.69)						
15	Lithgow et al. (45)	2018	United Kingdom	10	10	34.0 (9.3)	70	Placebo vs. VD (4,000 IU) q.d.	Yes	No	6	a,b,c,d,e,f,h,i,j,k,l,m
						34.0 (10)						
16	Mai et al. (46)	2015	Italy	12	12	38.0 (2.4)	54.2	Placebo vs. VD (600,000 IU) at beginning	Yes	Yes	4	a,c,d,e,f,g
						37.0 (3.0)						
17	Major et al. (47)	2006	Canada	30	33	43.6 (5.0)	0	Placebo vs. VD (200 IU) + Ca (600 mg) b.i.d	No	Yes	15	a,b,c,d,e,h,i,j,k,l,m
						41.6 (6.1)						
18	Mason et al. (48)	2014	America	94	94	60.0 (5.3)	0	Placebo vs. VD (2,000 IU) q.d.	Yes	Yes	48	a,b,c
						59.6 (5.1)						
19	Rajaie et al. (49)	2018	Iran	20/21	19	39.3 (5.9)	50	Placebo vs. VD (200 IU) vs. VD (200 IU) + Ca (500 mg) b.i.d	No	Yes	8	a,b,c
						39.1 (6.1)						
						39.8 (5.5)						
20	Rajaie et al. (50)	2021	Iran	20/21	19	39.3 (5.9)	50	Placebo vs. VD (200 IU) vs. VD (200 IU) + Ca (500 mg) b.i.d	No	Yes	8	j,k,l,m
						39.1 (6.1)						
						39.8 (5.5)						
21	Makariou et al. (51)	2019	Greece	25	25	53.0 (7.0)	50	Placebo vs. VD (2,000 IU) q.d.	No	Yes	12	a,b,c
						52.0 (15.0)						
22	Makariou et al. (52)	2017	Greece	25	25	52.0 (9.0)	42	Placebo vs. VD (2,000 IU) q.d.	No	Yes	12	d,g,h,i,k,l,m
						51.0 (12.0)						
23	Salehpour et al. (53)	2012	Iran	39	38	38.0 (7.0)	0	Placebo vs. VD (25 μg) q.d.	Yes	No	12	a,b,c,h,i,j,k,l,m
						37.0 (8.0)						
24	Salekzamani et al. (54)	2016	Iran	35	36	40.5 (5.0)	100	Placebo vs. VD (5,000 IU) q.w.	NA	NA	16	a,b,c,d,e,f,h,i,j,k,l,m
						40.5 (5.0)						
25	Zittermann et al. (55)	2009	Germany	82	83	47.4 (10.3)	32.7	Placebo vs. VD (3,332 IU) q.d.	No	Yes	48	a,b,c,d,g,h,i,k,l,m
						43.7 (10.0)						
26	Huerta et al. (96)	2015	Spain	16/19/17	21	38.0 (7.0)	0	Placebo vs. α-lipoic acid (300 mg) vs. EPA (1,300 mg) vs. α-lipoic acid (300 mg) + EPA (1,300 mg) q.d.	No	Yes	10	a,c,d
						38.0 (8.0)						
						39.0 (7.0)						
						39.0 (8.0)						
27	Nasiri et al. (57)	2021	Iran	22/21/22	21	37.3 (7.6)	NA	Placebo vs. α-lipoic acid (600 mg) vs. Probiotics (2*10^11^ CFU) vs. α-lipoic acid (600 mg) + Probiotics (2*10^11^ CFU) q.d.	No	Yes	8	a,b,c,g,h
						34.7 (5.0)						
						34.9 (5.7)						
						34.3 (7.3)						
28	Romo-Hualde et al. (58)	2018	Spain	16/15/15	19	39.3 (6.6)	0	Placebo vs. α-lipoic acid 300 (mg) vs. EPA (1,300 mg) vs. α-lipoic acid (300 mg) + EPA (1,300 mg) q.d.	No	Yes	10	b,c,f,j,k,l
						37.2 (8.1)						
						38.1 (7.0)						
						39.0 (8.0)						
29	Bateni et al. (59)	2021	Iran	22	21	50.0 (9.0)	23.3	Placebo vs. Curcumin (80 mg) q.d.	No	No	12	a,b,c,d,e,f,g,l,i,g,k,l,m
						54.0 (7.0)						
30	Campbell et al. (60)	2019	America	10	10	18–35	100	Placebo vs. Curcumin (500 mg) q.d.	No	No	12	d,e,h,i
31	Cicero et al. (61)	2020	Italy	40	40	54.0 (3.0)	60.1	Placebo vs. Curcumin (800 mg) q.d.	Yes	Yes	8	b,c,d,e,f,h,i,j,k,l,m
						53.0 (5.0)						
32	Dolati et al. (62)	2020	Iran	10	10	38.9 (5.4)	0	Placebo vs. Curcumin (500 mg) q.d.	Yes	No	8	a,b,c,d,e,f,j,k,l,m
						40.8 (3.6)						
33	Javandoost et al. (63)	2018	Iran	36	36	18–35	NA	Placebo vs. Curcumin (100 mg) q.d.	No	Yes	6	e,j,k,l,m
34	Karandish et al. (64)	2021	Iran	21	20	37.0 (7.2)	31.7	Placebo vs. Curcumin (500 mg) q.d.	Yes	Yes	12	a,b,d,e,g
						34.2 (7.0)						
35	Mohammadi et al. (65)	2013	Iran	15	15	18–52	20	Placebo vs. Curcumin (1 g) q.d.	NA	NA	4	a,b,c,k,j,l,m
36	Yang et al. (66)	2014	China	30	29	59.3 (10.1)	46.8	Placebo vs. Curcumin (1,890 mg) q.d.	No	No	12	a,b,d,g,j,k,l,m
						59.6 (14.0)						
37	Abbott et al. (67)	2020	Australian	38	32	50.9 ± 12.7	63.2	Placebo vs. DHA (860 mg) + EPA (120 mg) q.d.	No	No	12	a,b,c,d,e,f,j,k,l,m
38	Baxheinrich et al. (68)	2012	Germany	40	41	52.3 (10.6)	31.6	Placebo vs. α-linolenic acid q.d.	No	Yes	26	a,b,c,d,e,h,i,j,k,l,m
						50.3 (9.8)						
39	Browning et al. (69)	2007	United Kingdom	18	18	NA	0	Placebo vs. DHA (2.9 g) + EPA (1.3 g) q.d.	No	No	12	d,e,h,i,j,k,l,m
40	de Luis et al. (70)	2016	Spain	14	15	47.4 (9.1)	41.4	Placebo vs. DHA (500 mg) q.d.	Yes	Yes	24	a,b,c,d,e,f,j,k,l,m
						44.3 (11.7)						
41	DeFina et al. (71)	2011	America	64	64	47.4 (9.1)	31.3	Placebo vs. DHA (2,500 mg) + EPA (500 mg) q.d.	Yes	Yes	24	a,b,c,d,e,h,i,j,l,m
						44.3 (11.7)						
42	Gammelmark et al. (72)	2012	Denmark	25	25	58.0 (7.4)	48	Placebo vs. DHA (480 mg) + EPA (640 mg) q.d.	No	No	6	b,c,d,g,j,k,l,m
						55.4 (9.5)						
43	Jaacks et al. (73)	2018	America	10	9	40–65	26.7	Placebo vs. DHA/EPA (1.8 g) q.d.	No	No	8	a,c,d,e,j,k,l,m
44	Kratz et al. (74)	2008	America	13	13	37.5 (14.0)	38.5	Placebo vs. Fish oil (diet) q.d.	No	Yes	16	a,d,e,f
						37.8 (13.6)						
45	Munro et al. (75)	2012	Australia	18	14	40.5 (10.9)	19.4	Placebo vs. DHA (1.62 g) + EPA (0.42 g) q.d.	No	Yes	14	a,b,c,d,j,k,l,m
						42.3 (9.10)						
46	Munro et al. (76)	2013	Australia	15	15	44.7 (2.7)	23.1	Placebo vs. DHA (1.62 g) + EPA (0.42 g) q.d.	No	Yes	8	a,b,c,d,j,k,l,m
						47.1 (2.5)						
47	Neale et al. (77)	2013	Australia	14	14	41.9 (11.7)	32.1	Placebo vs. DHA (744.9 mg) + EPA (1055.1 mg) q.d.	No	Yes	4	a,b,c,d,e
						42.0 (13.3)						
48	Rajkumar et al. (78)	2014	India	15/15/15	15	40–60	50	Placebo vs. DHA (120 mg) + EPA (180 mg) vs. Probiotics (112.5 × 10^9^ CFU) vs. DHA (120 mg) + EPA (180 mg) + Probiotics (112.5 × 10^9^ CFU) q.d.	No	No	6	d,j,k,l,m
49	Romo-Hualde et al. (79)	2016	America	10	10	30.0 (10.1)	100	Placebo vs. EPA (2.45 g) + DHA (1.61 g) + other n-3 (500 mg) q.d.	Yes	No	6	d,e,j,k,l,m
50	Sjoberg et al. (80)	2010	Australia	16/17/17	17	53.6 (2.5)	51	Placebo vs. DHA (0.52 g) vs. DHA (1.04 g) vs. DHA (1.56 g) q.d.	No	No	12	c,h,i
						63.4 (2.2)						
						54.0 (2.1)						
						54.0 (1.6)						
51	Anggeraini et al. (81)	2021	India	8	8	20.3 (0.7)	50	Placebo vs. Probiotics (1*10^9^ CFU) q.d.	No	No	8	a,c,d
						20.3 (0.7)						
52	Eslamparast et al. (82)	2014	Iran	19	19	47.5 (9.1)	39.4	Placebo vs. Probiotics (1*10^8^ CFU) q.d.	Yes	Yes	28	d,j,k,l,m
						46.1 (10.1)						
53	Hadi et al. (83)	2019	Iran	30	29	34.5 (6.0)	66.1	Placebo vs. Probiotics (2*10^9^ CFU) q.d.	Yes	Yes	8	a,b,c,d,e,h,i,j,k,l,m
						36.6 (7.3)						
54	Hess et al. (84)	2020	Denmark	59	57	48.9 (8.6)	45.3	Placebo vs. probiotics (1*10^8^ CFU) b.i.d	Yes	Yes	12	a,b,c,d,e,f,g,h,i,j,k,l,m
						48.2 (9.0)						
55	Rabiei et al. (85)	2019	Iran	20	20	57.1 (1.5)	26.1	Placebo vs. Probiotics (2*10^8^ CFU) q.d.	Yes	Yes	12	a,c,d,e,f,j,k,l,m
						60.8 (1.6)						
56	Rahayu et al. (86)	2021	Indonesia	30	30	44.1 (6.2)	40	Placebo vs. Probiotics (2*10^9^ CFU) q.d.	No	No	12	a,c,j,k,l,m
						44.7 (5.7)						
57	Szulińska et al. (87)	2018	Poland	23/24	24	55.2 (6.9)	0	Placebo vs. Probiotics (1*10^10^CFU) vs. Probiotics (2.5*10^9^CFU) q.d.	No	No	12	a,b,c,d,e,f,j,k,l,m
						56.4 (6.6)						
						58.7 (7.3)						
58	Tripolt et al. (88)	2013	Austria	13	15	51.0 (11.0)	35.7	Placebo vs. Probiotics (1.95*10^10^CFU) q.d.	No	No	12	c,d,e,f
						55.0 (9.0)						
59	Chacko et al. (89)	2011	America	7	7	47.0 (13.8)	69.2	Placebo vs. Mg (500 mg) q.d.	No	No	4	d,e,j
						41.9 (12.7)						
60	Joris et al. (90)	2016	Netherlands	26	25	62.0 (5.0)	47.1	Placebo vs. Mg (350 mg) q.d.	No	No	24	h,i
						62.0 (6.0)						
61	Joris et al. (91)	2017	Netherlands	26	25	62.0 (5.0)	47.1	Placebo vs. Mg (350 mg) q.d.	No	No	24	d,e,f,j,k,l,m
						62.0 (5.0)						
62	Lee et al. (92)	2009	Korea	75	80	39.6 (7.9)	49.7	Placebo vs. Mg (300 mg) q.d.	No	Yes	12	d,e,f,h,i,j,k,l,m
						40.5 (7.3)						
63	Mooren et al. (93)	2011	Germany	25	22	30–70	0	Placebo vs. Mg (365 mg) q.d.	No	No	24	c,d,e,f,h,i,j,k,l,m
64	Rodríguez-Moran et al. (94)	2014	Mexico	24	23	31.9 (5.6)	0	Placebo vs. Mg (382 mg) q.d.	No	Yes	16	b,c,d,e,f,h,i,j,l
						39.5 (8.3)						
65	Solati et al. (95)	2019	Iran	35	35	40.7 (11.9)	48.6	Placebo vs. Mg (300 mg) q.d.	No	No	24	c,d,e,f,j,k,l,m
						40.7 (12.7)						

T, Trail group; C, Control group; SD, Standard deviation; VD, vitamin D; Ca, calcium; Mg, magnesium.

Outcomes: a, Weight; b, Waist circumference (WC); c, Body mass index (BMI); d, Fasting blood glucose (FBG); e, Fasting insulin level (FINS); f, Homeostatic model assessment of insulin resistance (HOMA-IR); g, Hemoglobin A1c (HbA1c); h, Systolic blood pressure (SBP); i, Diastolic blood pressure (DBP); j, Triglycerides (TGs); k, Total cholesterol (TC); l, High-density lipoprotein cholesterol (HDL-C); m, Low-density lipoprotein cholesterol.

**Figure 2:**
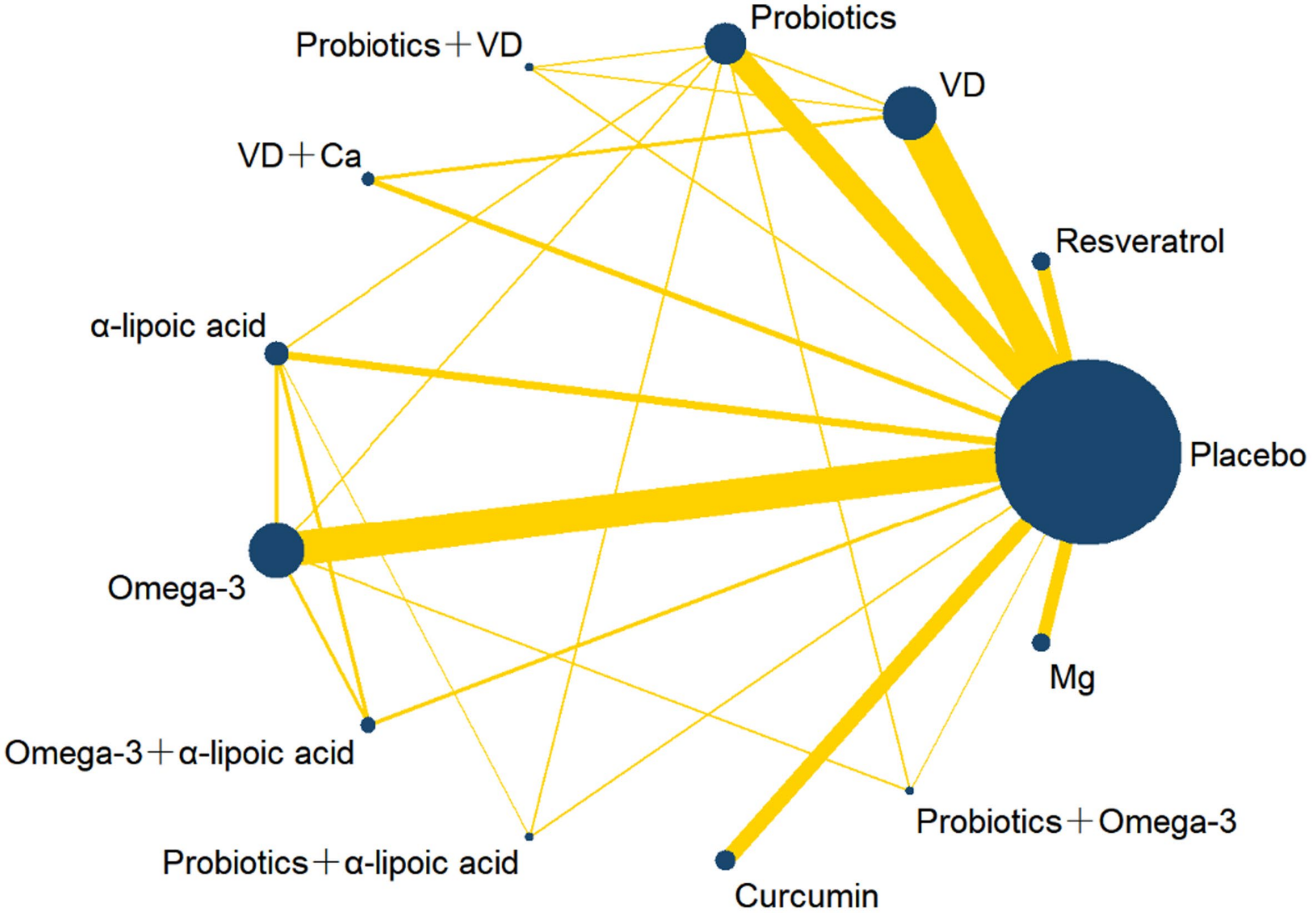
Network plot of different nutrition supplements for overweight and obese treatment. The width of the line is directly proportional to the number of treatments for each pair; the area of the circle represents the cumulative number of patients per intervention. VD, Vitamin D; Ca, Calcium; Mg, Magnesium.

Figure 2 shows the network plots of the included studies. We included 13 kinds of nutritional supplementations in our NMA: resveratrol, VD, probiotics, probiotics + VD, VD + Ca, α-lipoic acid, omega-3, omega-3 + α-lipoic acid, probiotics + α-lipoic acid, curcumin, probiotics + omega-3, Mg, and placebo.

### 3.2. Risk of bias and data quality

The results of quality assessment were summaries in Supplementary Figure S1. The revised Cochrane Risk-of bias Tool for RCTs (RoB 2.0) was used to assess the quality of 65 included RCTs. All eligible RCTs mentioned randomization and were classified as “low risk.” 28 articles showed “unclear risk” and 1 article showed “high risk” in adequate allocation concealment. Six articles showed “unclear risk” and 4 articles showed “high risk” in blinding of participants and personnel. 28 articles showed “unclear risk” and 1 article showed “high risk” in terms of adequate allocation concealment. Six articles showed “unclear risk” and 6 articles showed “high risk” in the aspect of blinding of outcome assessment. For complete outcome assessment and selective reporting, 22 articles were deemed as “unclear risk” in complete data, whereas 65 articles showed no selecting outcomes to report. The 22 articles were considered as “unclear risk” in other bias.

### 3.3. Exploration of inconsistency

Across all primary and secondary outcomes, model fit and iteration convergence were both good. All the dDIC value of the outcomes are less than 5 and the *I^2^* value of all outcomes were less than 25%, indicating that there is no significant difference between the global consistency model and inconsistency model (Supplementary Table S2). There are some closed-loop network structures in the comparison of Weight, WC, BMI, FBG, FINS, TGs, TC, HDL-C, LDL-C, and no inconsistency between direct and indirect evidence was found by node-splitting method (all *p* > 0.05 in Supplementary Table S3). Network meta-regression showed no association among our all outcomes and life styles, proportion of male, total number of participants and intervention duration; however, we found some potential heterogeneity in the mean age of the patients with respect to weight and WC (Supplementary Figure S2).

### 3.4. Cardiovascular risk factors

#### 3.4.1. Blood pressure

The change in blood pressure was recorded in 7 studies with 1787 patients. Pairwise meta-analysis and NMA results both revealed that there was no significant difference in SDP and DBP change in all the 9 interventions and placebo (Supplementary Tables S1, S4). The rankings were shown in Table 2 and Supplementary Figures S3A,B.

**Table 2:** Surface under the cumulative ranking curve and ranking probability of different nutrition supplements on each outcome.

Interventions	SBP		DBP		FBG		FINS		HOMA-IR		HbA1c			
	SUCRA (%)	Rank	SUCRA (%)	Rank	SUCRA (%)	Rank	SUCRA (%)	Rank	SUCRA (%)	Rank	SUCRA (%)		Rank	
Placebo	26.5	10	47.1	7	33.0	11	33.0	11	35.9	7	49.5		4	
Resveratrol	34.4	8	50.9	5	40.5	8	40.6	8	39.2	6	43.8		5	
VD	39.1	7	48.6	6	48.1	4	48.3	4	53.9	4	28.3		7	
Probiotics	57.0	4	61.8	3	***90.4***	1	***90.5***	1	***92.4***	1	***73.4***		1	
Probiotics + VD	NA	NA	NA	NA	NA	NA	NA	NA	NA	NA	NA		NA	
VD + Ca	42.8	6	47.1	8	41.3	7	41.4	7	NA	NA	NA		NA	
α-lipoic acid	67.8	3	59.4	4	36.0	10	35.8	10	32.3	8	NA		NA	
Omega-3	69.7	2	11.3	10	39.74	9	39.7	9	18.7	9	51.4		3	
Omega-3 + α-lipoic acid	NA	NA	NA	NA	47.14	5	46.9	5	51.2	5	NA		NA	
Probiotics + α-lipoic acid	***79.3***	1	***67.7***	1	NA	NA	NA	NA	NA	NA	NA		NA	
Curcumin	34.3	9	38.7	9	45.67	6	45.6	6	66.4	2	71.6		2	
Probiotics + omega-3	NA	NA	NA	NA	79.85	2	79.8	2	NA	NA	NA		NA	
Mg	44.6	5	67.4	2	48.40	3	48.5	3	59.9	3	32.0		6	
Interventions	TGs		TC		HDL-C		LDL-C		Weight		WC		BMI	
	SUCRA (%)	Rank	SUCRA (%)	Rank	SUCRA (%)	Rank	SUCRA (%)	Rank	SUCRA (%)	**Rank**	SUCRA (%)	**Rank**	SUCRA (%)	Rank
Placebo	13.7	12	35.7	9	34.8	9	69.4	2	27.9	10	37.9	9	40.1	11
Resveratrol	31.2	11	40.8	7	22.6	11	35.8	10	49.8	7	51.6	5	45.1	6
VD	39.1	9	***81.2***	1	65.9	4	67.6	3	50.6	5	53.1	4	45.3	5
Probiotics	57.0	5	59.8	3	51.2	5	36.4	9	67.1	2	69.6	2	49.0	4
Probiotics + VD	51.9	7	53.63	5	47.2	6	32.9	11	53.9	4	26.4	12	42.4	9
VD + Ca	58.5	4	61.5	2	47.1	7	40.8	8	44.9	8	35.1	11	44.0	7
α-lipoic acid	***75.2***	1	NA	NA	22.0	12	52.6	5	57.6	3	35.2	10	84.8	2
Omega-3	50.5	8	38.7	8	33.9	10	***77.2***	1	26.7	11	43.1	8	11.8	12
Omega-3 + α-lipoic acid	66.7	3	NA	NA	43.2	8	50.8	6	50.5	6	47.7	6	***90.7***	1
Probiotics + α-lipoic acid	NA	NA	NA	NA	NA	NA	NA	NA	***79.4***	1	***83.5***	1	62.9	3
Curcumin	34.9	10	25.8	10	74.5	2	66.5	4	41.6	9	69.5	3	42.47	8
Probiotics + omega-3	67.3	2	57.1	4	***90.1***	1	21.2	12	NA	NA	NA	NA	NA	NA
Mg	54.1	6	45.8	6	67.5	3	49.0	7	NA	NA	47.3	7	41.44	10

Bold and inclined: the SUCRA is relatively higher when compared with other interventions. Ranking: the probability of being the best, the second best, or the third best treatment, and so on, among all treatments. Rank 1 is the best, and Rank N is the worst. SBP, systolic blood pressure; DBP, diastolic blood pressure; FBG, fasting blood glucose; FINS, fasting insulin level; HOMA-IR, homeostatic model assessment of insulin resistance; HbA1c, hemoglobin A1c; TGs, triglycerides; TC, total cholesterol; HDL-C, high-density lipoprotein cholesterol; LDL-C, low-density lipoprotein cholesterol; WC, waist circumference; BMI, body mass index; SUCRA, surface under the cumulative ranking curve; VD, vitamin D; Ca, calcium; Mg, magnesium.

#### 3.4.2. Glucose and lipid metabolism

FBG was measured in 39 studies and involved 2,731 patients. The pairwise meta-analysis revealed that compared with placebo, probiotics + omega-3 (WMD: −5.55 mmol/l; 95%CI: −6.69 to −4.40) resulted in a significant reduction in FBG (Supplementary Table S1). However, in NMA (Table 3), compared with placebo (MD: −0.90 mmol/L; 95%CrI: −1.41 to −0.38), VD (MD: −0.75 mmol/L; 95%CrI: −1.47 to −0.01), and omega-3 (MD: −0.84 mmol/L; 95%CrI: −0.20 to −1.47), probiotics resulted in a greater reduction in FBG. According to the SCURA values, probiotics (SUCRA 90.4%) may be the best intervention for decreasing FBG (Table 2; Supplementary Figure S3C).

**Table 3:** Results of the network meta-analysis of FINS (lower-left quadrant) and FBG (upper-right quadrant).

**FBG**													
**FINS**	**Placebo**	−0.06 (−0.74, 0.62)	−0.15 (−0.67, 0.37)	**−0.90 (−1.41, −0.38)**	—	−0.02 (−1.54, 1.50)	0.07 (−1.28, 1.41)	−0.06 (−0.47, 0.35)	−0.14 (−1.49, 1.21)	—	−0.12 (−0.73, 0.49)	−0.82 (−2.07, 0.43)	−0.16 (−0.8, 0.48)
	−0.31 (−2.81, 2.11)	**Resveratrol**	−0.09 (−0.95, 0.77)	−0.84 (−1.69, 0.02)	—	0.04 (−1.62, 1.72)	0.13 (−1.39, 1.64)	0.00 (−0.79, 0.79)	−0.08 (−1.60, 1.43)	—	−0.06 (−0.98, 0.85)	−0.76 (−2.19, 0.66)	−0.1 (−1.03, 0.83)
	−0.17 (−2.68, 2.27)	0.14 (−3.35, 3.62)	**VD**	**−0.75 (−1.47, −0.01)**	—	0.13 (−1.47, 1.74)	0.22 (−1.23, 1.66)	0.09 (−0.57, 0.76)	0.01 (−1.44, 1.46)	—	0.03 (−0.78, 0.83)	−0.67 (−2.03, 0.69)	−0.01 (−0.83, 0.82)
	**−2.05 (−4.27, −0.02)**	−1.73 (−5.06, 1.44)	−1.88 (−5.17, 1.31)	**Probiotics**	—	0.88 (−0.72, 2.47)	0.96 (−0.48, 2.40)	**0.84 (0.20, 1.47)**	0.76 (−0.69, 2.20)	—	0.77 (−0.03, 1.57)	0.08 (−1.21, 1.36)	0.74 (−0.08, 1.55)
	—	—	—	—	**Probiotics + VD**	—	—	—	—	—	—	—	—
	−0.39 (−5.89, 5.09)	−0.08 (−6.08, 5.95)	−0.22 (−6.22, 5.79)	1.64 (−4.17, 7.63)	—	**VD + Ca**	0.09 (−1.94, 2.11)	−0.04 (−1.61, 1.54)	−0.13 (−2.18, 1.9)	—	−0.11 (−1.74, 1.53)	−0.81 (−2.77, 1.16)	−0.15 (−1.79, 1.5)
	—	—	—	—	—	—	**α-lipoic acid**	−0.13 (−1.47, 1.23)	−0.21 (−1.75, 1.34)	—	−0.19 (−1.68, 1.29)	−0.89 (−2.72, 0.94)	−0.23 (−1.72, 1.27)
	−0.52 (−2.25, 1.14)	−0.21 (−3.2, 2.78)	−0.35 (−3.34, 2.64)	1.54 (−0.95, 4.17)	—	−0.10 (−5.85, 5.62)	—	**omega-3**	−0.08 (−1.43, 1.27)	—	−0.07 (−0.8, 0.67)	−0.76 (−2.04, 0.51)	−0.1 (−0.86, 0.65)
	—	—	—	—	—	—	—	—	**omega-3 + α-lipoic acid**	—	0.02 (−1.46, 1.5)	−0.68 (−2.5, 1.16)	−0.02 (−1.51, 1.47)
	—	—	—	—	—	—	—	—	—	**Probiotics + α-lipoic acid**	—	—	—
	−0.97 (−3.41, 1.92)	−0.66 (−4.02, 3.22)	−0.79 (−4.20, 3.11)	1.08 (−2.09, 4.87)	—	−0.56 (−6.44, 5.76)	—	−0.46 (−3.38, 2.98)	—	—	**Curcumin**	−0.7 (−2.09, 0.7)	−0.04 (−0.92, 0.85)
	**6.68 (2.65, 10.63)**	**7.00 (2.29, 11.68)**	**6.86 (2.15, 11.54)**	**8.72 (4.61, 12.92)**	—	**7.09 (0.28, 13.82)**	—	**7.20 (3.06, 11.25)**	—	—	**7.66 (2.51, 12.19)**	**Probiotics + omega-3**	0.66 (−0.74, 2.06)
	−0.63 (−2.84, 1.46)	−0.32 (−3.61, 2.91)	−0.46 (−3.76, 2.78)	1.41 (−1.59, 4.48)	—	−0.23 (−6.16, 5.63)	—	−0.13 (−2.90, 2.58)	—	—	0.33 (−3.41, 3.50)	**−7.32 (−11.89, −2.75)**	**Mg**

Data are reported as mean difference (95% credible intervals) and indicate column-to-row differences (i.e., compared with placebo, prebiotic reduces FBG by 0.90 mmol/l). Statistically significant differences are in bold and inclined formats (*p*-values < 0.05). FBG, Fasting blood glucose; FINS, Fasting insulin level; VD, Vitamin D; Ca, Calcium; Mg, Magnesium. Statistically significant effects are shown in bold and underline.

FINS was measured in 37 studies involving 1936 patients. The pairwise meta-analysis revealed that compared with placebo, α-lipoic acid + probiotics (WMD: −2.51 μIU/mL; 95%CI: −3.33 to −1.69) and probiotics + omega-3 (WMD: −4.04 μIU/mL; 95%CI: −4.94 to −3.14) resulted in a significant reduction in FINS (Supplementary Table S1). However, NMA revealed that probiotics might be more effective than placebo (MD: −2.05 μIU/mL; 95%CrI: −4.27 to −0.02) and probiotics + omega-3 (MD: −8.72 μIU/mL; 95% CrI: −4.61 to −12.92; Table 3). According to the SCURA values, probiotics (SUCRA 90.5%) may be the best intervention to reduce FINS (Table 2; Supplementary Figure S3D).

HOMA-IR was measured in 25 studies involving 1,436 patients. The pairwise meta-analysis revealed that compared with placebo, α-lipoic acid + probiotics (WMD: −2.59; 95%CI: −3.42 to −1.76) and curcumin (WMD: −0.41; 95%CI: −0.74 to −0.08, *I^2^* = 0%; *p* = 0.60) resulted in a greater benefit in improving HOMA-IR (Supplementary Table S1). However, NMA showed that probiotics might be more effective than placebo (MD: −1.43; 95%CrI: −2.46 to −0.31) and omega-3 (MD: −1.92; 95%CrI: −0.20 to −3.55; Supplementary Table S5). According to the SCURA values, probiotics (SUCRA 93.4%) may be the best intervention to improve HOMA-IR (Table 4; Supplementary Figure S3E).

**Table 4:** Results of the network meta-analysis of TC (lower-left quadrant) and TGs (upper-right quadrant).

**TGs**													
**TC**	**Placebo**	−4.01 (−18.50, 11.34)	−6.39 (−19.11, 5.69)	**−11.15 (−22.16, −1.26)**	−10.63 (−42.58, 21.49)	−12.9 (−37.04, 11.16)	−21.44 (−52.74, 9.12)	**−9.45 (−20.69, −0.93)**	−16.99 (−47.27, 12.45)	—	−4.70(−23.13, 9.62)	−14.94 (−35.44, 3.63)	−11.14 (−25.30, 7.47)
	−0.74 (−28.19, 26.54)	**Resveratrol**	−2.40 (−22.61, 16.46)	−7.20 (−26.38, 10.19)	−6.65 (−42.23, 28.37)	−8.95 (−37.64, 19.07)	−17.4 (−52.62, 16.25)	−5.52 (−25.16, 11.22)	−12.90 (−47.20, 19.66)	—	−0.78(−25.41, 19.25)	−11.00 (−36.87, 12.21)	−7.14 (−27.48, 16.53)
	**−17.86 (−35.53, −0.27)**	−17.14 (−49.64, 15.42)	**VD**	−4.82 (−20.57, 10.89)	−4.26 (−36.47, 28.88)	−6.53 (−31.34, 18.85)	−15.00 (−48.28, 18.16)	−3.08 (−19.49, 11.74)	−10.59 (−42.92, 21.55)	—	1.67 (−20.15, 20.05)	−8.74 (−31.83, 14.04)	−4.66 (−23.32, 18.61)
	−8.26 (−25.29, 8.78)	−7.55 (−39.48, 24.88)	9.59 (−13.89, 33.28)	**Probiotics**	0.64 (−31.91, 33.58)	−1.65 (−27.56, 24.77)	−10.29 (−42.63, 22.07)	1.83 (−12.43, 14.04)	−5.77 (−37.18, 25.36)	—	6.61 (−14.52, 23.66)	−3.89 (−24.20, 16.17)	0.07 (−16.85, 22.37)
	−7.12 (−49.72, 35.57)	−6.41 (−56.69, 44.45)	10.81 (−32.48, 54.18)	1.09 (−42.40, 44.56)	**Probiotics + VD**	−2.28 (−41.97, 37.15)	−10.79 (−55.43, 33.21)	1.09 (−33.12, 33.98)	−6.36 (−50.51, 37.37)	—	5.8 (−31.93, 40.87)	−4.53 (−41.94, 31.93)	−0.22 (−35.19, 37.08)
	−10.83 (−46.44, 24.42)	−10.11 (−55.03, 34.80)	7.01 (−30.47, 44.46)	−2.61 (−41.91, 36.35)	−3.77 (−58.74, 50.90)	**VD + Ca**	−8.50 (−47.93, 30.28)	3.38 (−23.55, 28.45)	−3.94 (−42.64, 33.83)	—	8.21 (−22.68, 35.42)	−2.20 (−33.59, 28.16)	1.88 (−25.69, 32.92)
	—	—	—	—	—	—	**α-lipoic acid**	11.90 (−19.65, 42.29)	4.51 (−32.06, 40.78)	—	16.65 (−19.71, 50.13)	6.26 (−29.60, 42.18)	10.44 (−22.96, 47.62)
	−0.56 (−18.49, 17.47)	0.20 (−32.22, 33.05)	17.32 (−7.77, 42.41)	7.70 (−15.90, 31.55)	6.58 (−39.46, 52.75)	10.29 (−29.38, 50.19)	—	**omega-3**	−7.43 (−36.61, 22.89)	—	4.91 (−14.63, 21.80)	−5.96 (−24.61, 15.12)	−1.42 (−18.39, 21.44)
	—	—	—	—	—	—	—	—	**omega-3 + α-lipoic acid**	—	12.19(−23.09, 44.64)	1.77 (−32.98, 36.70)	5.97 (−26.47, 42.48)
	—	—	—	—	—	—	—	—	—	**Probiotics + α-lipoic acid**	—	—	—
	5.39 (−16.84, 27.37)	6.14 (−29.00, 41.42)	23.26 (−5.00, 51.53)	13.61 (−14.34, 41.46)	12.49 (−35.29, 60.1)	16.26 (−25.59, 57.97)	—	5.95 (−22.66, 34.44)	—	—	**Curcumin**	−10.80 (−33.33, 16.90)	−6.11 (−27.15, 21.72)
	−9.21 (−51.53, 33.41)	−8.51 (−58.83, 42.4)	8.67 (−37, 54.7)	−0.95 (−44.19, 42.53)	−2.13 (−61.73, 57.69)	1.64 (−53.31, 57.06)	—	−8.65 (−52.08, 34.86)	—	—	−14.55 (−62.30, 33.58)	**Probiotics + omega-3**	3.82 (−18.52, 32.83)
	−2.94 (−29.3, 23.75)	−2.21 (−40.05, 36.09)	14.96 (−16.72, 46.85)	5.34 (−26.07, 37.17)	4.16 (−45.99, 54.31)	7.91 (−36.04, 52.31)	—	−2.34 (−34.52, 29.74)	—	—	−8.29 (−42.65, 26.33)	6.30 (−43.67, 56.44)	**Mg**

Data are reported as mean difference (95% credible intervals) and indicate column-to-row differences. Statistically significant differences are in bold and inclined formats (*P*-values < 0.05). TGs, Triglycerides; TC, Total cholesterol; VD, Vitamin D; Ca, Calcium; Mg, Magnesium. Statistically significant effects are shown in bold and underline.

HbA1c was reported in 12 studies involving 724 patients. The pairwise meta-analysis revealed that only curcumin (WMD: −0.36; 95%CI: −0.70 to −0.01; *I^2^* = 16%; *p* = 0.30) resulted in a significant reduction in HbA1c compared to placebo (Supplementary Table S1). However, there was no significant difference between all interventions and placebo in NMA (Supplementary Table S5).

TGs was measured in 43 studies involving 2,795 patients. The pairwise meta-analysis revealed that compared with placebo, probiotics (WMD: −0.21 mg/dL; 95%CI −0.39 to −0.04; *I^2^* = 49%; *p* = 0.10), omega-3 (WMD: −0.29 mg/dL; 95%CI −0.46 to −0.13; *I^2^* = 0%; *p* = 0.89) and probiotics + omega-3 (WMD: −0.60 mg/dL; 95%CI: −1.12 to −0.09) all resulted in a significant reduction in TGs (Supplementary Table S1). Similarly, NMA revealed that probiotics (MD: −11.15 mg/dL; 95%CrI: −22.16 to −1.26) and omega-3 (MD: −9.45 mg/dL; 95%CrI: −20.69 to −0.93) might be more effective than placebo (Table 4). According to the SCURA values, α-lipoic acid (SUCRA 75.2%) may be the best intervention to reduce TGs (Table 4; Supplementary Figure S3G).

TC was measured in 37 studies involving 2,379 patients. The pairwise meta-analysis showed that compared with placebo, probiotics (WMD: −0.36 mg/dL; 95%CI: −0.57 to −0.15; *I^2^* = 23%; *p* = 0.24), α-lipoic acid + probiotics (WMD: −2.51 mg/dL; 95%CI: −3.33 to −1.69), and probiotics + omega-3 (WMD: −0.94 mg/dL; 95%CI: −1.48 to −0.41) all resulted in a significant reduction in TC (Supplementary Table S1). However, NMA revealed that only VD (MD: −17.86 mg/dL; 95%CrI: −35.53 to −0.27) might be more effective than placebo (Table 4). According to the SCURA values, VD (SUCRA 81.2%) may be the best intervention to reduce TC (Table 2; Supplementary Figure S3H).

HDL-C was reported in 43 studies involving 2,804 patients. The pairwise meta-analysis demonstrated that curcumin (WMD: 0.35 mg/dL; 95%CI: 0.12 to 0.57; *I^2^* = 0%; *p* = 0.70) and probiotics + omega-3 (WMD: 3.07 mg/dL; 95%CI: 2.31 to 3.83) resulted in a significant increase in HDL-C compared to placebo (Supplementary Table S1). Likewise, NMA revealed that probiotics + omega-3 might be more effective than placebo (MD: 5.09 mg/dL; 95%CrI: 0.77 to 9.38), resveratrol (MD: 6.36 mg/dl; 95%CrI: 0.92 to 11.58) and omega-3 (MD: 5.24 mg/dL; 95%CrI 0.78 to 9.63; Supplementary Table S6). According to the SCURA values, probiotics + omega-3 (SUCRA 90.1%) may be the best intervention to increase HDL-C (Table 2; Supplementary Figure S3I).

LDL-C was reported in 42 studies involving 2,737 patients. The pairwise meta-analysis described that compared with placebo, probiotics (WMD: −0.32 mg/dL; 95%CI: −0.52 to −0.12; *I^2^* = 25%; *p* = 0.22) and probiotics + omega-3 (WMD: −1.37 mg/dL; 95%CI: −1.94 to −0.80) resulted in a significant reduction in LDL-C (Supplementary Table S1). However, there was no significant difference between all interventions and placebo in NMA (Supplementary Table S6).

**Figure 3:**
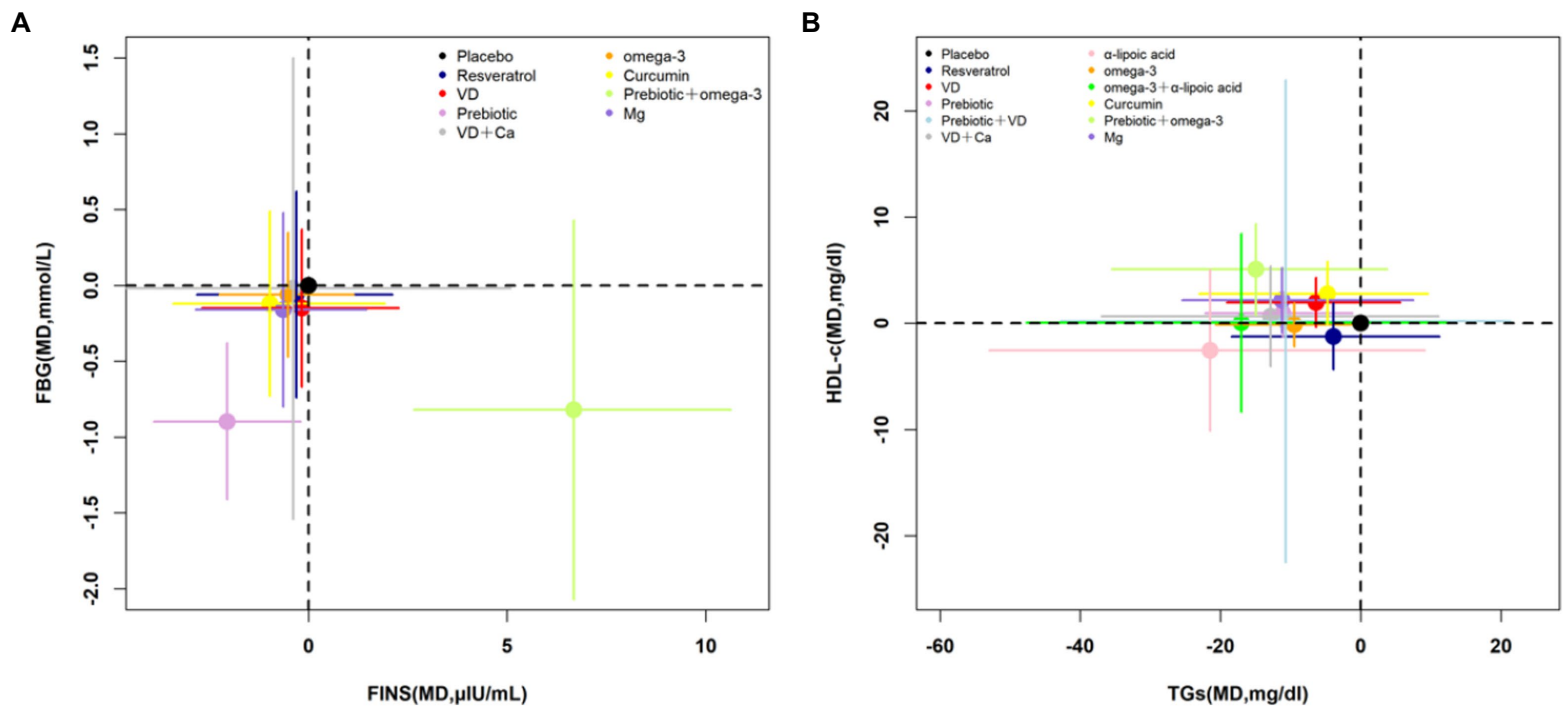
Cluster-rank plots. **(A)** The cluster-rank plot of FBG and FINS. **(B)** The cluster-rank plot of HDL-C and TGs. (The cluster-rank value is the product of the abscissa and ordinate of each treatment.) FBG, fasting blood glucose; FINS, fasting insulin level; TGs, triglycerides; TC, total cholesterol; HDL-C, high-density lipoprotein cholesterol; VD, vitamin D; Ca, calcium; Mg, magnesium.

According to the cluster-rank results in Figure 3, probiotics ranked highest in decreasing the FBG and FINS and was the optimum strategy (cluster-rank value = 3290.50); probiotics + omega-3 ranked highest in decreasing the TGs meanwhile increasing the HDL-C and has the greatest potential to be the optimum strategy (cluster-rank value = 2117.61).

### 3.5. Body composition

In this NMA, body weight, WC and BMI were reported in 38, 33, and 45 studies, respectively. The results of this NMA showed that there was no significant difference in weight loss and WC reduction among all interventions. The effect of omega-3 + α-lipoic acid (MD: −6.70 Kg/m^2^; 95%CI: −13.13 to −0.24) on reducing BMI was significantly better than that of placebo (Supplementary Table S8). According to the SCURA values, α-lipoic acid + probiotics might be the best intervention to reduce body weight (SUCRA 79.4%) and WC (SUCRA 83.5%), and omega-3 + α-lipoic acid (SUCRA 90.7%) might be the best intervention to reduce BMI (Table 2; Supplementary Figures S3K–M).

### 3.6. Comparison-adjusted funnel plot

Comparison-adjusted funnel plot was showed in Supplementary Figure S4. All studies on the funnel plot were symmetrically distributed on either side of the vertical line of *X* = 0, indicating that there was no significant small sample effects or publication bias.

## 4. Discussion

At present, nutritional supplements have been shown to be effective as a complementary therapy to improve glucose and lipid metabolism in patients with metabolic diseases (97, 98). However, the effects of common nutritional supplements on improving cardiometabolic risk factors in overweight and obese patients are inconsistent. Network meta-analysis can directly and indirectly compare the efficacy of various nutritional supplements to determine the best nutritional strategy (99).

Overall, among the interventions we included in the comparison, probiotic showed significantly higher efficacy in reducing FBG, FINS, and HOMA-IR than placebo and other interventions; probiotic and omega-3 seemed to be more effective than placebo and other nutrients in reducing TGs; probiotic + omega-3 seemed to be more effective than placebo and other nutrients in increasing HDL-C; however, none of the interventions had a significant impact on body weight, WC, and BMI.

In terms of blood glucose metabolism, SUCRA shows that probiotic was the best way for improving the FBG, FINS, and HOMA-IR. The result is inconsistent with Zarezadeh, who believes that probiotics supplementation does not reduce glucose metabolism in patients with metabolic syndrome and obesity (97). We believe that this is mainly due to the difference in intervention dose and duration. The study intervention included in our meta lasted 8 weeks, and were all medium dose probiotics (more than 10^8^ or 10^9^ CFU). Our NMA also provides compelling evidence for the benefits of probiotics in improving blood glucose metabolism. Different studies have explained the potential hypoglycemic mechanisms of probiotics, and most studies believe that it may be related to gut bacteria, increasing satiety and reducing appetite (13, 100–102). A variety of probiotics, such as *Bifidobacterium and Lactobacillus,* were used in the included studies. These composite probiotics can decrease the number of harmful bacteria such as *Acinobacteria*, *Escherichina*, and *Gram-negative bacteria*, and promote the growth of short chain fatty acids (SCFAs) (103). SCFAs play an important role in regulating glucose storage in the muscle, adipose tissue, and liver (104). Moreover, probiotics can improve insulin signaling pathway (105).

In terms of lipid metabolism, NMA shows that probiotic and omega-3 seemed to be more effective than placebo in reducing TGs, however, the SUCRA shows that α-lipoic acid might be the most successful intervention among these treatments. This research showed that α-lipoic acid reduced triglycerides to a greatest extent. α-lipoic acid is a potent antioxidant and free radical scavenger, but the mechanism of its regulation of blood lipids is still unclear (106). Butler et al. found that α-lipoic acid offset the rise of TGs in blood and liver by inhibiting liver lipogenic gene expressions, and stimulate clearance of TG-rich lipoproteins by lowering the secretion of hepatic TGs (107). Lee WJ ‘s experimental study showed that α-lipoic acid decreased lipid accumulation in skeletal muscle and hepatic steatosis by activating AMP-activated kinase (AMPK) (108). The SCURA shows that VD was the best for decreasing TC. Makariou (51) and Jiang XJ (109) found a significant negative correlation between VD supplementation and TC. Jorde et al. found that vitamin D 40,000 IU/wk. did not significantly improve serum lipids and other cardiovascular risk factors compared with placebo (110). In contrast, Ramiro-Lozano and Calvo-Romero presented that oral vitamin D 16,000 IU/wk. showed a reduction in TC but not LDL-C and TGs in type 2 diabetes participants (111). In this NMA, the doses of vitamin D in most studies exceeded 2,000 IU/d, which may prove that high VD levels were associated with a favorable serum lipid profile. With regard to cholesterol level, Major’s experiment study showed that vitamin D may increase calcium intestinal absorption and insoluble calcium-fatty soap formation in the gut, resulting in reduced fatty acid absorption and increased fecal fatty acids (47). The effect of probiotics +omega-3 on HDL-C was significant, and SCURA shows that probiotics +omega-3 might be the most successful intervention. The results on HDL-C increasing are compatible with those of Jone’s (112) and Venturini’s (113) experiment studies, but discordant with those of Chang’s (114, 115), possibly owing to the different proportions and dose of omega-3. The effect of omega-3 on HDL cholesterol has been well established (116), but the mechanism of synergistic effect in combination with probiotics remains unclear.

This study comprehensively analyzed the effect of intervention on blood glucose control and lipid metabolism, and the results of cluster rank analysis were consistent with those of SUCRA. Probiotic was found to have statistically significant advantages in decreasing FBG and FINS simultaneously. For the effect of lowering TGs and improving HDL-C, the cluster rank analysis showed that omega-3 + probiotics might be the most effective intervention. Previous meta-analysis results showed different nutrients have different effects on body composition in obese and overweight patients (117, 118). In this NMA, no effective change was found in body weight, WC, and BMI, however, meta-regression shows that age may be a source of heterogeneity in the results of body weight and WC. A low-calorie diet and regular physical exercise were also important ways to improve cardiometabolic indicators in the early prevention of overweight and obesity patients, but meta-regression indicated that the results were consistent, and no matter the daily exercise or a low-calorie diet alone or a combination of the two life styles had no significant effect on the outcomes.

In this review, chronic cardiovascular diseases with complex pathogenesis are excluded, only overweight and obese patients are included, which reduces clinical heterogeneity to some extent and improves the comparability of results. A comprehensive search of treatment strategies for common nutritional supplements in adjuvant therapy was conducted, including a sufficient number of RCT experiments and nutrients were compared as much as possible. The statistical stability and reliability of network meta-analysis depends on the uniform standards of similarity, homogeneity and consistency. No inconsistency was observed in this NMA through consistency test and node splitting method, and the NMA results are robust. Based on SCURA and cluster rank analysis, the results of this NMA will be useful for clinical decision making.

## 5. Limitations

There are also some limitations shown in the study. First, we did not perform further subgroup analysis. On the one hand, subgroups could not include all supplements in this study. On the other hand, this meta-regression showed the reported results have good consistency, which was not affected by the imputation models. Second, although all the included studies are RCTs, the most common; used is a placebo as a control. Due to the variety of interventions, a small number of direct comparisons of some treatments impairs the robustness of the final results. Third, although SCURA ranking has been widely used to give clinically significant results, due to the minimum absolute difference between the highest rank and others rankings, cautious interpretation is required.

## 6. Conclusion

Nutrition supplements might be positive efficacious intervention from which patients with overweight or obesity will derive benefit in improving some CVD risk factors. Probiotics supplementation might be potentially the preferred the intervention for glycemic control. VD, α-lipoic acid, probiotic + omega-3 have a better impact on lipid metabolism. Further studies are required to verify the current findings.

## Data availability statement

The original contributions presented in the study are included in the article/Supplementary material, further inquiries can be directed to the corresponding author.

## Author contributions

XL, ZY, and DZ conceived and designed the research, analyzed the data, interpreted the data, and wrote the first draft. ZY and DZ retrieved the literature and identified eligible studies. XL and DZ extracted the data and checked the statistical methods. XL and ZY reviewed the manuscript and revised the important content. All authors contributed to the article and approved the submitted version.

## Funding

This work was supported by a research grant from Science and Technology Project of Henan Province (no. 222102310176).

## Conflict of interest

The authors declare that the research was conducted in the absence of any commercial or financial relationships that could be construed as a potential conflict of interest.

## Publisher’s note

All claims expressed in this article are solely those of the authors and do not necessarily represent those of their affiliated organizations, or those of the publisher, the editors and the reviewers. Any product that may be evaluated in this article, or claim that may be made by its manufacturer, is not guaranteed or endorsed by the publisher.

## Abbreviations

CVD: Cardiovascular disease
VD: Vitamin D
Ca: Calcium
Mg: Magnesium
WC: Waist circumference
BMI: Body mass index
FBG: Fasting blood glucose
FINS: Fasting insulin level
HOMA-IR: Homeostatic model assessment of insulin resistance
HbA1c: Hemoglobin A1c
SBP: Systolic blood pressure
DBP: Diastolic blood pressure
TGs: Triglycerides
TC: Total cholesterol
HDL-C: High-density lipoprotein cholesterol
LDL-C: Low-density lipoprotein cholesterol
