# Supplementary material for: Nutritional supplements improve cardiovascular risk factors in overweight and obese patients: A Bayesian network meta-analysis
Source: Front Nutr. 2023 Mar 30;10:1140019. doi: 10.3389/fnut.2023.1140019 (PMC10098366; doi:10.3389/fnut.2023.1140019)
Supplement: Supplementary file 1 [file Data_Sheet_1.pdf]

SUPPLEMENTARY APPENDIX TO THE MANUSCRIPT

Nutritional Supplements Improves Cardiovascular Risk Factors in Overweight and Obese Patients: A Bayesian Network Meta-Analysis

Zengli Yu[1], Danyang Zhao[1], Xinxin Liu[1*]

[1*] Department of Nutrition and Hygiene, College of Public Health, Zhengzhou University, No. 100 of Science Road, Zhengzhou, Henan, 450001, China.

Contents of supplementary appendix

Supplementary file 1 1

PRISMA 2020 Checklist 1

Supplementary file 2 4

Search strategies of systematic review 4

Supplementary Table 1 6

Pairwise meta-analysis 6

Blood pressure 6

Glucose and lipid metabolism 7

Body composition 12

Supplementary Table 2 15

Global inconsistency and heterogeneity of each outcome. 15

Supplementary Table 3 16

Node-Splitting results of all outcomes. 16

Supplementary Table 4 17

Results of the network meta-analysis on DBP and SBP. 17

Supplementary Table 5 19

Results of the network meta-analysis on HbA1c and HOMA-IR. 19

Supplementary Table 6 21

Results of the network meta-analysis on HDL-C and LDL-C. 21

Supplementary Table 7 23

Results of the network meta-analysis on Weight and WC. 23

Supplementary Table 8 25

Results of the network meta-analysis on BMI. 25

Supplementary Figure 1 27

Risk of bias graph 27

Risk of bias table 28

Supplementary Figure 2 34

Results of network meta-regression analysis 34

Supplementary Figure 3 47

Detailed results of SCURA ranking 47

Supplementary Figure 4 49

Comparison-adjusted funnel plot 49

Supplementary file 1

PRISMA 2020 Checklist

Section and Topic	Item #	Checklist item	Location where item is reported
**TITLE**			
Title	1	Identify the report as a systematic review.	1
**ABSTRACT**			
Abstract	2	See the PRISMA 2020 for Abstracts checklist.	2
**INTRODUCTION**			
Rationale	3	Describe the rationale for the review in the context of existing knowledge.	3-4
Objectives	4	Provide an explicit statement of the objective(s) or question(s) the review addresses.	4
**METHODS**			
Eligibility criteria	5	Specify the inclusion and exclusion criteria for the review and how studies were grouped for the syntheses.	4-5
Information sources	6	Specify all databases, registers, websites, organisations, reference lists and other sources searched or consulted to identify studies. Specify the date when each source was last searched or consulted.	4
Search strategy	7	Present the full search strategies for all databases, registers and websites, including any filters and limits used.	4 and Supplementary file 2
Selection process	8	Specify the methods used to decide whether a study met the inclusion criteria of the review, including how many reviewers screened each record and each report retrieved, whether they worked independently, and if applicable, details of automation tools used in the process.	4-5
Data collection process	9	Specify the methods used to collect data from reports, including how many reviewers collected data from each report, whether they worked independently, any processes for obtaining or confirming data from study investigators, and if applicable, details of automation tools used in the process.	5
Data items	10a	List and define all outcomes for which data were sought. Specify whether all results that were compatible with each outcome domain in each study were sought (e.g. for all measures, time points, analyses), and if not, the methods used to decide which results to collect.	5
	10b	List and define all other variables for which data were sought (e.g. participant and intervention characteristics, funding sources). Describe any assumptions	5

		made about any missing or unclear information.	
Study risk of bias assessment	11	Specify the methods used to assess risk of bias in the included studies, including details of the tool(s) used, how many reviewers assessed each study and whether they worked independently, and if applicable, details of automation tools used in the process.	6
Effect measures	12	Specify for each outcome the effect measure(s) (e.g. risk ratio, mean difference) used in the synthesis or presentation of results.	6
Synthesis methods	13a	Describe the processes used to decide which studies were eligible for each synthesis (e.g. tabulating the study intervention characteristics and comparing against the Placebonned groups for each synthesis (item #5)).	6
	13b	Describe any methods required to prepare the data for presentation or synthesis, such as handling of missing summary statistics, or data conversions.	6
	13c	Describe any methods used to tabulate or visually disPlaceboy results of individual studies and syntheses.	6
	13d	Describe any methods used to synthesize results and provide a rationale for the choice(s). If meta-analysis was performed, describe the model(s), method(s) to identify the presence and extent of statistical heterogeneity, and software package(s) used.	6-7
	13e	Describe any methods used to explore possible causes of heterogeneity among study results (e.g. subgroup analysis, meta-regression).	7
	13f	Describe any sensitivity analyses conducted to assess robustness of the synthesized results.	-
Reporting bias assessment	14	Describe any methods used to assess risk of bias due to missing results in a synthesis (arising from reporting biases).	6-7
Certainty assessment	15	Describe any methods used to assess certainty (or confidence) in the body of evidence for an outcome.	6-7
**RESULTS**			
Study selection	16a	Describe the results of the search and selection process, from the number of records identified in the search to the number of studies included in the review, ideally using a flow diagram.	7 and Figure 1
	16b	Cite studies that might appear to meet the inclusion criteria, but which were excluded, and exPlaceboin why they were excluded.	7 and Figure 1
Study characteristics	17	Cite each included study and present its characteristics.	7-8 and Table 1
Risk of bias in studies	18	Present assessments of risk of bias for each included study.	8 and Supplementary Figure 1
Results of individual studies	19	For all outcomes, present, for each study: (a) summary statistics for each group (where appropriate) and (b) an effect estimate and its precision (e.g. confidence/credible interval), ideally using structured tables or plots.	8-11, and Supplementary Table1
Results of syntheses	20a	For each synthesis, briefly summarise the characteristics and risk of bias among contributing studies.	9-10
	20b	Present results of all statistical syntheses conducted. If meta-analysis was done, present for each the summary estimate and its precision (e.g.	8-11, Table 2-3

		confidence/credible interval) and measures of statistical heterogeneity. If comparing groups, describe the direction of the effect.	
	20c	Present results of all investigations of possible causes of heterogeneity among study results.	8, and Supplementary Figure 2
	20d	Present results of all sensitivity analyses conducted to assess the robustness of the synthesized results.	-
Reporting biases	21	Present assessments of risk of bias due to missing results (arising from reporting biases) for each synthesis assessed.	10
Certainty of evidence	22	Present assessments of certainty (or confidence) in the body of evidence for each outcome assessed.	-
**DISCUSSION**			
Discussion	23a	Provide a general interpretation of the results in the context of other evidence.	11-13
	23b	Discuss any limitations of the evidence included in the review.	14-15
	23c	Discuss any limitations of the review processes used.	14-15
	23d	Discuss implications of the results for practice, policy, and future research.	14-15
**OTHER INFORMATION**			
registration and protocol	24a	Provide registration information for the review, including register name and registration number, or state that the review was not registered.	4
	24b	Indicate where the review protocol can be accessed, or state that a protocol was not prepared.	4
	24c	Describe and exPlaceboin any amendments to information provided at registration or in the protocol.	-
Support	25	Describe sources of financial or non-financial support for the review, and the role of the funders or sponsors in the review.	16
Competing interests	26	Declare any competing interests of review authors.	16
Availability of data, code and other materials	27	Report which of the following are publicly available and where they can be found: temPlacebote data collection forms; data extracted from included studies; data used for all analyses; analytic code; any other materials used in the review.	16

Supplementary file 2

Search strategies of systematic review

Database	Search strategy	Number of records
PubMed	("glycemic control"[Tiab] OR glucose[MeSH] OR glucose[tiab] OR "fasting plasma glucose"[Tiab] OR FPG[Tiab] OR FBG[Tiab] OR FBS[Tiab] OR insulin[MeSH] OR insulin[Tiab] OR "Glycated Hemoglobin A"[MeSH] OR "Glycated Hemoglobin"[tiab] OR HbA1c[Tiab] OR "homeostatic model of insulin resistance"[Tiab] OR "HOMA-IR"[Tiab] OR "lipid profile*"[Tiab] OR triglycerides[MeSH] OR triglyceride*[Tiab] OR triacylglycerol[tiab] OR cholesterol[MeSH] OR cholesterol[Tiab] OR VLDL[Tiab] OR LDL[Tiab] OR HDL[Tiab]) AND ("metabolic syndrome"[Mesh] OR "metabolic syndrome*"[tiab] OR "metabolic disorders*"[tiab] OR overweight[MeSH] OR overweight[tiab] OR obesity[Mesh] OR obesity[MeSH] OR obese[tiab]) AND (("Resveratrol"[MeSH] OR " Resveratrol*"[Title/Abstract]) OR ("vitamin D"[MeSH] OR " vitamin D*"[Title/Abstract]) OR ("Thioctic Acid "[MeSH] OR "α-lipoic acid"[Title/Abstract] OR ALA[Title/Abstract]) OR ("Probiotics "[MeSH] OR " Probiotic bacteria "[Title/Abstract] OR Probioticss [Title/Abstract]) OR ("omega-3 fatty acid "[MeSH] OR "ω-3polyunsaturated fatty acid Omega-3"[Title/Abstract]) OR ("magnesium"[MeSH] OR "Mg"[Title/Abstract]) OR ("Curcumin"[MeSH]))	833
Embase	('metabolic syndrome':ti,ab OR 'Metabolic Syndrome X':ti,ab OR 'Mets':ti,ab OR 'overweight':ti,ab OR ' obesity':ti,ab OR 'obese':ti,ab) AND ('waist circumference':ti,ab OR 'body mass index':ti,ab OR 'blood pressure':ti,ab OR 'fasting Placebosma glucose':ti,ab OR 'lipid profile':ti,ab OR 'insulin':ti,ab OR 'Glycated Hemoglobin A':ti,ab OR 'triglycerides':ti,ab OR 'triacylglycerol':ti,ab OR 'cholesterol':ti,ab OR 'WC':ti,ab OR 'BMI':ti,ab OR 'BP':ti,ab OR 'SBP':ti,ab OR 'DBP':ti,ab OR 'FPG':ti,ab OR 'HbA1c':ti,ab OR 'HOMA-IR':ti,ab OR 'LDL':ti,ab OR 'HDL':ti,ab) AND ('randomized controlled trial'/exp OR 'randomized':ti,ab OR 'Placebocebo':ti,ab) AND ('supplementation'/exp OR 'intake':ti,ab OR 'use':ti,ab) AND ('resveratrol':ti,ab OR 'Vitamin D':ti,ab OR 'Thioctic Acid'/exp OR 'alpha lipoic acid':ti,ab OR 'ALA':ti,ab OR 'Curcumin'/exp OR 'curcuminoid':ti,ab OR 'Omega-3'/exp OR 'ω-3polyunsaturated fatty acid':ti,ab OR ' omega-3 fatty acid ':ti,ab 'fish oil':ti,ab OR 'Probiotics'/exp OR 'Probiotic bacteria':ti,ab OR 'Magnesium'/exp OR 'Magnesium':ti,ab)	619
Web of Science	(TI= (metabolic syndrome* OR metabolic disorders* OR overweight OR obesity OR obese) OR AB= (metabolic syndrome* OR metabolic disorders* OR overweight OR obesity OR obese)) AND (TI=(waist circumference OR body mass index OR blood pressure OR BMI OR fasting Placebosma glucose OR FPG OR SBP OR DBP OR insulin OR Glycated Hemoglobin A OR HbA1c OR homeostatic model of insulin resistance OR HOMA-IR OR lipid profile* OR triglycerides OR triacylglycerol OR cholesterol OR LDL OR HDL) OR AB=(waist circumference OR body mass index OR blood pressure OR BMI OR fasting Placebosma glucose OR FPG OR SBP OR DBP OR insulin OR Glycated Hemoglobin A OR HbA1c OR homeostatic model of insulin resistance OR HOMA-IR OR lipid profile* OR triglycerides OR triacylglycerol OR cholesterol OR LDL OR HDL)) AND (TS= (randomized controlled trial OR randomized OR Placebocebo)) AND (TI= (supplementation OR intake* OR use) OR AB=(supplementation OR intake* OR use)) AND (TS= (resveratrol) OR (TI= (vitamin D OR cholecalciferol OR VitD) OR AB= (vitamin D OR cholecalciferol OR VitD)) OR (TI= (thioctic acid OR α-lipoic acid OR ALA) OR AB= (thioctic acid OR α-lipoic acid OR ALA)) OR (TI= (Curcumin) OR AB= (Curcumin)) OR (TI= (omega-3 fatty acid OR ω-3polyunsaturated fatty acid OR fish oil) OR AB= (omega-3 fatty acid OR ω-3polyunsaturated fatty acid OR fish oil)) OR (TI= (Probiotics OR Probiotic bacteria) OR AB= (Probiotics OR Probiotic bacteria)) OR (TI= (Magnesium) OR AB= (Magnesium))	1077

Cochrane	((metabolic syndrome):ti,ab,kw OR (metabolic syndrome X):ti,ab,kw OR (Mest):ti,ab,kw OR(overweight):ti,ab,kw OR (obesity):ti,ab,kw) AND ((waist circumference):ti,ab,kw OR (body mass index):ti,ab,kw OR (fasting Placebosma glucose):ti,ab,kw OR (lipid profile):ti,ab,kw OR (insulin):ti,ab,kw OR (Glycated Hemoglobin A):ti,ab,kw OR (triglycerides):ti,ab,kw OR (triacylglycerol):ti,ab,kw OR (cholesterol):ti,ab,kw OR (WC):ti,ab,kw OR (BMI):ti,ab,kw OR (BP):ti,ab,kw OR (SBP):ti,ab,kw OR (DBP):ti,ab,kw OR (FPG):ti,ab,kw OR (HbA1c):ti,ab,kw OR (HOMA-IR):ti,ab,kw OR (LDL):ti,ab,kw OR (HDL):ti,ab,kw) AND ((randomized controlled trial) ti,ab,kw OR (randomized):ti,ab,kw OR (Placebocebo):ti,ab,kw) AND ((supplementation):ti,ab,kw OR (intake):ti,ab,kw OR (use):ti,ab,kw) AND ((resveratrol):ti,ab,kw OR (vitamin D):ti,ab,kw OR (Thioctic Acid):ti,ab,kw OR (alpha lipoic acid):ti,ab,kw OR (ALA):ti,ab,kw OR (curcumin):ti,ab,kw OR (turmeric):ti,ab,kw OR (curcuminoid):ti,ab,kw OR (Omega-3):ti,ab,kw OR (ω-3polyunsaturated fatty acid):ti,ab,kw OR (omega-3 fatty acid):ti,ab,kw OR (n-3 PUFA):ti,ab,kw OR (Probiotic):ti,ab,kw OR (Probiotic bacteria):ti,ab,kw OR (Magnesium):ti,ab,kw)	1334

Supplementary Table 1

Pairwise meta-analysis

Blood pressure

Comparisons	Studies	Participants	Heterogeneity	WMD (95%CI)
**SBP**				
Resveratrol vs Placebo	4	245	(P<0.01), I^2=82	-0.34 (-1.39, 0.70)
VD vs Placebo	6	429	($P=0.51$), I^2=0	-0.11 (-0.30, 0.08)
Probiotics vs Placebo	3	217	(P<0.01), I^2=96	-2.10 (-5.82, 1.61)
**α-lipoic acid vs Placebo**	**1**	**43**	**NA**	***-14.89(-18.24, -11.54)***
omega-3 vs Placebo	6	346	(P<0.01), I^2=96	-0.14 (-1.15, 0.87)
Curcumin vs Placebo	3	143	(P=0.91), I^2=0	-0.04 (-0.37, 0.29)
Mg vs Placebo	4	300	(P=0.79), I^2=0	-0.18, (-0.41, 0.05)
VD＋Ca vs Placebo	1	63	NA	-0.26 (-0.75, 0.24)
**α-lipoic acid ＋ Probiotics vs Placebo**	**1**	**43**	**NA**	***-7.77 (-9.59, -5.95)***
**α-lipoic acid ＋ Probiotics vs α-lipoic acid**	**1**	**44**	**NA**	***-1.65 (-2.34, -0.95)***
**α-lipoic acid ＋ Probiotics vs Probiotics**	**1**	**43**	**NA**	***-0.73 (-1.35, -0.11)***
α-lipoic acid vs Probiotics	1	43	NA	0.54 (-0.07, 1.15)
**DBP**				
Resveratrol vs Placebo	4	245	($P<0.01$), I^2=77	0.01 (-0.75, 0.78)
VD vs Placebo	6	429	($P=0.83$), I^2=0	-0.02 (-0.21, 0.17)
Probiotics vs Placebo	3	217	($P<0.01$), I^2=96	-1.56 (-4.27, 1.14)
**α-lipoic acid vs Placebo**	**1**	**43**	**NA**	***-4.34 (-5.47, -3.21)***
omega-3 vs Placebo	6	346	($P<0.01$), I^2=98	-6.68 (-5.60, 18.95)
Curcumin vs Placebo	3	143	(P=0.02), I^2=75	0.57 (-0.31, 1.44)
Mg vs Placebo	4	300	(P=0.12), I^2=48	-0.41 (-0.75, 0.06)
VD＋Ca vs Placebo	1	103	NA	0.05 (-0.44, 0.55)
**α-lipoic acid ＋ Probiotics vs Placebo**	**1**	**43**	**NA**	***-6.23 (-7.74, -4.73)***
**α-lipoic acid ＋ Probiotics vs α-lipoic acid**	**1**	**44**	**NA**	***-2.50 (-3.30, -1.69)***
α-lipoic acid＋Probiotics vs Probiotics	1	43	NA	-1.40 (-2.08, 0.73)
**α-lipoic acid vs Probiotics**	**1**	**43**	**NA**	***0.77 (0.14, 1.39)***

Glucose and lipid metabolism

Comparisons	Studies	Participants	Heterogeneity	WMD (95%CI)
**FGB**				
Resveratrol vs Placebo	5	297	(P<0.01), I^2=89	-0.38 (-1.06, 0.30)
VD vs Placebo	9	518	(P=0.01), I^2=58	-0.26 (-0.55, 0.03)
Probiotics vs Placebo	9	452	(P<0.01), I^2=97	-2.32 (-4.88, 0.23)
α-lipoic acid vs Placebo	1	37	NA	0.47 (-0.19, 1.13)
omega-3 vs Placebo	14	649	(P<0.01), I^2=92	-0.25 (-0.68, 0.18)
Curcumin vs Placebo	7	335	(P=0.51), I^2=0	-014 (-0.35, 0.08)
Mg vs Placebo	6	384	($P<0.01$), I^2=81	-0.33 (-0.91, 0.26)
VD＋Ca vs Placebo	1	63	NA	-0.05 (-0.55, 0.44)
omega-3 ＋ α-lipoic acid vs Placebo	1	38	NA	-0.04 (-0.68, 0.60)
omega-3 ＋ α-lipoic acid vs omega-3	1	36	NA	-0.47 (-1.13, 0.20)
omega-3＋α-lipoic acid vs α-lipoic acid	1	33	NA	-0.46 (-1.15, 0.24)
omega-3 vs α-lipoic acid	1	35	NA	0.00 (-0.67, 0.67)
**Probiotics ＋ omega-3 vs Placebo**	**1**	**60**	**NA**	***-5.55 (-6.69, -4.40)***
**Probiotics ＋ omega-3 vs Probiotics**	**1**	**60**	**NA**	***-2.37 (-3.04, -1.70)***
**Probiotics ＋ omega-3 vs omega-3**	**1**	**60**	**NA**	***-5.23 (-6.32, -4.14)***
**omega-3 vs Probiotics**	**1**	**60**	**NA**	***4.12 (3.21, 5.03)***
**FINS**				
Resveratrol vs Placebo	5	208	(P=0.75), I^2=0	-0.03 (-0.31, 0.25)
VD vs Placebo	5	202	(P<0.01), I^2=87	0.11 (-0.82, 1.04)
Probiotics vs Placebo	7	398	(P<0.01), I^2=91	0.71 (-1.51, 0.09)
omega-3 vs Placebo	10	497	(P<0.01), I^2=84	-0.32 (-0.84, 0.20)
Curcumin vs Placebo	5	204	(P<0.01), I^2=81	0.01 (-0.99, 1.02)
Mg vs Placebo	6	384	($P=0.05$), I^2=54	-0.16 (-0.49, 0.16)
VD＋Ca vs Placebo	1	63	NA	-0.06 (-0.56, 0.43)
VD＋Ca vs VD	1	41	NA	0.58 (-0.05, 1.21)
Probiotics＋VD vs Placebo	1	61	NA	-0.12(-0.63, 0.38)
Probiotics vs VD	1	58	NA	0.26(-0.26, 0.77)
Probiotics＋VD vs Probiotics	1	58	NA	-0.28(-0.8, 0.24)
Probiotics＋VD vs VD	1	60	NA	0.08(-0.43, 0.59)
**α-lipoic acid＋Probiotics vs Placebo**	**1**	**43**	**NA**	***-2.51(-3.33, -1.69)***

**α-lipoic acid＋Probiotics vs α-lipoic acid**	**1**	**44**	**NA**	***-1.79(-2.5, -1.08)***
α-lipoic acid＋Probiotics vs Probiotics	1	43	NA	-0.26(-0.86, 0.34)
**α-lipoic acid vs Probiotics**	**1**	**43**	**NA**	***2.17 (1.4, 2.93)***
omega-3 ＋ α-lipoic acid vs Placebo	1	34	NA	-0.25 (-0.93, 0.43)
omega-3 ＋ α-lipoic acid vs omega-3	1	30	NA	-0.36 (-1.09, 0.36)
omega-3＋α-lipoic acid vs α-lipoic acid	1	31	NA	0.12 (-0.58, 0.83)
omega-3＋vs α-lipoic acid	1	31	NA	0.49 (-0.23, 1.2)
**Probiotics ＋ omega-3 vs Placebo**	**1**	**60**	**NA**	***-4.04 (-4.94, -3.14)***
**Probiotics ＋ omega-3 vs Probiotics**	**1**	**60**	**NA**	***-1.57 (-2.15, -0.99)***
**Probiotics ＋ omega-3 vs omega-3**	**1**	**60**	**NA**	***-2.30 (-2.96, -1.64)***
omega-3 vs Probiotics	1	60	NA	0.59 (-1.15, 0.11)
**HOMA-IR**				
Resveratrol vs Placebo	4	208	(*P*<0.01), I^2=75	-0.22 (-0.96, 0.52)
VD vs Placebo	5	252	(*P*<0.01), I^2=82	-0.26 (-0.91, 0.39)
Probiotics vs Placebo	5	279	(*P*<0.01), I^2=93	-1.02 (-2.61, 0.57)
α-lipoic acid vs Placebo	1	35	NA	0.22 (-0.45, 0.89)
omega-3 vs Placebo	4	159	(*P*=0.02), I^2=69	0.19 (-0.42, 0.81)
**Curcumin vs Placebo**	**3**	**143**	***(P*=0.60*), I^2=0***	***-0.41 (-0.74, -0.08)***
Mg vs Placebo	5	370	(*P*<0.01), I^2=96	-1.24 (-2.82, 0.35)
VD＋Ca vs Placebo	2	103	*(P*=0.10*), I^2=63*	-0.08 (-0.74, 0.58)
**VD＋Ca vs VD**	**1**	**41**	**NA**	***0.84 (0.2, 1.49)***
Probiotics＋VD vs Placebo	1	61	NA	-0.10 (-0.60, 0.4)
Probiotics vs VD	1	58	NA	0.48 (-0.04, 1.00)
**Probiotics＋VD vs Probiotics**	**1**	**58**	**NA**	***-0.66 (-1.19, -013)***
Probiotics＋VD vs VD	1	60	NA	-0.18 (-0.69, 0.33)
**α-lipoic acid＋Probiotics vs Placebo**	**1**	**43**	**NA**	***-2.59(-3.42, -1.76)***
**α-lipoic acid＋Probiotics vs α-lipoic acid**	**1**	**44**	**NA**	***-0.84 (-1.46, -0.22)***
**α-lipoic acid＋Probiotics vs Probiotics**	**1**	**43**	**NA**	***-1.25 (-1.91, -0.59)***
**α-lipoic acid vs Probiotics**	**1**	**43**	**NA**	***-1.09 (-1.73, -0.44)***
omega-3 ＋ α-lipoic acid vs Placebo	1	34	NA	-0.14 (-0.82, 0.54)

**omega-3 ＋ α-lipoic acid vs omega-3**	**1**	**30**	**NA**	***-0.79 (-1.52, -0.04)***
omega-3＋α-lipoic acid vs α-lipoic acid	1	31	NA	-0.55 (-1.27, 0.17)
omega-3 vs α-lipoic acid	1	31	NA	0.34 (-0.37, 1.05)
**HbA1c**				
Resveratrol vs Placebo	3	162	(P=0.11), I^2=55	0.30 (-0.26, 0.85)
VD vs Placebo	3	239	(P<0.01), I^2=90	0.35 (-0.63, 1.34)
Probiotics vs Placebo	1	116	NA	-0.12 (-0.48, 0.25)
omega-3 vs Placebo	1	50	NA	-0.07 (-0.62, 0.49)
**Curcumin vs Placebo**	**3**	**143**	***(P=0.30), I^2=16***	***-0.36 (-0.70, -0.01)***
Mg vs Placebo	1	14	NA	0.37 (-0.69, 1.43)
**TGs**				
Resveratrol vs Placebo	6	313	(P=0.04), I^2=56	-0.12 (-0.52, 0.28)
VD vs Placebo	9	598	(P=0.72), I^2=0	-0.13 (-0.29, 0.03)
Probiotics vs Placebo	9	527	*(P=0.10), I^2=49*	-0.21 (-0.39, -0.04)
α-lipoic acid vs Placebo	1	35	NA	-0.35 (-1.02, 0.33)
**omega-3 vs Placebo**	**12**	**589**	***(P=0.89), I^2=0***	***-0.29 (-0.46, -0.13)***
Curcumin vs Placebo	6	304	(P<0.01), I^2=68	-0.16 (-0.57, 0.24)
Mg vs Placebo	5	370	(P<0.01), I^2=94	-0.62 (-1.72, 0.48)
VD＋Ca vs Placebo	2	103	(P=0.39), I^2=0	-0.25 (-0.64, 0.14)
VD＋Ca vs VD	1	41	NA	-0.03 (-0.64, 0.59)
Probiotics＋VD vs Placebo	1	61	NA	-0.36 (-0.87, 0.14)
**Probiotics vs VD**	**1**	**58**	**NA**	***-0.57 (-1.10, -0.05)***
Probiotics＋VD vs Probiotics	1	58	NA	0.43 (-0.09, 0.95)
Probiotics＋VD vs VD	1	60	NA	-0.19 (-0.70, 0.32)
omega-3 ＋ α-lipoic acid vs Placebo	1	34	NA	-0.26 (-0.94, 0.42)
omega-3 ＋ α-lipoic acid vs omega-3	1	30	NA	-0.40 (-1.13, 0.32)
omega-3＋α-lipoic acid vs α-lipoic acid	1	31	NA	0.10 (-0.60, 0.81)
omega-3 vs α-lipoic acid	1	31	NA	0.48 (−0.24, 1.20)
**Probiotics ＋ omega-3 vs Placebo**	**1**	**60**	**NA**	***-0.60 (-1.12, -0.09)***
Probiotics ＋ omega-3 vs Probiotics	1	60	NA	-0.27 (-0.77, 0.24)
**Probiotics ＋ omega-3 vs omega-3**	**1**	**60**	**NA**	***-1.05 (-1.59, -0.51)***
omega-3 vs Probiotics	1	60	NA	0.32 (-0.19, 0.83)
**TC**				
Resveratrol vs Placebo	4	252	(P=0.08), I^2=56	0.09 (-0.34, 0.52)

VD vs Placebo	9	483	(*P*<0.01), I^2=89	-0.47 (-1.39, 0.44)
**Probiotics vs Placebo**	**9**	**527**	***(P=0.24), I^2=23***	***-0.36 (-0.57, -0.15)***
omega-3 vs Placebo	10	427	(*P*=0.16), I^2=31	-0.09 (-0.30, 0.12)
Curcumin vs Placebo	6	304	(*P*=0.34), I^2=12	0.16 (-0.11, 0.42)
Mg vs Placebo	4	323	(*P*<0.01), I^2=93	-0.53 (-1.60, 0.54)
VD＋Ca vs Placebo	2	103	(*P*=0.44), I^2=0	-0.21 (-0.60, 0.17)
VD＋Ca vs VD	1	41	NA	-0.16 (-0.77, 0.45)
Probiotics＋VD vs Placebo	1	61	NA	-0.11 (-0.61, 0.39)
Probiotics vs VD	1	58	NA	-0.29 (-0.81, 0.22)
Probiotics＋VD vs Probiotics	1	58	NA	0.39 (-0.81, 0.22)
Probiotics＋VD vs VD	1	60	NA	0.11 (-0.40, 0.62)
**α-lipoic acid＋Probiotics vs Placebo**	**1**	**43**	**NA**	***-2.51(-3.33, -1.69)***
**α-lipoic acid＋Probiotics vs α-lipoic acid**	**1**	**44**	**NA**	***-1.79(-2.5, -1.08)***
α-lipoic acid＋Probiotics vs Probiotics	1	43	NA	-0.26(-0.86, 0.34)
**α-lipoic acid vs Probiotics**	**1**	**43**	**NA**	***2.17 (1.4, 2.93)***
omega-3 ＋ α-lipoic acid vs Placebo	1	34	NA	-0.25 (-0.93, 0.43)
omega-3 ＋ α-lipoic acid vs omega-3	1	30	NA	-0.36 (-1.09, 0.36)
omega-3＋α-lipoic acid vs α-lipoic acid	1	31	NA	0.12 (-0.58, 0.83)
omega-3＋vs α-lipoic acid	1	31	NA	0.49 (-0.23, 1.2)
**Probiotics ＋ omega-3 vs Placebo**	**1**	**60**	**NA**	***-0.94 (-1.48, -0.41)***
Probiotics ＋ omega-3 vs Probiotics	1	60	NA	-0.23 (-0.74, 0.23)
**Probiotics ＋ omega-3 vs omega-3**	**1**	**60**	**NA**	***-0.69 (-1.22, -0.17)***
**omega-3 vs Probiotics**	**1**	**60**	**NA**	***0.57 (0.05, 1.08)***
**HDL-C**				
Resveratrol vs Placebo	4	252	(*P*<0.01), I^2=77	-0.31 (-1.04, 0.42)
VD vs Placebo	10	648	(*P*=0.24), I^2=22	0.14 (-0.02, 0.29)
**Probiotics vs Placebo**	**9**	**527**	***(P<0.01), I^2=91***	***-0.21 (-0.52, 0.93)***
α-lipoic acid vs Placebo	1	35	NA	-0.34 (-1.01, 0.33)
omega-3 vs Placebo	12	589	(*P*<0.01), I^2=63	0.05 (-0.24, 0.33)
**Curcumin vs Placebo**	**6**	**304**	***(P=0.70), I^2=0***	***0.35 (0.12, 0.57)***
Mg vs Placebo	5	370	(*P*<0.01), I^2=95	0.86 (-0.57, 2.29)
VD＋Ca vs Placebo	2	103	(*P*=0.19), I^2=43	0.09 (-0.43, 0.62)
VD＋Ca vs VD	1	41	NA	-0.40 (-1.02, 0.22)

Probiotics＋VD vs Placebo	1	61	NA	-0.26 (-0.77, 0.24)
Probiotics vs VD	1	58	NA	-0.52 (-1.04, 0.94)
Probiotics＋VD vs Probiotics	1	58	NA	0.41 (-0.11, 0.94)
Probiotics＋VD vs VD	1	60	NA	-0.08 (-0.59, 0.42)
**α-lipoic acid＋Probiotics vs Placebo**	**1**	**43**	**NA**	***-2.59(-3.42, -1.76)***
**α-lipoic acid＋Probiotics vs α-lipoic acid**	**1**	**44**	**NA**	***-0.84 (-1.46, -0.22)***
**α-lipoic acid＋Probiotics vs Probiotics**	**1**	**43**	**NA**	***-1.25 (-1.91, -0.59)***
**α-lipoic acid vs Probiotics**	**1**	**43**	**NA**	***-1.09 (-1.73, -0.44)***
omega-3 ＋ α-lipoic acid vs Placebo	1	34	NA	-0.09 (-0.76, 0.59)
omega-3 ＋ α-lipoic acid vs omega-3	1	30	NA	0.12 (-0.59, 0.84)
omega-3＋α-lipoic acid vs α-lipoic acid	1	31	NA	0.22 (-0.48, 0.93)
omega-3＋vs α-lipoic acid	1	31	NA	0.10 (-0.60, 0.81)
**Probiotics ＋ omega-3 vs Placebo**	**1**	**60**	**NA**	***3.07 (2.31, 3.83)***
**Probiotics ＋ omega-3 vs Probiotics**	**1**	**60**	**NA**	***1.19 (0.63, 1.74)***
**Probiotics ＋ omega-3 vs omega-3**	**1**	**60**	**NA**	***2.57 (1.87, 3.26)***
**omega-3 vs Probiotics**	**1**	**60**	**NA**	***-2.03 (-2.66, -1.40)***
**LDL-C**				
Resveratrol vs Placebo	4	252	(*P*<0.01), I^2=88	0.64 (-0.22, 1.51)
VD vs Placebo	10	648	(*P*=0.17), I^2=30	0.01 (-0.19, 0.21)
**Probiotics vs Placebo**	**9**	**527**	***(P=0.22), I^2=25***	***-0.32 (-0.52, -0.12)***
α-lipoic acid vs Placebo	1	35	NA	-0.32 (-0.99, 0.35)
omega-3 vs Placebo	12	589	(*P*<0.01), I^2=91	0.20 (-0.31, 0.71)
Curcumin vs Placebo	6	304	(*P*<0.01), I^2=72	-0.05 (-0.49, 0.39)
Mg vs Placebo	4	323	(*P*<0.01), I^2=95	-0.75 (-2.18, 0.69)
VD＋Ca vs Placebo	2	41	(*P*=0.64), I^2=0	-0.26 (-0.64, 0.13)
VD＋Ca vs VD	1	61	NA	-0.26 (-0.88, 0.35)
Probiotics＋VD vs Placebo	1	61	NA	-0.32 (-0.83, 0.18)
Probiotics vs VD	1	58	NA	-0.18 (-0.75, 0.39)
Probiotics＋VD vs Probiotics	1	58	NA	0.00 (-0.51, 0.52)
Probiotics＋VD vs VD	1	60	NA	-0.17 (-0.73, 0.39)
omega-3 ＋ α-lipoic acid vs Placebo	1	34	NA	-0.29 (-0.97, 0.39)
omega-3 ＋ α-lipoic acid vs	1	30	NA	0.04 (-0.67, 0.76)

omega-3				
omega-3＋α-lipoic acid vs α-lipoic acid	1	31	NA	-0.02 (-0.72, 0.68)
omega-3＋vs α-lipoic acid	1	31	NA	-0.07 (-0.79, 0.63)
**Probiotics ＋ omega-3 vs Placebo**	**1**	**60**	**NA**	***-1.37 (-1.94, -0.80)***
**Probiotics ＋ omega-3 vs Probiotics**	**1**	**60**	**NA**	***-1.10 (-1.65, -0.56)***
**Probiotics ＋ omega-3 vs omega-3**	**1**	**60**	**NA**	***-1.02 (-1.56, -0.48)***
omega-3 vs Probiotics	1	60	NA	0.26 (-0.25, 0.77)

Body composition

Comparisons	Studies	Participants	Heterogeneity	WMD (95%CI)
**Weight**				
Resveratrol vs Placebo	2	61	(P=0.91), I^2=0	-0.07 (-0.59, 0.44)
VD vs Placebo	16	1434	(P<0.01), I^2=82	-0.19 (-0.49, 0.10)
Probiotics vs Placebo	9	487	(P=0.94), I^2=0	-0.17 (-0.35, 0.01)
α-lipoic acid vs Placebo	2	80	(P=0.86), I^2=0	-0.17 (-0.61, 0.27)
omega-3 vs Placebo	10	483	(P<0.01), I^2=91	-0.03 (-0.65, 0.59)
Curcumin vs Placebo	5	193	(P=0.91), I^2=0	-0.04 (-0.32, 0.24)
VD＋Ca vs Placebo	2	103	(P=0.81), I^2=0	-0.06 (-0.45, 0.33)
VD＋Ca vs VD	1	41	NA	0.05 (-0.56, 0.66)
Probiotics＋VD vs Placebo	1	61	NA	-0.13 (-0.64, 0.37)
Probiotics vs VD	1	58	NA	-0.08 (-0.60, 0.43)
Probiotics＋VD vs Probiotics	1	58	NA	0.06 (-0.46, 0.57)
Probiotics＋VD vs VD	1	60	NA	-0.04 (-0.55, 0.47)
α-lipoic acid ＋ Probiotics vs Placebo	1	43	NA	-0.40 (-1.00, 0.21)
α-lipoic acid＋Probiotics vs α-lipoic acid	1	44	NA	-0.23 (-0.83, 0.36)
α-lipoic acid ＋ Probiotics vs Probiotics	1	43	NA	-0.26(-0.86, 0.34)
α-lipoic acid vs Probiotics	1	43	NA	0.07 (-0.53, 0.66)
omega-3 ＋ α-lipoic acid vs Placebo	1	38	NA	-0.09 (-0.73, 0.55)
omega-3 ＋ α-lipoic acid vs omega-3	1	36	NA	-0.10 (-0.75, 0.56)
omega-3＋α-lipoic acid vs α-lipoic acid	1	35	NA	0.14 (-0.52, 0.81)

omega-3＋vs α-lipoic acid	1	31	NA	0.49 (-0.23, 1.2)
**WC**				
Resveratrol vs Placebo	2	61	(*P*=0.47), I^2=0	-0.10 (-0.61, 0.42)
VD vs Placebocebo	11	822	(*P*<0.01), I^2=72	-0.06 (-0.33, 0.22)
Probiotics vs Placebo	6	371	(*P*<0.01), I^2=82	-0.43 (-1.09, 0.22)
α-lipoic acid vs Placebo	2	78	(*P*=0.65), I^2=0	-0.13 (-0.58, 0.31)
omega-3 vs Placebo	10	501	(*P*=0.98), I^2=0	-0.08 (-0.26, 0.10)
Curcumin vs Placebo	4	173	(*P*=0.44), I^2=0	-0.20 (-0.50, 0.10)
Mg vs Placebo	1	47	NA	-0.03 (-0.60, 0.54)
VD＋Ca vs Placebo	2	103	(*P*=0.72), I^2=0	0.06 (-0.33, 0.04)
VD＋Ca vs VD	1	41	NA	0.13 (-0.48,0.75)
Probiotics＋VD vs Placebo	1	61	NA	0.27 (-0.23, 0.77)
Probiotics vs VD	1	58	NA	-0.23(-0.74, 0.29)
Probiotics＋VD vs Probiotics	1	58	NA	0.40 (-0.12, 0.92)
Probiotics＋VD vs VD	1	60	NA	0.05 (-0.46, 0.55)
**α-lipoic acid＋Probiotics vs Placebo**	**1**	**43**	**NA**	***-2.49 (-3.31, -1.68)***
**α-lipoic acid＋Probiotics vs α-lipoic acid**	**1**	**44**	**NA**	***-2.45 (-3.25, -1.65)***
α-lipoic acid＋Probiotics vs Probiotics	1	43	NA	-0.60 (-1.22, 0.01)
**α-lipoic acid vs Probiotics**	**1**	**43**	**NA**	***2.26 (1.48, 3.04)***
omega-3 ＋ α-lipoic acid vs Placebo	1	34	NA	-0.08 (-0.75, 0.60)
omega-3 ＋ α-lipoic acid vs omega-3	1	30	NA	0.02 (-0.69, 0.74)
omega-3＋α-lipoic acid vs α-lipoic acid	1	31	NA	-0.09 (-0.79, 0.62)
omega-3 vs α-lipoic acid	1	31	NA	-0.11 (-0.82, 0.59)
**BMI**				
Resveratrol vs Placebo	4	179	(*P*=0.06), I^2=59	-0.29 (-0.82, 0.23)
VD vs Placebo	16	1434	(*P*<0.01), I^2=91	-0.44 (-1.01, 0.14)
Probiotics vs Placebo	10	515	(*P*=0.76), I^2=0	-0.15 (-0.32, 0.03)
α-lipoic acid vs Placebo	3	115	(*P*=0.99), I^2=0	-0.12 (-0.49, 0.25)
omega-3 vs Placebo	13	623	(*P*<0.01), I^2=79	0.35 (-0.38, 1.09)
Curcumin vs Placebo	6	273	(*P*=0.94), I^2=0	-0.09 (-0.33, 0.15)
Mg vs Placebo	3	164	(*P*=1), I^2=0	0.00 (-0.30, 0.31)
VD＋Ca vs Placebo	2	103	(*P*=0.76), I^2=0	-0.08 (-0.46, 0.31)
VD＋Ca vs VD	1	41	NA	0.03 (-0.58, 0.64)
Probiotics＋VD vs Placebo	1	61	NA	0.10 (-0.40, 0.60)
Probiotics vs VD	1	58	NA	0.14 (-0.38, 0.65)
Probiotics＋VD vs Probiotics	1	58	NA	0.01 (-0.50, 0.53)

Probiotics＋VD vs VD	1	60	NA	0.18 (-0.32, 0.69)
**α-lipoic acid＋Probiotics vs Placebo**	**1**	**43**	**NA**	***-1.22 (-1.88, -0.56)***
**α-lipoic acid＋Probiotics vs α-lipoic acid**	**1**	**44**	**NA**	***-1.09 (-1.72, -0.45)***
**α-lipoic acid＋Probiotics vs Probiotics**	**1**	**43**	**NA**	***-0.75 (-1.35, -0.11)***
α-lipoic acid vs Probiotics	1	43	NA	0.03 (-0.31, 0.90)
omega-3 ＋ α-lipoic acid vs Placebo	2	72	(P<0.01), I^2=98	-4.58 (-13.51, 4.35)
omega-3 ＋ α-lipoic acid vs omega-3	2	66	(P=0.82), I^2=0	-0.14 (-0.63, 0.34)
omega-3＋α-lipoic acid vs α-lipoic acid	2	64	(P=0.92), I^2=0	0.05 (-0.44, 0.54)
omega-3 vs α-lipoic acid	2	66	(P<0.01), I^2=98	-0.13 (-1.25, 0.98)

Supplementary Table 2

Global inconsistency and heterogeneity of each outcome.

Outcomes	Consistency model					Inconsistency model					dDIC
	Dbar	data points	pD	DIC	I^2	Dbar	data points	pD	DIC	I^2	
SBP	58.81	56	54.92	113.73	6%	58.78	56	54.90	113.68	0%	0.05
DBP	55.39	56	55.21	110.60	0.7%	55.40	56	55.23	110.63	0.7%	0.03
FBG	101.95	104	100.96	202.91	0%	101.95	104	100.97	202.91	0%	0
FINS	102.90	78	65.39	168.29	24%	102.91	78	65.34	168.5	24%	0.21
HOMA-IR	54.51	54	49.98	104.49	3%	54.48	54	50.00	104.48	3%	0.01
Hb1Ac	22.50	24	21.90	44.41	0%	22.55	24	21.94	44.49	0%	0.08
TGs	110.51	97	70.61	181.12	13%	110.73	97	70.61	181.34	13%	0.22
TC	85.81	85	80.75	166.56	2%	85.81	85	80.73	166.54	2%	0.02
HDL-C	100.71	97	75.73	176.44	5%	110.67	97	75.76	176.43	5%	0.01
LDL-C	95.04	93	73.59	168.63	3%	95.13	93	73.64	168.77	3%	0.14
Weight	70.27	91	60.17	130.43	0%	70.21	91	60.11	130.32	0%	0.11
WC	76.88	75	58.86	135.74	4%	77.03	75	58.83	135.86	4%	0.12
BMI	114.36	113	111.08	225.44	2%	114.41	113	111.12	225.54	2%	0.1

DIC: deviance information criterion; dDIC: the difference between each pair of DICs.

Supplementary Table 3

Node-Splitting results of all outcomes.

Study	Mean Difference (95%Crl)			P-value
	Direct	Indirect	Network	
**FBG**				
Omega-3 vs Probiotics	0.28 (-1.26, 1.81)	0.96 (0.25, 1.68)	0.84 (0.20, 1.47)	0.417
**FINS**				
Omega-3 vs Probiotics	0.44 (-4.47, 5.36)	2.17 (-1.15, 5.55)	1.52 (-0.95, 4.17)	0.534
**TGs**				
Probiotics vs VD	-43.26 (-88.69, 0.30)	-0.70 (-16.42, 16.84)	-4.89 (-20.74, 11.08)	0.070
VD＋Ca vs VD	-1.16 (-38.32, 36.12)	-15.01 (-52.05, 23.31)	-6.41 (-31.28, 19.16)	0.594
Omega-3 vs Probiotics	4.26 (-22.71, 31.29)	-0.44 (-18.89, 16.72)	1.78 (-12.58, 14.04)	0.751
**TC**				
Probiotics vs VD	-3.31 (-55.83, 49.12)	13.38 (-13.99, 40.86)	9.71 (-13.82, 33.36)	0.568
VD＋Ca vs VD	-5.43 (-61.32, 50.29)	10.9 (-45.91, 68.11)	7.01 (-30.41, 44.56)	0.674
Omega-3 vs Probiotics	4.52 (-48.32, 57.78)	8.61 (-19.19,36.33)	7.63 (-15.83, 31.55)	0.890
**HDL-C**				
Probiotics vs VD	-27.91 (-55.93, 0.07)	-0.70 (-3.91, 2.35)	-1.01 (-4.24, 2.07)	0.058
VD＋Ca vs VD	-2.99 (-9.73, 3.74)	1.59 (-6.12, 9.25)	-1.31 (-6.19, 3.57)	0.376
Omega-3 vs Probiotics	-2.87 (-7.46, 1.70)	-0.35 (-3.35, 2.84)	-1.11 (-3.79, 1.79)	0.337
**LDL-C**				
Probiotics vs VD	-10.35(-41.11,20.81)	-5.69 (-17.74, 6.79)	-6.34 (-17.35, 4.98)	0.778
VD＋Ca vs VD	-6.03 (-30.97, 18.86)	-8.04 (-33.78, 17.98)	-6.25 (-22.77, 10.56)	0.907
Omega-3 vs Probiotics	2.06 (-18.84, 23.06)	10.49 (-1.84, 22.98)	8.22 (-2.02, 18.73)	0.476
**Weight**				
Probiotics vs VD	-1.20 (-9.30, 6.80)	-0.90 (-3.10, 1.30)	-0.93 (-3.10,1.20)	0.938
VD＋Ca vs VD	0.47 (-5.90, 6.90)	-0.13 (-5.80, 5.50)	0.22 (-4.00, 4.30)	0.891
α-lipoic acid vs Probiotics	0.47 (-4.60, 5.50)	0.18 (-7.30, 7.60)	0.30 (-3.60, 4.10)	0.950
Omega-3 vs α-lipoic acid	1.80 (-6.30, 9.70)	1.60 (-3.20, 6.40)	1.70 (-2.30, 5.70)	0.974
**WC**				
Probiotics vs VD	-2.73 (-10.67, 5.33)	-0.75 (-4.26, 3.02)	-1.03 (-4.08, 2.20)	0.644
α-lipoic acid vs Probiotics	2.69 (-2.71, 8.11)	1.62, (-6.42, 9.66)	2.15 (-1.91, 5.99)	0.812
Omega-3 vs α-lipoic acid	-1.02 (-8.98, 6.86)	-0.45 (-5.87, 4.88)	-0.52 (-4.72, 3.52)	0.901
**BMI**				
Probiotics vs VD	0.49 (-10.00, 11.00)	-0.48 (-4.91, 3.95)	-0.33 (-4.31, 3.63)	0.864
VD＋Ca vs VD	0.10 (-10.88, 10.81)	-0.04(-10.88, 10.81)	0.10 (-6.89, 7.09)	0.984
α-lipoic acid vs Probiotics	0.19 (-9.95, 10.32)	-7.61 (-15.03, -0.14)	-4.49 (-10.36, 1.42)	0.219
Omega-3 vs α-lipoic acid	16.92 (12.15, 21.76)	0.01 (-5.98, 6.01)	7.76 (2.23, 13.32)	0.974

P-value < 0.05 may show significant inconsistency.

Supplementary Table 4

Results of the network meta-analysis on DBP and SBP.

	SBP												
**DBP**	**Placebo**	-0.94 (-13.44, 11.67)	-2.09 (-12.52, 8.30)	-7.67 (-22.09, 6.81)	—	-2.52 (-27.60, 22.56)	-10.65 (-33.09, 12.12)	-9.05 (-19.87, 0.97)	—	-14.68 (-37.31, 7.85)	-0.74 (-15.71, 14.11)	—	-3.24 (-16.15, 9.62)
	-0.60 (-11.11, 9.88)	**Resveratrol**	-1.15 (-17.54, 15.14)	-6.74 (-25.83, 12.38)	—	-1.60 (-29.82, 26.39)	-9.68 (-35.51, 16.25)	-8.10 (-24.86, 7.69)	—	-13.72 (-39.83, 12)	0.20 (-19.31, 19.65)	—	-2.31 (-20.42, 15.67)
	-0.18 (-8.87, 8.56)	0.43 (-13.21, 14.03)	**VD**	-5.59 (-23.36, 12.29)	—	-0.41 (-27.67, 26.63)	-8.55 (-33.43, 16.40)	-6.95 (-22.09, 7.29)	—	-12.59 (-37.55, 12.30)	1.33 (-16.84, 19.53)	—	-1.15 (-17.73, 15.36)
	-2.97 (-15.04, 9.18)	-2.39 (-18.35, 13.78)	-2.80 (-17.74, 12.12)	**Probiotics**	—	5.20 (-23.93, 33.99)	-2.94 (-25.49, 19.56)	-1.36 (-19.63, 16.03)	—	-7.02 (-29.57, 15.62)	6.94 (-13.79, 27.63)	—	4.46 (-14.89, 23.70)
	—	—	—	—	**Probiotics ＋VD**	—	—	—	—	—	—	—	—
	0.44 (-20.69, 21.50)	0.99 (-22.65, 24.65)	0.61 (-22.27, 23.44)	3.40 (-20.96, 27.72)	—	**VD＋Ca**	-8.14 (-41.58, 25.72)	-6.51 (-34.16, 20.24)	—	-12.18 (-45.8, 21.66)	1.74 (-27.41, 30.94)	—	-0.71 (-28.84, 27.60)
	-3.15 (-22.06, 15.80)	-2.55 (-24.13, 19.06)	-2.96 (-23.77, 17.82)	-0.18 (-19.15, 18.84)	—	-3.56 (-31.89, 24.94)	**α-lipoic acid**	1.59 (-23.81, 26.04)	—	-4.07 (-28.91, 20.56)	9.84 (-17.25, 36.93)	—	7.37 (-18.80, 33.25)

	8.06 (-0.58, 16.64)	8.67 (-5.00, 22.21)	8.23 (-4.11, 20.44)	11.01 (-3.85, 25.81)	—	7.60 (-15.16, 30.42)	11.21 (-9.73, 31.96)	**omega-3**	—	-5.63 (-30.24, 19.65)	8.32 (-9.45, 26.80)	—	5.81 (-10.40, 22.81)
	—	—	—	—	—	—	—	—	**omega-3＋α-lipoic acid**	—	—	—	—
	-5.23 (-24.28, 13.73)	-4.66 (-26.47, 17.05)	-5.06 (-26.07, 15.83)	-2.28 (-21.37, 16.74)	—	-5.68 (-34.15, 22.72)	-2.13 (-22.86, 18.68)	-13.31 (-34.14, 7.70)	—	**Probiotics＋α-lipoic acid**	13.93 (-13.11, 41.12)	—	11.44 (-14.45, 37.42)
	1.90 (-10.44, 14.21)	2.52 (-13.78, 18.68)	2.10 (-12.99, 17.15)	4.85 (-12.43, 22.10)	—	1.47 (-22.95, 25.95)	5.02 (-17.57, 27.54)	-6.16 (-21.20, 8.90)	—	7.14 (-15.48, 29.71)	**Curcumin**	—	-2.49 (-22.15, 17.16)
	—	—	—	—	—	—	—	—	—	—	—	**Probiotics＋omega-3**	—
	-3.91 (-14.56, 6.70)	-3.31 (-18.29, 11.59)	-3.73 (-17.52, 9.93)	-0.92 (-17.16, 15.15)	—	-4.37 (-27.96, 19.27)	-0.78 (-22.45, 20.85)	-11.96 (-25.74, 1.70)	—	1.31 (-20.37, 23.01)	-5.84 (-22.09, 10.49)	—	**Mg**

Data are reported as mean difference (95% credible intervals) and indicate column-to-row differences. Statistically significant differences are in bold and inclined formats (P-values <0.05).

SBP: systolic blood pressure; DBP: diastolic blood pressure; VD: Vitamin D; Ca: Calcium; Mg: Magnesium..

Supplementary Table 5

Results of the network meta-analysis on HbA1c and HOMA-IR.

HOMA-IR													
HbA1c	**Placebo**	-0.01 (-1.07, 1.04)	-0.31 (-1.32, 0.69)	**-1.43 (-2.46, -0.31)**	—	—	0.24 (-1.75, 2.23)	0.49 (-0.79, 1.77)	-0.26 (-2.30, 1.76)	—	-0.63 (-1.91, 0.68)	—	-0.43 (-1.40, 0.55)
	0.04 (-0.49, 0.55)	**Resveratrol**	-0.30 (-1.75, 1.15)	-1.41 (-2.88, 0.14)	—	—	0.26 (-2.00, 2.50)	0.51 (-1.17, 2.15)	-0.25 (-2.54, 2.03)	—	-0.61 (-2.28, 1.06)	—	-0.41 (-1.85, 1.02)
	0.16 (-0.26, 0.64)	0.12 (-0.53, 0.85)	**VD**	-1.11 (-2.55, 0.39)	—	—	0.56 (-1.69, 2.78)	0.81 (-0.84, 2.42)	0.06 (-2.22, 2.30)	—	-0.31 (-1.95, 1.32)	—	-0.11 (-1.51, 1.28)
	-0.69 (-2.91, 1.59)	-0.73 (-3.01, 1.59)	-0.86 (-3.13, 1.45)	**Probiotics**	—	—	1.67 (-0.64, 3.90)	**1.92 (0.20, 3.55)**	1.17 (-1.18, 3.42)	—	0.81 (-0.92, 2.45)	—	1.00 (-0.49, 2.41)
	—	—	—	—	**Probiotics ＋VD**	—	—	—	—	—	—	—	—
	—	—	—	—	—	**VD＋Ca**	—	—	—	—	—	—	—
	—	—	—	—	—	—	**α-lipoic acid**	0.25 (-1.72, 2.21)	-0.50 (-2.66, 1.64)	—	-0.86 (-3.23, 1.52)	—	-0.67 (-2.88, 1.55)
	-0.03 (-0.80, 0.73)	-0.07 (-0.99, 0.86)	-0.19 (-1.11, 0.66)	0.66 (-1.73, 3.00)	—	—	—	**Omega-3**	-0.75 (-2.75, 1.24)	—	-1.11 (-2.92, 0.72)	—	-0.91 (-2.52, 0.69)
	—	—	—	—	—	—	—	—	**Omega-3 ＋α-lipoic acid**	—	-0.36 (-2.76, 2.05)	—	-0.16 (-2.41, 2.09)

	—	—	—	—	—	—	—	—		**Probiotics ＋α-lipoic acid**	—	—	—
	-0.20 (-0.70, 0.30)	-0.24 (-0.96, 0.48)	-0.37 (-1.07, 0.28)	0.49 (-1.84, 2.76)	—	—	—	-0.17 (-1.08, 0.74)	—	—	**Curcumin**	—	0.20 (-1.42, 1.80)
	—	—	—	—	—	—	—	—	—	—	—	**Probiotics ＋Omega-3**	—
	0.20 (-0.68, 1.09)	0.16 (-0.86, 1.19)	0.04 (-0.98, 1.01)	0.89 (-1.55, 3.28)	—	—	—	0.23 (-0.93, 1.40)	—	—	0.40 (-0.61, 1.42)	—	**Mg**

Data are reported as mean difference (95% credible intervals) and indicate column-to-row differences. Statistically significant differences are in bold and inclined formats (*P*-values <0.05).

HOMA-IR: Homeostatic model assessment of insulin resistance; HbA1c: Hemoglobin A1c; VD: Vitamin D; Ca: Calcium; Mg: Magnesium..

Supplementary Table 6

Results of the network meta-analysis on HDL-C and LDL-C.

	HDL-C												
LDL-C	**Placebo**	-1.26 (-4.35, 1.93)	1.96 (-0.34, 4.29)	0.96 (-1.26, 2.98)	0.22 (-22.50, 22.90)	0.67 (-4.03, 5.38)	-2.57 (-10.14, 5.04)	-0.14 (-2.19, 1.92)	0.04 (-8.35, 8.46)	—	2.76 (-0.12, 5.84)	**5.09 (0.77, 9.38)**	2.20 (-0.88, 5.25)
	-7.21 (-20.49, 4.99)	**Resveratrol**	3.22 (-0.71, 7.10)	2.22 (-1.73, 5.87)	1.46 (-21.53, 24.41)	1.92 (-3.78, 7.55)	-1.31 (-9.49, 6.89)	1.11 (-2.67, 4.83)	1.30 (-7.70, 10.24)	—	4.02 (-0.25, 8.35)	**6.36 (0.92, 11.58)**	3.46 (-1.00, 7.81)
	-0.10 (-8.40, 7.95)	7.12 (-7.52, 22.56)	**VD**	-1.01 (-4.25, 2.04)	-1.74 (-24.53, 20.99)	-1.30 (-6.17, 3.54)	-4.54 (-12.45, 3.40)	-2.11 (-5.22, 0.99)	-1.93 (-10.65, 6.81)	—	0.80 (-2.88, 4.64)	3.13 (-1.80, 7.97)	0.24 (-3.65, 4.04)
	-6.41 (-14.42, 1.51)	0.77 (-13.63, 16.28)	-6.34 (-17.40, 4.96)	**Probiotics**	-0.71 (-23.47, 22)	-0.29 (-5.37, 4.94)	-3.52 (-11.28, 4.41)	-1.10 (-3.79, 1.78)	-0.91 (-9.49, 7.79)	—	1.81 (-1.72, 5.68)	4.13 (-0.21, 8.65)	1.24 (-2.40, 5.03)
	-10.17 (-34.92, 14.22)	-2.91 (-30.32, 25.11)	-10.06 (-35.08, 15.00)	-3.74 (-29.00, 21.13)	**Probiotics ＋VD**	0.45 (-22.71, 23.60)	-2.82 (-26.51, 21.21)	-0.36 (-23.05, 22.41)	-0.20 (-24.30, 24.09)	—	2.58 (-20.32, 25.45)	4.87 (-18.17, 27.89)	1.98 (-20.93, 24.89)
	-6.32 (-22.14, 9.42)	0.89 (-18.90, 21.54)	-6.20 (-22.92, 10.56)	0.12 (-17.45, 17.73)	3.85 (-25.10, 32.95)	**VD＋Ca**	-3.24 (-12.13, 5.68)	-0.81 (-5.95, 4.33)	-0.62 (-10.25, 8.99)	—	2.09 (-3.40, 7.76)	4.43 (-1.96, 10.76)	1.54 (-4.09, 7.12)

	-3.08 (-26.43, 20.46)	4.13 (-22.03, 31.47)	-2.99 (-27.58, 22.03)	3.38 (-21.13, 28.11)	7.10 (-26.68, 41.25)	3.23 (-24.93, 31.66)	**α-lipoic acid**	2.43 (-5.20, 10.01)	2.60 (-6.79, 11.96)	—	5.35 (-2.81, 13.56)	7.66 (-0.97, 16.23)	4.77 (-3.46, 12.92)
	1.81 (-5.52, 9.38)	9.02 (-5.12, 24.43)	1.92 (-8.92, 13.22)	8.23 (-1.98, 18.71)	11.97 (-13.50, 37.89)	8.11 (-9.10, 25.75)	4.89 (-18.58, 28.31)	**omega-3**	0.18 (-8.24, 8.62)	—	2.91 (-0.62, 6.62)	**5.24 (0.78, 9.63)**	2.35 (-1.36, 6.00)
	-3.73 (-30.77, 23.50)	3.49 (-25.90, 33.90)	-3.62 (-31.69, 24.93)	2.68 (-25.42, 31.06)	6.46 (-30.27, 43.22)	2.61 (-28.67, 34.02)	-0.63 (-29.45, 28.18)	-5.56 (-32.59, 21.63)	**omega-3 ＋α-lipoic acid**	—	2.73 (-6.13, 11.67)	5.06 (-4.31, 14.37)	2.16 (-6.81, 11.07)
	—	—	—	—	—	—	—	—	—	**Probiotics ＋α-lipoic acid**	—	—	—
	-0.09 (-10.58, 10.70)	7.12 (-8.93, 24.50)	0.00 (-13.15, 13.15)	6.34 (-6.88, 19.84)	10.10 (-16.38, 37.13)	6.24 (-12.58, 25.41)	2.99 (-22.78, 28.71)	-1.91 (-14.85, 11.13)	3.65 (-25.40, 32.76)	—	**Curcumin**	2.34 (-3.05, 7.43)	-0.56 (-4.94, 3.60)
	-12.49 (-29.61, 4.61)	-5.38 (-25.91, 16.73)	-12.43 (-31.09, 6.63)	-6.08 (-23.52, 11.45)	-2.32 (-31.95, 27.40)	-6.18 (-29.41, 17.17)	-9.44 (-38.21, 19.18)	-14.31 (-31.87, 3.10)	-8.75 (-40.58, 22.76)	—	-12.41 (-32.67, 7.62)	**Probiotics ＋omega-3**	-2.89 (-8.15, 2.39)
	-3.93 (-15.35, 7.86)	3.27 (-13.33, 21.3)	-3.82 (-17.78, 10.71)	2.49 (-11.34, 16.78)	6.25 (-20.74, 33.71)	2.40 (-16.94, 22.13)	-0.85 (-26.88, 25.28)	-5.74 (-19.45, 8.14)	-0.18 (-29.69, 29.30)	—	-3.82 (-19.52, 11.83)	8.59 (-11.93, 29.46)	**Mg**

Data are reported as mean difference (95% credible intervals) and indicate column-to-row differences (i.e. compared with Placebocebo, probiotics＋omega-3 increase HDL-C by 5.09 mg/dL). . Statistically significant differences are in bold and inclined formats (P-values <0.05).

HDL-C: High-density lipoprotein cholesterol; LDL-C: Low-density lipoprotein cholesterol; VD: Vitamin D; Ca: Calcium; Mg: Magnesium..

Supplementary Table 7

Results of the network meta-analysis on Weight and WC.

Weight													
WC	**Placebo**	-1.02 (-8.21, 6.13)	-0.89 (-1.91, 0.19)	-1.81 (-3.68, 0.09)	-1.32 (-6.18, 3.5)	-0.67 (-4.71, 3.41)	-1.53(-5.31,2.27)	0.15(-1.51,1.77)	-0.98(-8.37,6.27)	-3.59 (-8.77,1.62)	-0.5 (-3.59,7.56)	—	—
	0.70 (-4.96, 6.34)	**Resveratrol**	0.15 (-7.08, 7.38)	-0.8 (-8.21, 6.66)	-0.29 (-9.01, 8.31)	0.35 (-7.87, 8.6)	-0.49(-8.67,7.7)	1.17(-6.17,8.53)	0.02 (-10.27,10.33)	-2.57 (-11.50,6.43)	0.53(-7.37,8.38)	—	—
	0.73 (-1.34, 2.78)	0.03 (-5.98, 6.06)	**VD**	-0.93 (-3.06, 1.22)	-0.44 (-5.33, 4.39)	0.21 (-3.89,4.33)	-0.65 (-4.58,3.29)	1.04 (-0.96,2.94)	-0.1 (-7.56,7.23)	-2.71 (-8,2.59)	0.38 (-2.89,3.6)	—	—
	1.75 (-0.84, 4.1)	1.03 (-5.23, 7.15)	1.01 (-2.21, 4.08)	**Probiotics**	0.49 (-4.57, 5.52)	1.13 (-3.34, 5.62)	0.28 (-3.6, 4.17)	1.97 (-0.56, 4.39)	0.83 (-6.72, 8.23)	-1.78 (-6.98, 3.42)	1.31 (-2.32, 4.92)	—	—
	-1.35 (-6.53, 3.79)	-2.06 (-9.68, 5.6)	-2.09 (-7.44, 3.29)	-3.1 (-8.32, 2.33)	**Probiotics ＋VD**	0.65 (-5.63, 6.97)	-0.21 (-6.3, 5.89)	1.47 (-3.65, 6.56)	0.32 (-8.44, 9.16)	-2.26 (-9.35, 4.76)	0.83 (-4.93, 6.51)	—	—
	-0.76 (-7.29, 5.72)	-1.47 (-10.08, 7.16)	-1.49 (-7.89, 4.87)	-2.51 (-9.36, 4.49)	0.58 (-7.61, 8.79)	**VD＋Ca**	-0.85 (-6.44, 4.72)	0.82 (-3.57, 5.17)	-0.3 (-8.73, 8.01)	-2.93 (-9.46, 3.67)	0.17 (-4.95, 5.23)	—	—
	-0.34 (-4.21, 3.34)	-1.07 (-7.85, 5.71)	-1.09 (-5.41, 3.14)	-2.13 (-6.04, 1.96)	0.99 (-5.31, 7.2)	0.4 (-7.14, 7.9)	**α-lipoic acid**	1.68 (-2.37, 5.67)	0.53 (-7.07, 8.13)	-2.06 (-7.54, 3.4)	1.03 (-3.89, 5.96)	—	—
	0.19 (-2.01, 2.36)	-0.51 (-6.57, 5.56)	-0.54 (-3.54, 2.49)	-1.56 (-4.7, 1.78)	1.54 (-4.06, 7.15)	0.94 (-5.91, 7.84)	0.54 (-3.54, 4.73)	**omega-3**	-1.13 (-8.51, 6.16)	-3.75 (-9.1, 1.73)	-0.65 (-4.13, 2.83)	—	—
	0.33 (-6.47, 7.17)	-0.37 (-9.19, 8.55)	-0.4 (-7.49, 6.77)	-1.39 (-8.45, 5.8)	1.7 (-6.85, 10.24)	1.1 (-8.34, 10.5)	0.71 (-6.28, 7.77)	0.15 (-6.66, 7)	**omega-3＋α-lipoic acid**	-2.59 (-11.32, 6.17)	0.48 (-7.39,8.46)	—	—

	3.22 (-1.3, 7.53)	2.49 (-4.69, 9.6)	2.47 (-2.42, 7.26)	1.42 (-2.98, 6.01)	4.56 (-2.16, 11.13)	3.96 (-3.86, 11.77)	3.56 (-1.24, 8.34)	3.02 (-1.9, 7.81)	2.85 (-5.02, 10.62)	**Probiotics ＋α-lipoic acid**	3.09 (-2.93,9.07)	—	—
	1.92 (-1.64, 5.52)	1.22 (-5.46, 7.93)	1.19 (-2.92, 5.35)	0.17 (-4.07, 4.67)	3.26 (-2.96, 9.61)	2.69 (-4.7, 10.16)	2.27 (-2.85, 7.56)	1.73 (-2.44, 5.97)	1.6 (-6.15, 9.33)	-1.29 (-6.87, 4.47)	**Curcumin**	—	—
	—	—	—	—	—	—	—	—	—	—	—	**Probiotics ＋omega-3**	—
	0.32 (-6.75, 7.37)	-0.39 (-9.45, 8.66)	-0.41 (-7.77, 6.92)	-1.41 (-8.84, 6.16)	1.67 (-7.11, 10.46)	1.08 (-8.51, 10.69)	0.69 (-7.27, 8.75)	0.12 (-7.27, 7.52)	-0.05 (-9.89, 9.79)	-2.88 (-11.14, 5.48)	-1.61 (-9.56, 6.32)	—	**Mg**

Data are reported as mean difference (95% credible intervals) and indicate column-to-row differences. Statistically significant differences are in bold and inclined formats (*P*-values <0.05).

WC: Waist circumference; VD: Vitamin D; Ca: Calcium; Mg: Magnesium.

Supplementary Table 8

Results of the network meta-analysis on BMI.

**BMI**	**Placebo**								
	-0.39 (-5.54, 4.78)	**Resveratrol**							
	-0.34 (-2.88, 2.18)	0.04 (-5.72, 5.77)	**VD**						
	-0.65 (-3.89, 2.55)	-0.27 (-6.37, 5.81)	-0.31 (-4.32, 3.66)	**Probiotics**					
	-0.01 (-8.48, 8.46)	0.37 (-9.56, 10.3)	0.32 (-8.28, 8.92)	0.65 (-8.04, 9.29)	**Probiotics+VD**				
	-0.26 (-7.01, 6.52)	0.12 (-8.36, 8.62)	0.08 (-6.88, 7.06)	0.41 (-7.07, 7.88)	-0.24 (-11.02, 10.52)	**VD+Ca**			
	-5.16 (-10.47, 0.16)	-4.76 (-12.22, 2.63)	-4.81 (-10.66, 1.06)	-4.51 (-10.39, 1.42)	-5.13 (-15.11, 4.82)	-4.89 (-13.51, 3.66)	**α-lipoic acid**		
	2.61 (-0.2, 5.45)	3.00 (-2.9, 8.89)	2.96 (-0.82, 6.75)	3.27 (-0.94, 7.53)	2.61 (-6.28, 11.57)	2.86 (-4.44, 10.26)	**-7.76 (2.22, 13.33)**	**omega-3**	
	**-6.70 (-13.13, -0.24)**	-6.33 (-14.57, 1.96)	-6.36 (-13.27, 0.56)	-6.04 (-13.12, 1.07)	-6.68 (-17.31, 3.89)	-6.45 (-15.79, 2.92)	-1.56 (-8.52, 5.44)	**-9.31 (-15.79, -2.81)**	**omega-3+α-lipoic**

									**acid**				
	-2.68 (-11.22, 5.92)	-2.29 (-12.25, 7.74)	-2.33 (-11.23, 6.61)	-2.02 (-10.72, 6.71)	-2.64 (-14.58, 9.28)	-2.43 (-13.32, 8.52)	2.47 (-6.55, 11.51)	-5.29 (-14.19, 3.66)	4.04 (-6.33, 14.37)	**Probiotics ＋α-lipoic acid**			
	-0.13 (-4.32, 4.09)	0.26 (-6.4, 6.9)	0.21 (-4.69, 5.16)	0.53 (-4.75, 5.84)	-0.12 (-9.56, 9.37)	0.12 (-7.85, 8.16)	5.02 (-1.72, 11.8)	-2.74 (-7.79, 2.34)	6.58 (-1.14, 14.26)	2.55 (-7.02, 12.11)	**Curcumin**		
	—	—	—	—	—	—	—	—	—	—	—	**Probiotics ＋omega-3**	
	0.03 (-5.86, 5.89)	0.41 (-7.44, 8.21)	0.37 (-6.03, 6.76)	0.68 (-6.04, 7.37)	0.04 (-10.26, 10.3)	0.27 (-8.68, 9.23)	5.18 (-2.76, 13.07)	-2.58 (-9.15, 3.95)	6.72 (-1.98, 15.43)	2.69 (-7.7, 13.09)	0.15 (-7.1, 7.39)	—	**Mg**

Data are reported as mean difference (95% credible intervals) and indicate column-to-row differences. Statistically significant differences are in bold and inclined formats (*P*-values <0.05).

BMI: Body mass index; VD: Vitamin D; Ca: Calcium; Mg: Magnesium.

Supplementary Figure 1

Risk of bias graph

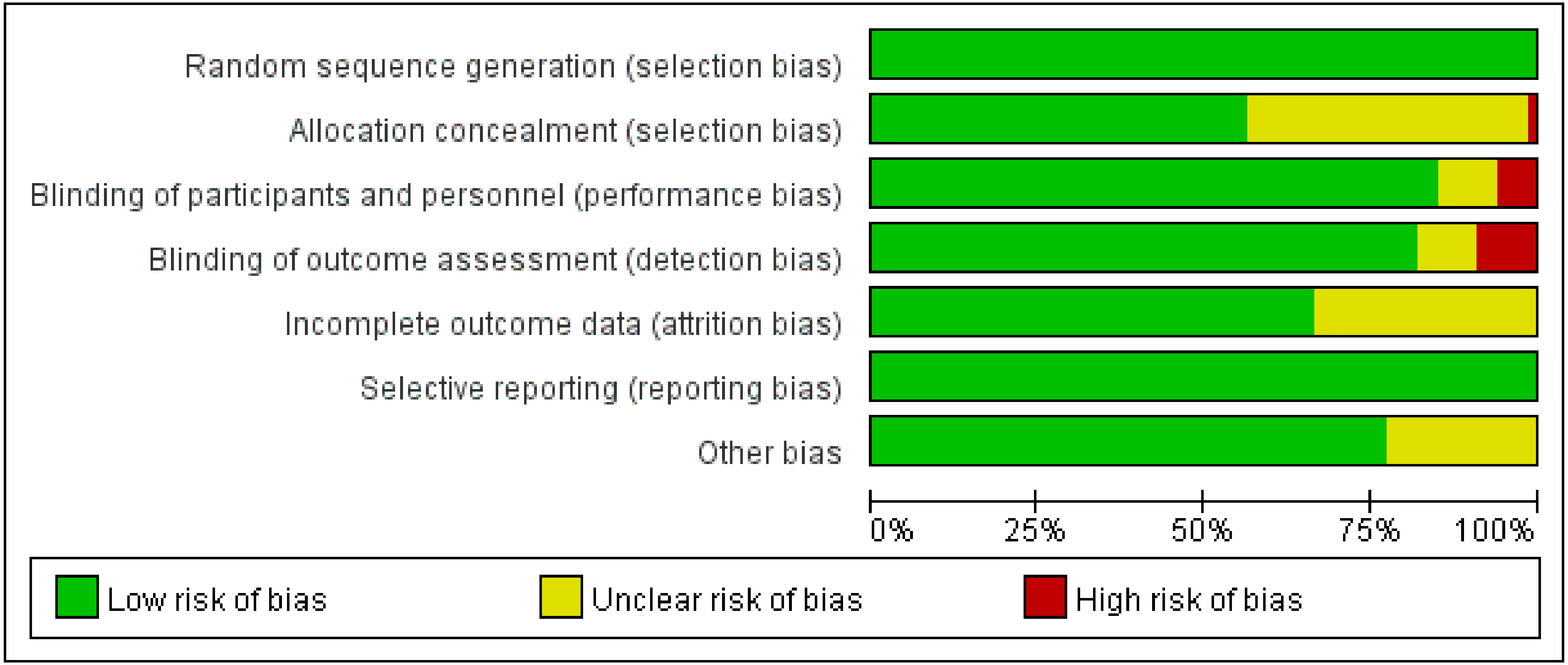

Risk of bias table

StudyID	Adequate sequence generation	Adequate allocation concealment	Blinding-participant	Blinding-therapist	Incomplete outcome data addressed	Free of selective reporting	Other bias
**1. Arzola-Paniagua 2016**	Low risk	Low risk	Low risk	Low risk	Low risk	Low risk	Unclear risk
**2. Abbott 2020**	Low risk	Low risk	Low risk	Low risk	Low risk	Low risk	Unclear risk
**3. Al-Bayyari 2018**	Low risk	Low risk	Low risk	Low risk	Low risk	Low risk	Low risk
**4. Anggeraini 2021**	Low risk	Unclear risk	Low risk	Low risk	Unclear risk	Low risk	Unclear risk
**5. Bateni 2021**	Low risk	Low risk	Low risk	Low risk	Low risk	Low risk	Low risk
**6. Batista-Jorge 2020**	Low risk	Unclear risk	Unclear risk	Unclear risk	Low risk	Low risk	Unclear risk
**7. Baxheinrich 2012**	Low risk	Unclear risk	Unclear risk	Unclear risk	Low risk	Low risk	Unclear risk
**8. Browning 2007**	Low risk	Unclear risk	Unclear risk	Unclear risk	Low risk	Low risk	Low risk
**9. Campbell 2019**	Low risk	Unclear risk	Low risk	Low risk	Low risk	Low risk	Low risk

**10. Carrillo 2013**	Low risk	Unclear risk	Low risk	Low risk	Low risk	Low risk	Low risk
**11. Chacko 2011**	Low risk	Low risk	Low risk	Low risk	Low risk	Low risk	Low risk
**12. Chandler 2015**	Low risk	Low risk	Low risk	Low risk	Low risk	Low risk	Low risk
**13. Cheshmazar 2020**	Low risk	Low risk	Low risk	Low risk	Low risk	Low risk	Low risk
**14. Cicero 2020**	Low risk	Low risk	Low risk	Low risk	Low risk	Low risk	Low risk
**15. De Luis 2016**	Low risk	Unclear risk	Low risk	High risk	Unclear risk	Low risk	Unclear risk
**16. DeFina 2011**	Low risk	Unclear risk	Low risk	High risk	Unclear risk	Low risk	Unclear risk
**17. Dolati 2020**	Low risk	Unclear risk	Low risk	Low risk	Low risk	Low risk	Low risk
**18. Ebadi 2021**	Low risk	Low risk	Low risk	Low risk	Unclear risk	Low risk	Low risk
**19. Eslamparast 2014**	Low risk	Low risk	Low risk	Low risk	Low risk	Low risk	Low risk
**20. Farag 2018**	Low risk	Unclear risk	Unclear risk	Unclear risk	Low risk	Low risk	Unclear risk
**21. Gammelmark 2012**	Low risk	Unclear risk	Low risk	Low risk	Low risk	Low risk	Low risk
**22. Hadi 2019**	Low risk	Low risk	Low risk	Low risk	Low risk	Low risk	Low risk

**23. Hajipoor 2020**	Low risk	Low risk	Low risk	Low risk	Unclear risk	Low risk	Low risk
**24. Hess 2020**	Low risk	Low risk	Low risk	Low risk	Low risk	Low risk	Low risk
**25. Huerta 2015**	Low risk	Low risk	Low risk	Low risk	Low risk	Low risk	Low risk
**26. Jaacks 2018**	Low risk	Unclear risk	High risk	High risk	Low risk	Low risk	Unclear risk
**27. Javandoost 2018**	Low risk	Low risk	Low risk	Low risk	Low risk	Low risk	Low risk
**28. Joris 2016**	Low risk	Low risk	Low risk	Low risk	Unclear risk	Low risk	Low risk
**29. Joris 2017**	Low risk	Unclear risk	Low risk	Low risk	Low risk	Low risk	Low risk
**30. Kantartzis 2018**	Low risk	Low risk	Low risk	Low risk	Low risk	Low risk	Low risk
**31. Karandish 2021**	Low risk	Low risk	Low risk	Low risk	Low risk	Low risk	Low risk
**32. Kratz 2008**	Low risk	Unclear risk	High risk	High risk	Low risk	Low risk	Unclear risk
**33. Lee 2009**	Low risk	Low risk	Low risk	Low risk	Low risk	Low risk	Low risk
**34. Lithgow 2018**	Low risk	Low risk	Low risk	Low risk	Low risk	Low risk	Low risk
**35. Mai 2017**	Low risk	Unclear risk	Unclear risk	Unclear risk	Low risk	Low risk	Low risk

**36. Major 2007**	Low risk	Unclear risk	Low risk	Low risk	Low risk	Low risk	Low risk
**37. Makariou 2017**	Low risk	Unclear risk	Low risk	Low risk	Unclear risk	Low risk	Low risk
**38. Mason 2014**	Low risk	Low risk	Low risk	Low risk	Low risk	Low risk	Low risk
**39. Mohammadi 2013**	Low risk	Unclear risk	Low risk	Low risk	Low risk	Low risk	Low risk
**40. Mooren 2011**	Low risk	Unclear risk	Low risk	Low risk	Low risk	Low risk	Low risk
**41. Morten M 2013**	Low risk	Low risk	Low risk	Low risk	Low risk	Low risk	Low risk
**42. Munro 2012**	Low risk	Low risk	Low risk	Low risk	Low risk	Low risk	Low risk
**43. Munro 2013**	Low risk	Low risk	Low risk	Low risk	Low risk	Low risk	Low risk
**44. Nasiri 2021**	Low risk	Low risk	Low risk	Low risk	Low risk	Low risk	Low risk
**45. Neale 2013**	Low risk	Unclear risk	High risk	High risk	Unclear risk	Low risk	Unclear risk
**46. Rabiei 2019**	Low risk	Low risk	Low risk	Low risk	Low risk	Low risk	Low risk
**47. Rahayu 2021**	Low risk	Low risk	Low risk	Low risk	Unclear risk	Low risk	Low risk
**48. Rajaie 2018**	Low risk	Low risk	Low risk	Low risk	Low risk	Low risk	Low risk

**49. Rajaie 2021**	Low risk	Low risk	Low risk	Low risk	Low risk	Low risk	Low risk
**50. Rajkumar 2014**	Low risk	Low risk	Low risk	Low risk	Unclear risk	Low risk	Low risk
**51. Rodríguez-Moran 2014**	Low risk	Unclear risk	Low risk	Low risk	Low risk	Low risk	Low risk
**52. Romo-Hualde 2016**	Low risk	High risk	High risk	High risk	Unclear risk	Low risk	Low risk
**53. Romo-Hualde 2018**	Low risk	Unclear risk	Low risk	Low risk	Unclear risk	Low risk	Unclear risk
**54. Roosta, M 2019**	Low risk	Unclear risk	Low risk	Low risk	Unclear risk	Low risk	Low risk
**55. Salehpour 2012**	Low risk	Unclear risk	Low risk	Low risk	Unclear risk	Low risk	Unclear risk
**56. Salekzamani 2016**	Low risk	Low risk	Low risk	Low risk	Unclear risk	Low risk	Low risk
**57. Sanne M 2015**	Low risk	Low risk	Low risk	Low risk	Low risk	Low risk	Low risk
**58. Sjoberg 2010**	Low risk	Unclear risk	Low risk	Low risk	Unclear risk	Low risk	Low risk
**59. Solati 2019**	Low risk	Low risk	Low risk	Low risk	Unclear risk	Low risk	Low risk
**60. Szulińska 2018**	Low risk	Low risk	Low risk	Low risk	Unclear risk	Low risk	Low risk
**61. Timmers 2011**	Low risk	Unclear risk	Low risk	Low risk	Low risk	Low risk	Low risk

**62. Tripolt 2013**	Low risk	Unclear risk	Unclear risk	Unclear risk	Unclear risk	Low risk	Low risk
**63. Wong 2013**	Low risk	Low risk	Low risk	Low risk	Unclear risk	Low risk	Low risk
**64. Yang 2014**	Low risk	Low risk	Low risk	Low risk	Unclear risk	Low risk	Low risk
**65. Zittermann 2009**	Low risk	Unclear risk	Low risk	Low risk	Unclear risk	Low risk	Unclear risk

Supplementary Figure 2

Results of network meta-regression analysis

(a) SBP

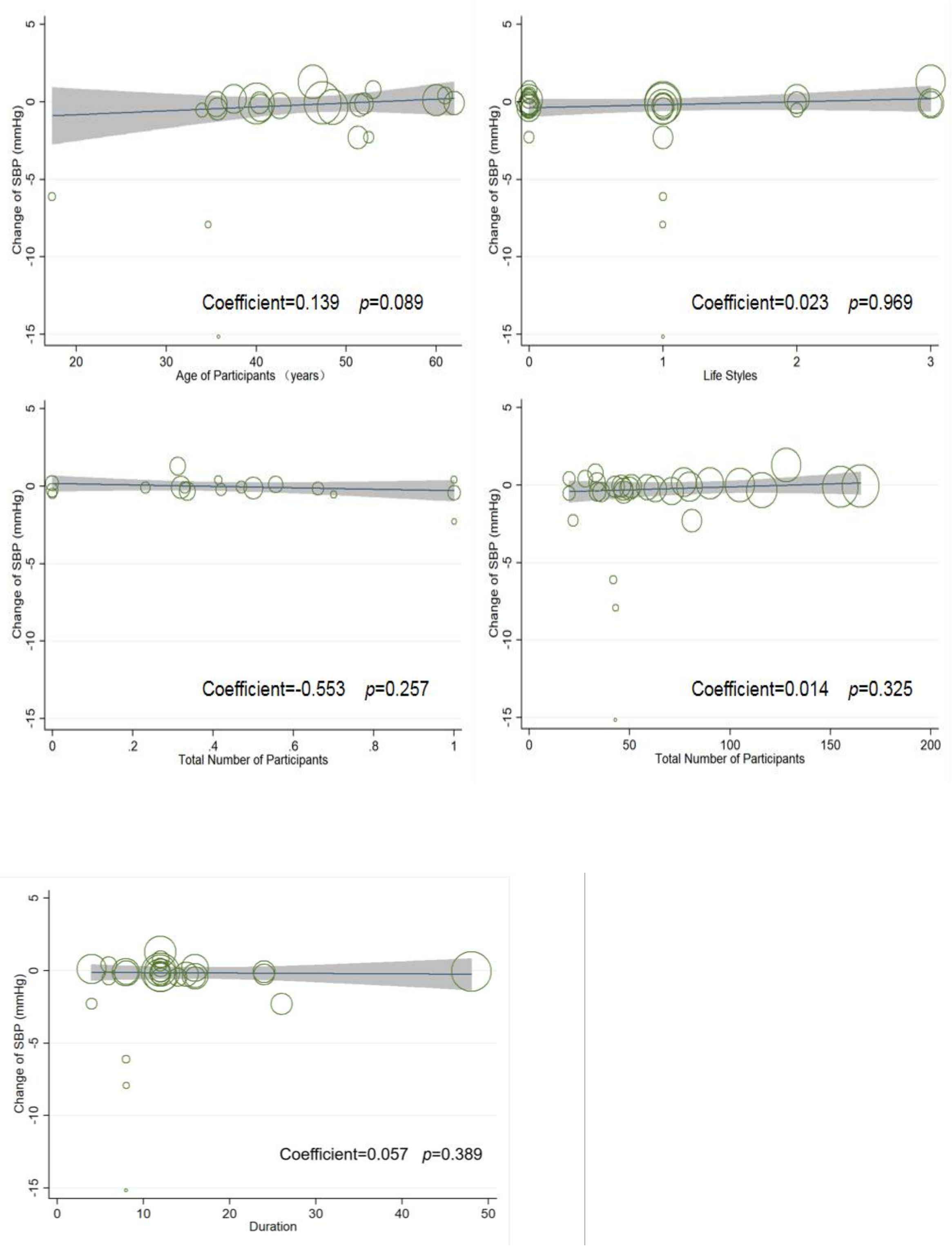

(b) DBP

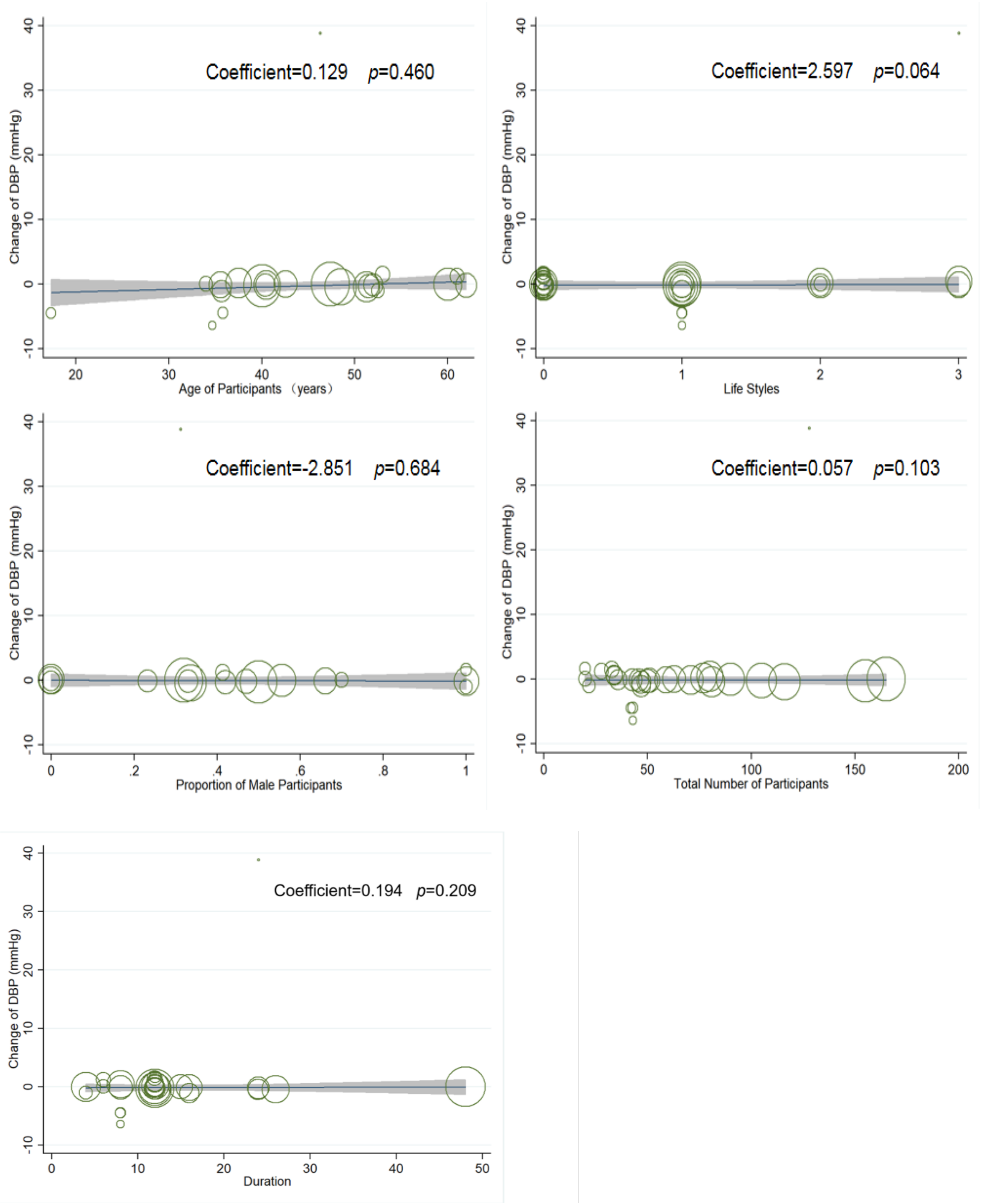
Coefficient=0.129 p=0.460
Change of DBP (mmHg)
Age of Participants （years）
Coefficient=2.597 p=0.064
Change of DBP (mmHg)
Life Styles
Coefficient=-2.851 p=0.684
Change of DBP (mmHg)
Proportion of Male Participants
Coefficient=0.057 p=0.103
Change of DBP (mmHg)
Total Number of Participants
Coefficient=0.194 p=0.209
Change of DBP (mmHg)
Duration

(c) FBG

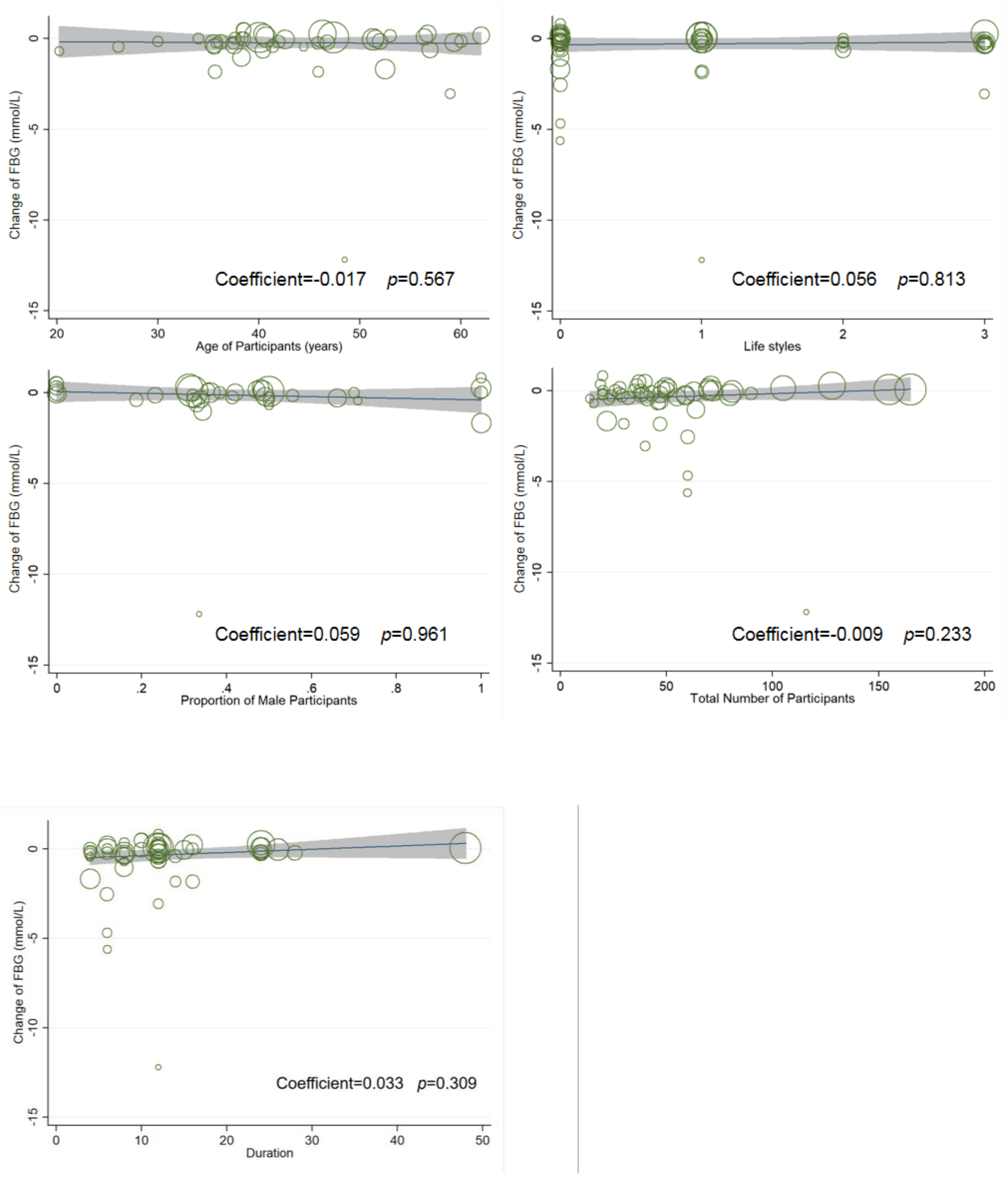

Change of FBG (mmol/L)
Coefficient=-0.017 p=0.567
Age of Participants (years)
Change of FBG (mmol/L)
Coefficient=0.056 p=0.813
Life styles
Change of FBG (mmol/L)
Coefficient=0.059 p=0.961
Proportion of Male Participants
Change of FBG (mmol/L)
Coefficient=-0.009 p=0.233
Total Number of Participants
Change of FBG (mmol/L)
Coefficient=0.033 p=0.309
Duration

(d) FINS

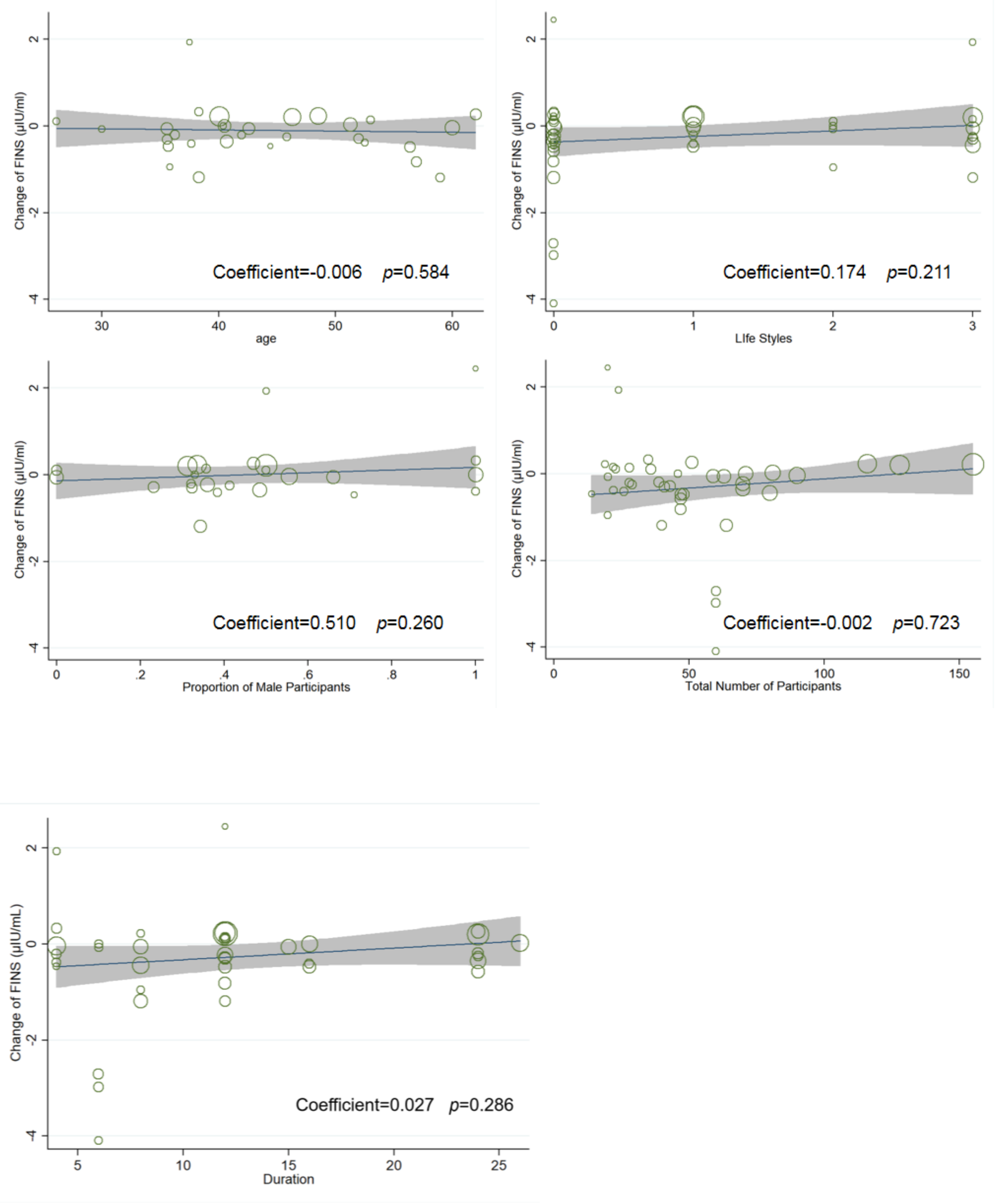
Change of FINS (µIU/ml)
Coefficient=-0.006 p=0.584
age
Change of FINS (µIU/ml)
Coefficient=0.174 p=0.211
LIfe Styles
Change of FINS (µIU/ml)
Coefficient=0.510 p=0.260
Proportion of Male Participants
Change of FINS (µIU/ml)
Coefficient=-0.002 p=0.723
Total Number of Participants
Change of FINS (µIU/mL)
Coefficient=0.027 p=0.286
Duration

(e) HOMA-IR

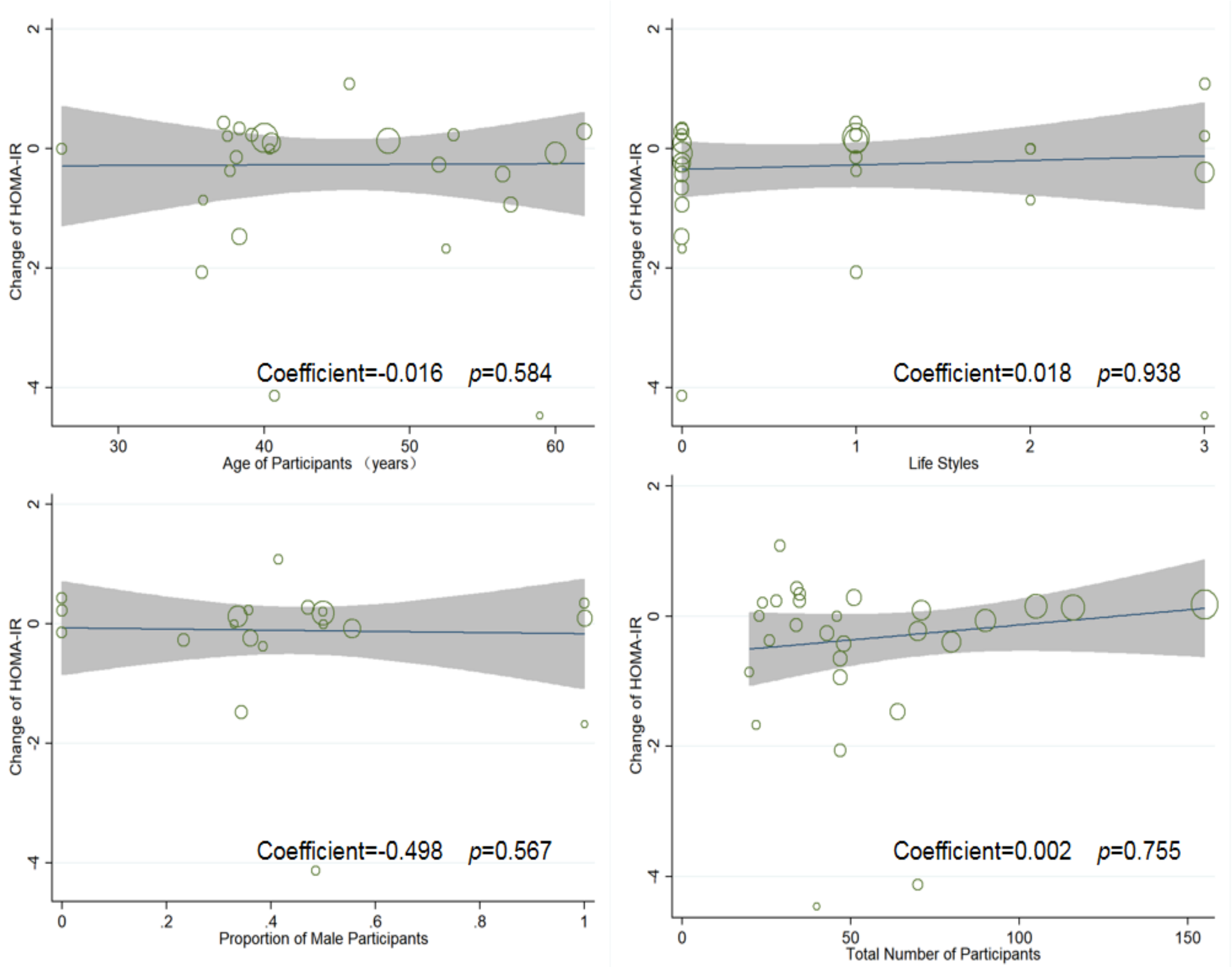

Change of HOMA-IR
Coefficient=-0.016 p=0.584
Age of Participants （years）
Coefficient=0.018 p=0.938
Life Styles
Coefficient=-0.498 p=0.567
Proportion of Male Participants
Coefficient=0.002 p=0.755
Total Number of Participants

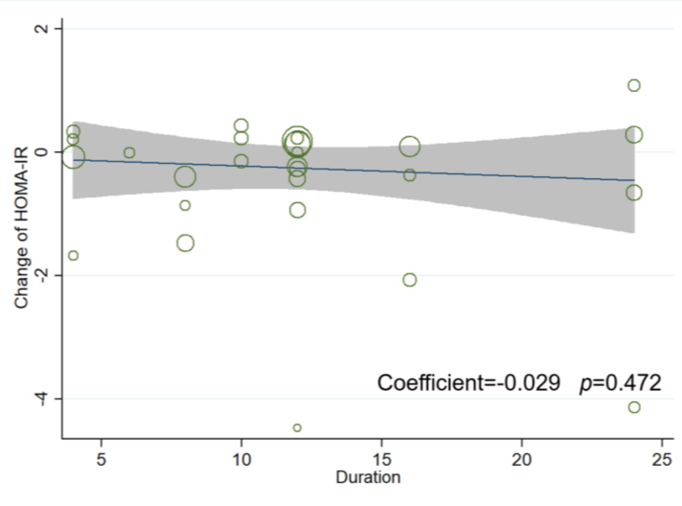

Change of HOMA-IR
Coefficient=-0.029 p=0.472
Duration

(f) HbA1c

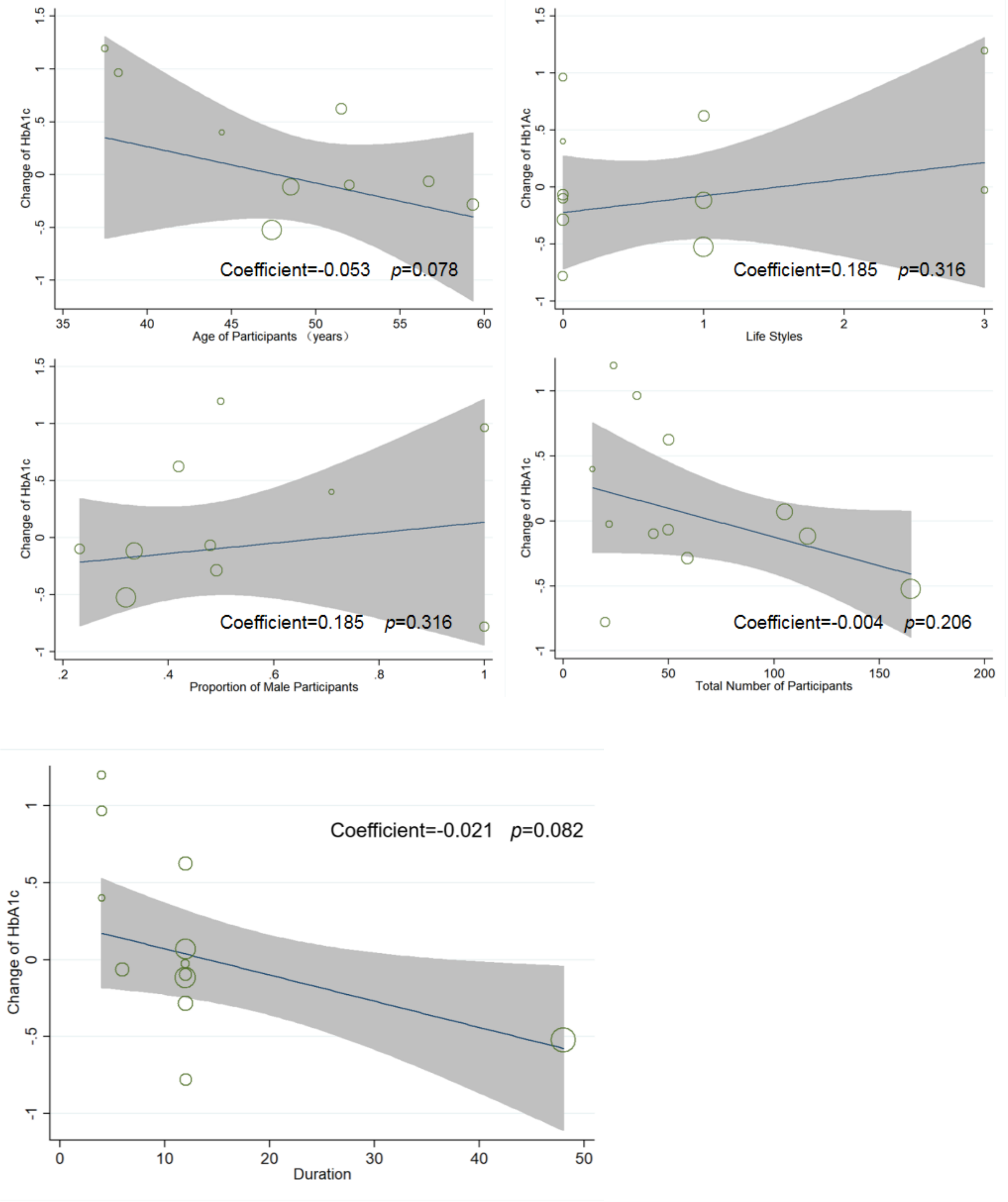
Change of HbA1c
Age of Participants （years）
Coefficient=-0.053 p=0.078
Change of Hb1Ac
Life Styles
Coefficient=0.185 p=0.316
Change of HbA1c
Proportion of Male Participants
Coefficient=0.185 p=0.316
Change of HbA1c
Total Number of Participants
Coefficient=-0.004 p=0.206
Change of HbA1c
Duration
Coefficient=-0.021 p=0.082

(g) TGs

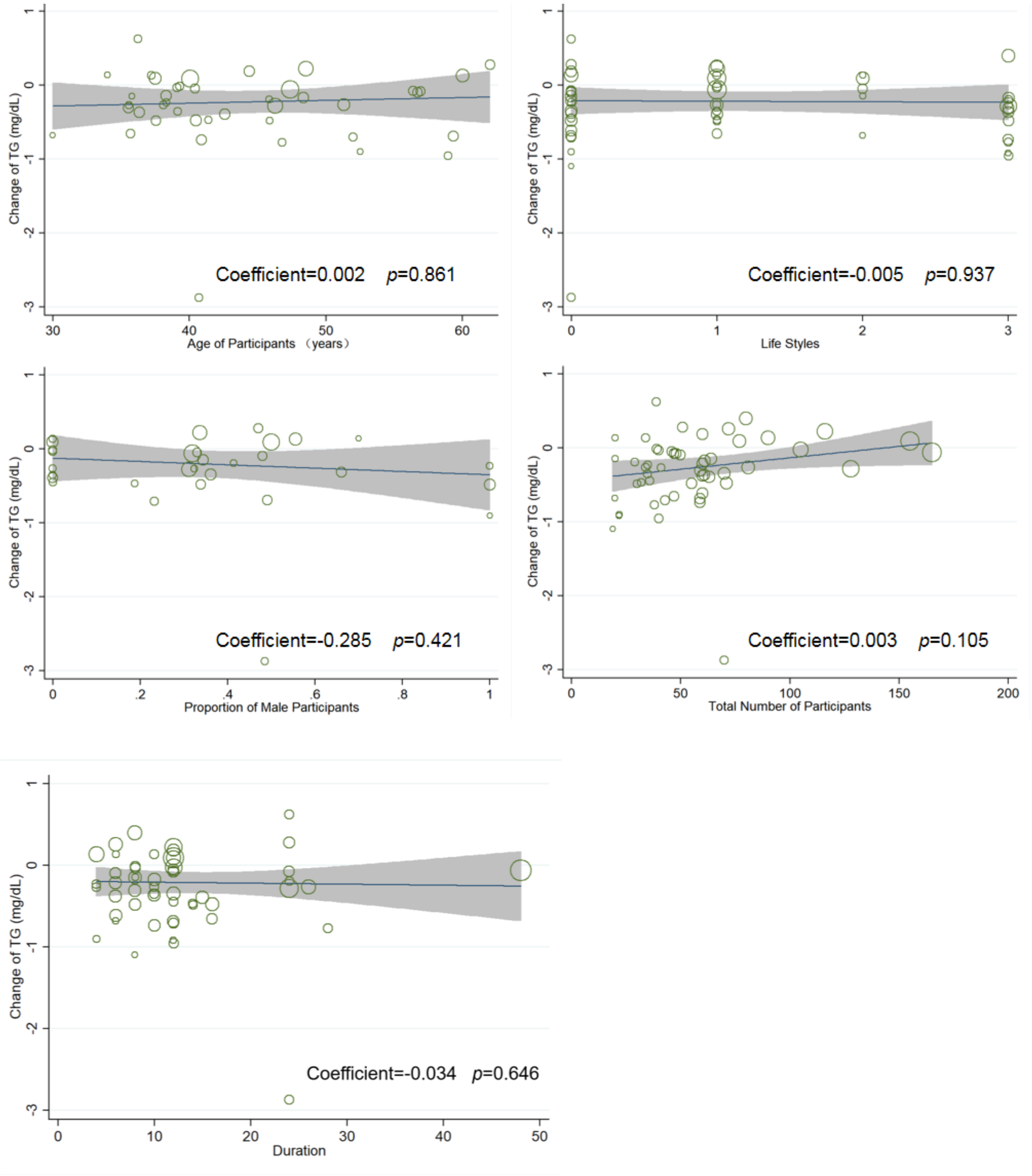
Change of TG (mg/dL)
Coefficient=0.002 p=0.861
Age of Participants （years）
Change of TG (mg/dL)
Coefficient=-0.005 p=0.937
Life Styles
Change of TG (mg/dL)
Coefficient=-0.285 p=0.421
Proportion of Male Participants
Change of TG (mg/dL)
Coefficient=0.003 p=0.105
Total Number of Participants
Change of TG (mg/dL)
Coefficient=-0.034 p=0.646
Duration

(h) TC

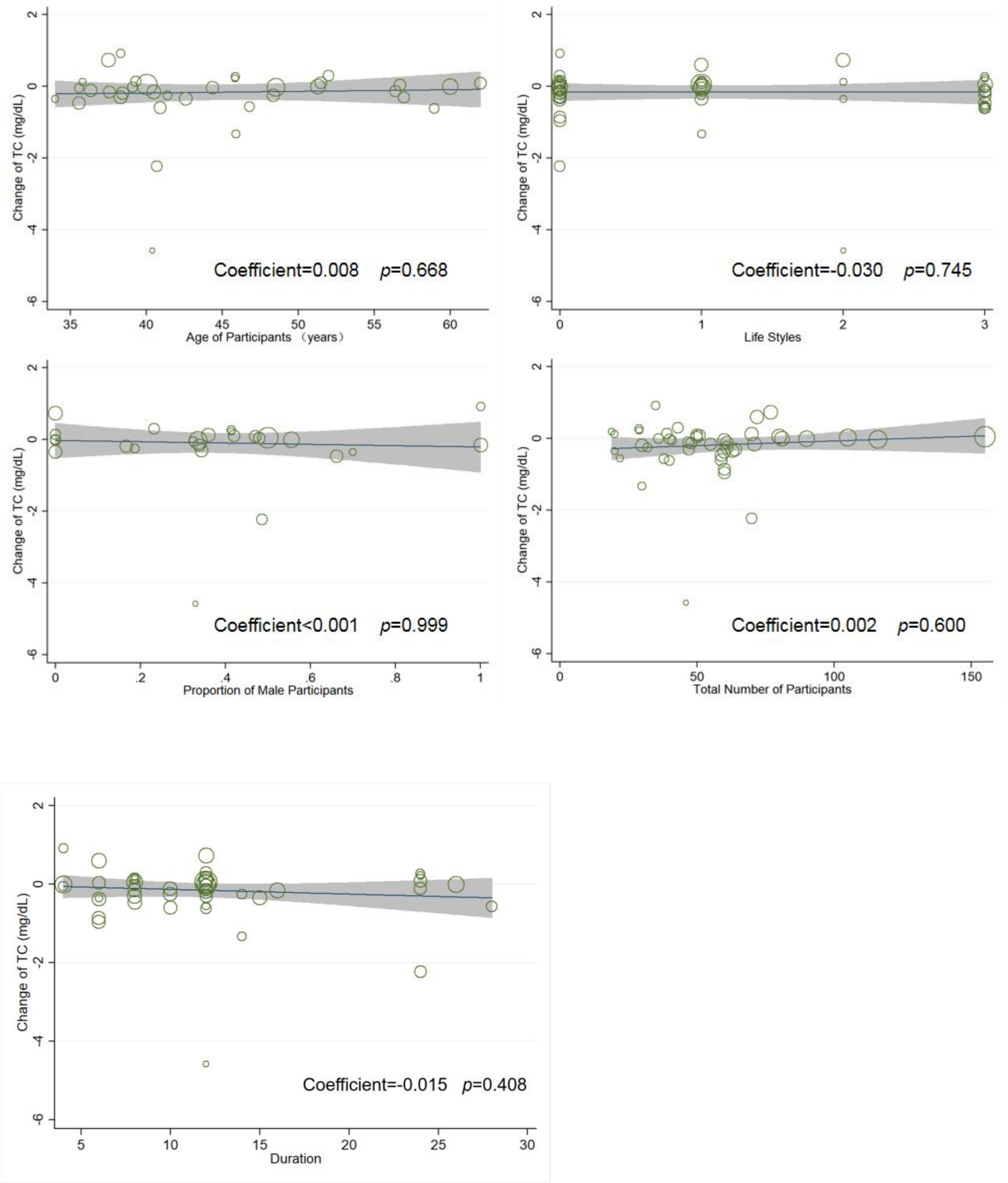
Change of TC (mg/dL)
Age of Participants （years）
Coefficient=0.008 p=0.668
Life Styles
Coefficient=-0.030 p=0.745
Proportion of Male Participants
Coefficient<0.001 p=0.999
Total Number of Participants
Coefficient=0.002 p=0.600
Duration
Coefficient=-0.015 p=0.408

(i) HDL-C

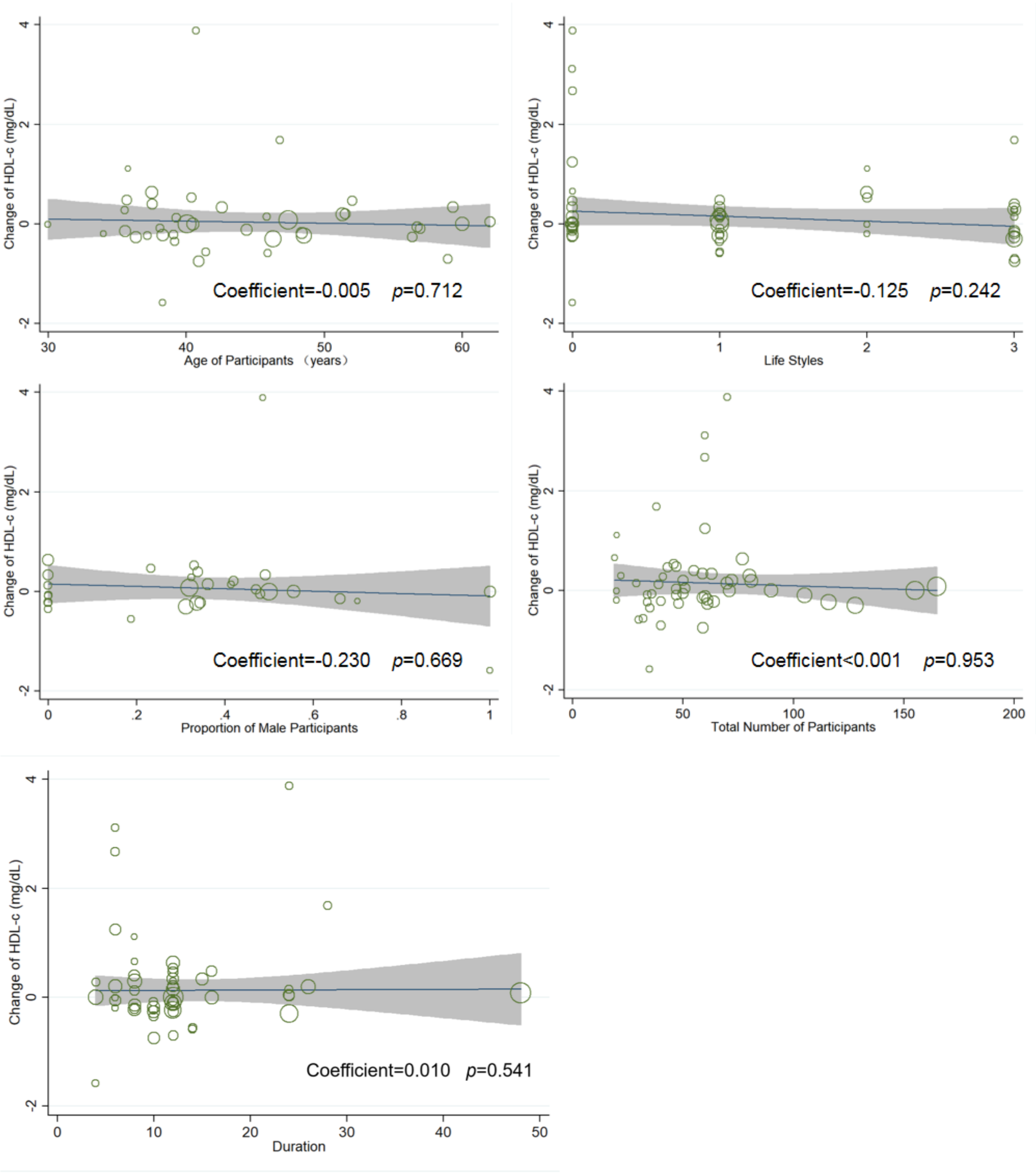
Change of HDL-c (mg/dL)
Coefficient=-0.005 p=0.712
Age of Participants （years）
Change of HDL-c (mg/dL)
Coefficient=-0.125 p=0.242
Life Styles
Change of HDL-c (mg/dL)
Coefficient=-0.230 p=0.669
Proportion of Male Participants
Change of HDL-c (mg/dL)
Coefficient<0.001 p=0.953
Total Number of Participants
Change of HDL-c (mg/dL)
Coefficient=0.010 p=0.541
Duration

(j) LDL-C

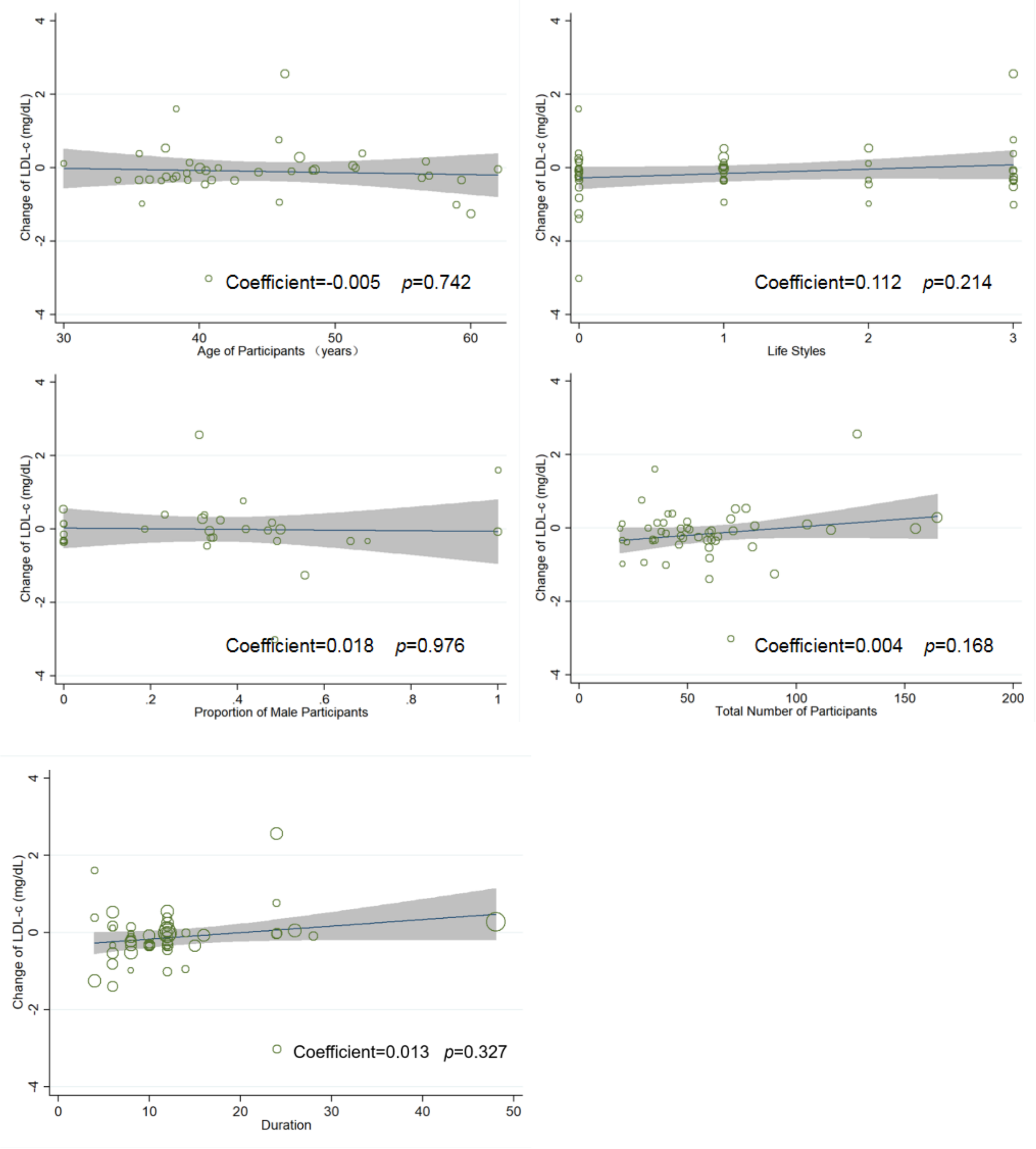
Change of LDL-c (mg/dL)
Coefficient=-0.005 p=0.742
Age of Participants （years）
Coefficient=0.112 p=0.214
Life Styles
Coefficient=0.018 p=0.976
Proportion of Male Participants
Coefficient=0.004 p=0.168
Total Number of Participants
Coefficient=0.013 p=0.327
Duration

(k) Weight

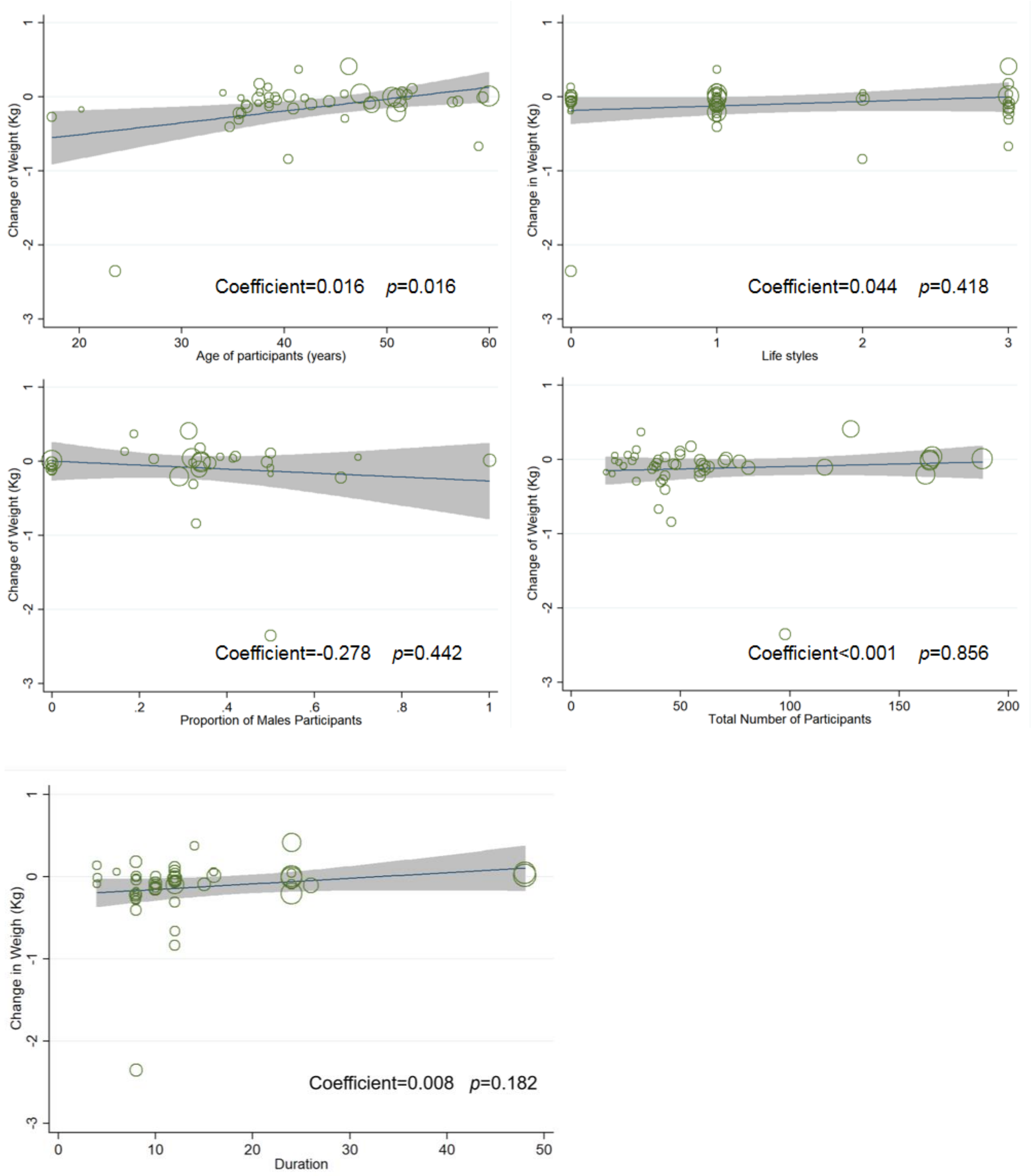
Change of Weight (Kg)
Coefficient=0.016 p=0.016
Age of participants (years)
Change in Weight (Kg)
Coefficient=0.044 p=0.418
Life styles
Change of Weight (Kg)
Coefficient=-0.278 p=0.442
Proportion of Males Participants
Change of Weight (Kg)
Coefficient<0.001 p=0.856
Total Number of Participants
Change in Weigh (Kg)
Coefficient=0.008 p=0.182
Duration

(l) WC

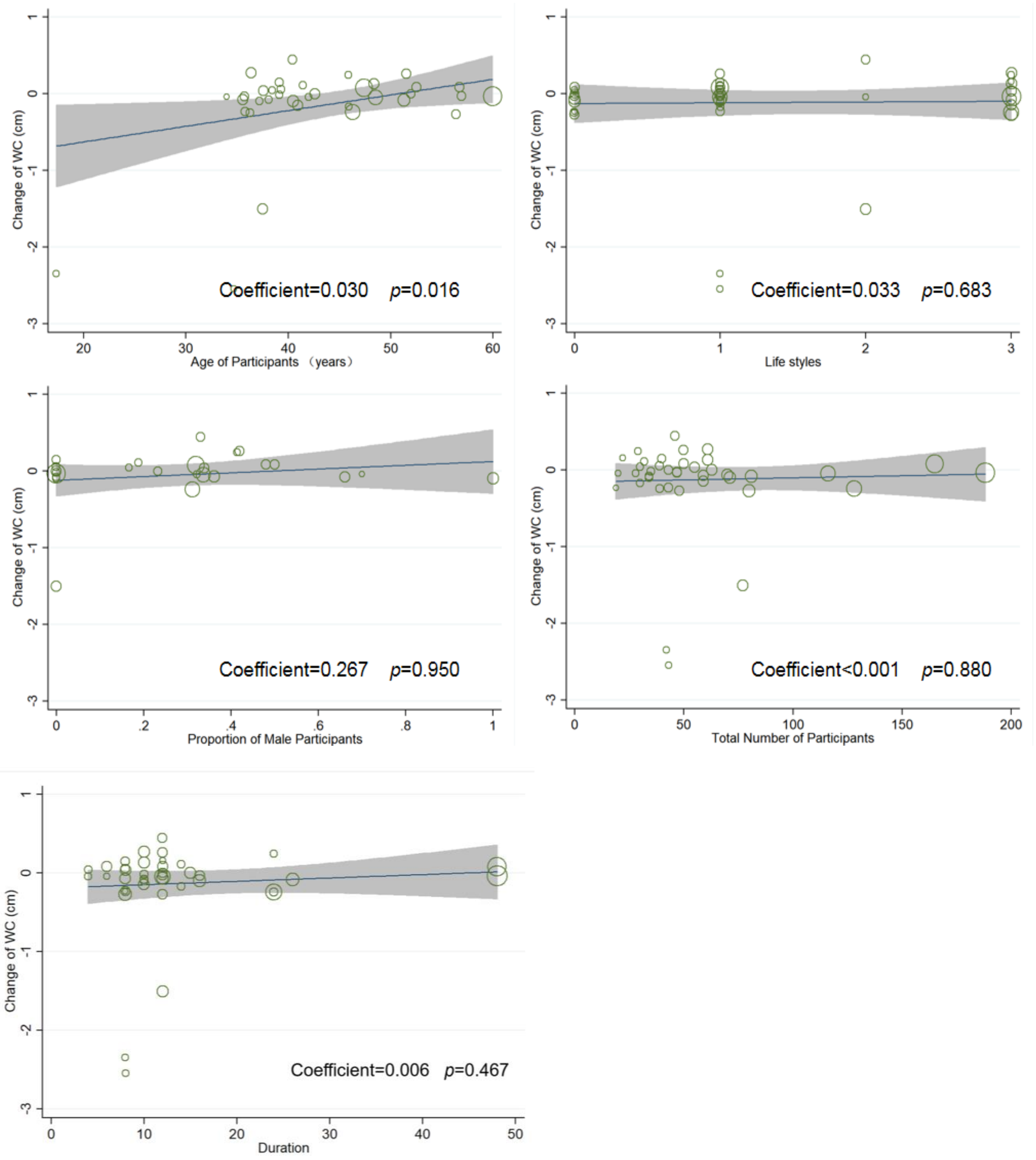
Change of WC (cm)
Coefficient=0.030 p=0.016
Age of Participants （years）
Change of WC (cm)
Coefficient=0.033 p=0.683
Life styles
Change of WC (cm)
Coefficient=0.267 p=0.950
Proportion of Male Participants
Change of WC (cm)
Coefficient<0.001 p=0.880
Total Number of Participants
Change of WC (cm)
Coefficient=0.006 p=0.467
Duration

(m) BMI

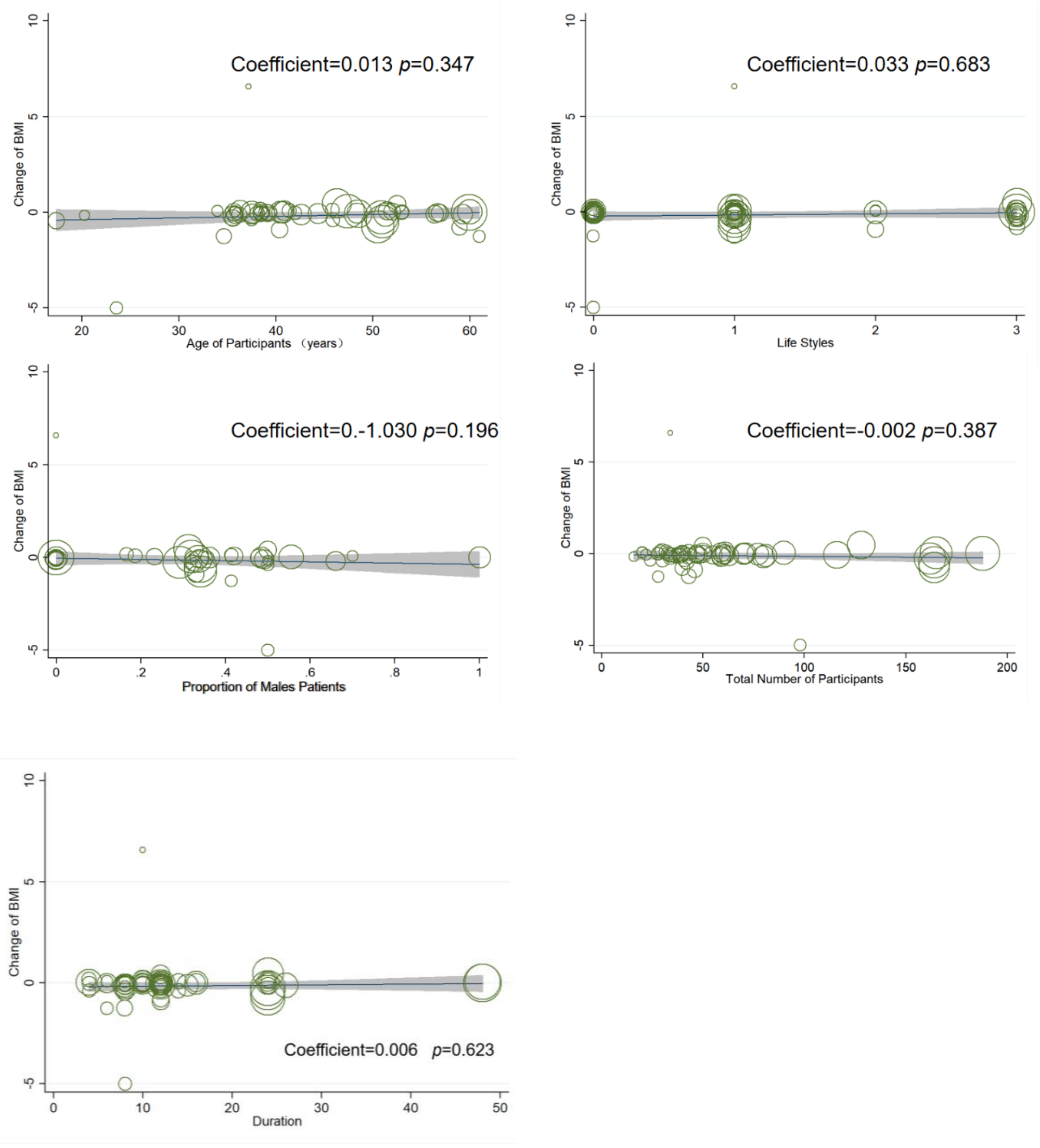

Figure 2 The association between outcomes and age, life style, proportion of males, total number, respectively. (a) SBP; (b) DBP; (c) FBG; (d) FINS; (e) HOMA-IR; (f) HbA1c; (g) TGs (h) TC; (i) HDL-C; (j) LDL-C; (k)Weight; (l) WC; (m) BMI. SBP: systolic blood pressure; DBP: diastolic blood pressure; FBG: fasting blood glucose; FINS: fasting insulin level; HOMA-IR: homeostatic model assessment of insulin resistance; HbA1c: hemoglobin A1c; TGs: triglycerides; TC: total cholesterol; HDL-C: high-density lipoprotein cholesterol; LDL-C: low-density lipoprotein cholesterol; WC: waist circumference; BMI: body mass index;

Supplementary Figure 3

Detailed results of SCURA ranking

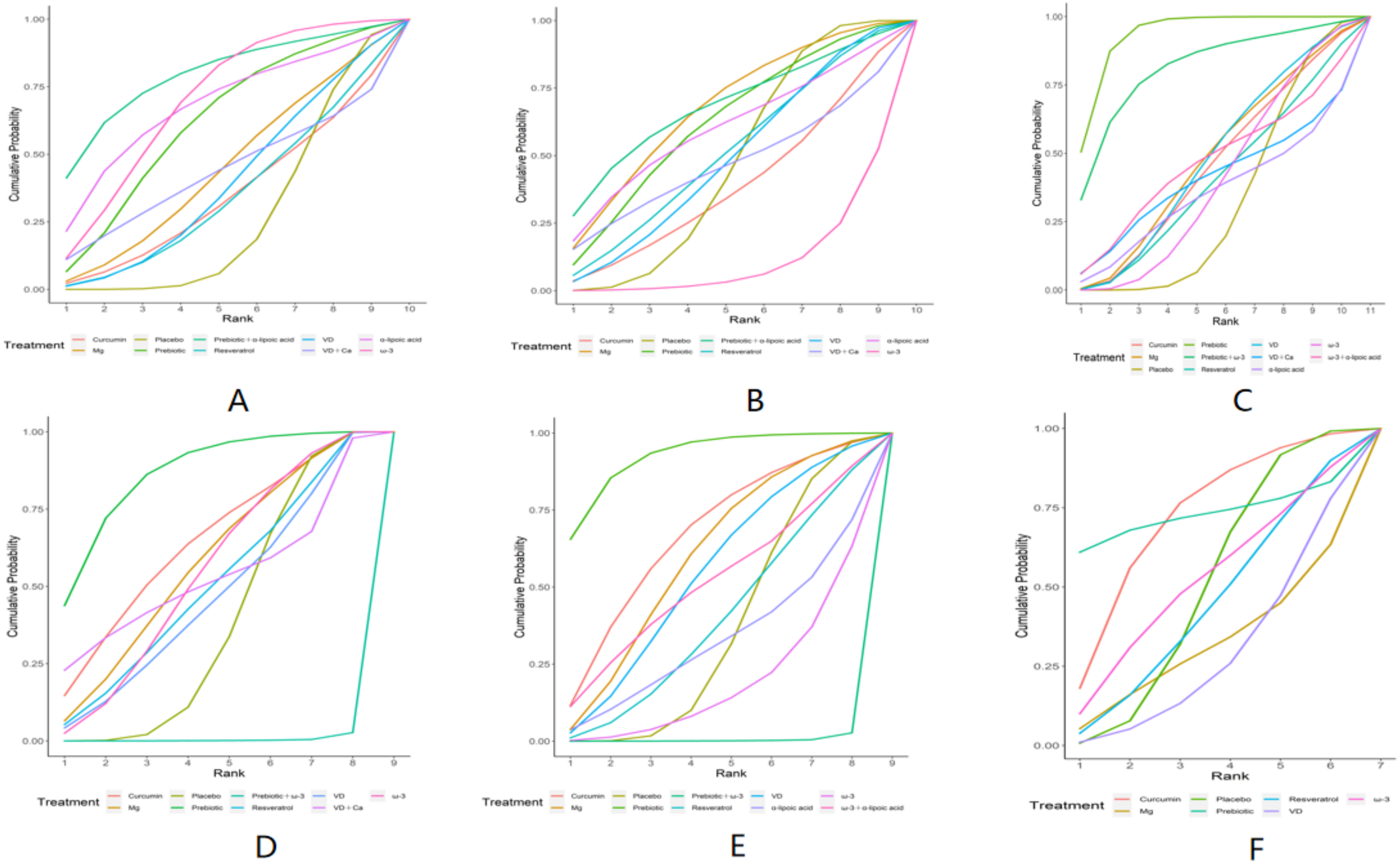

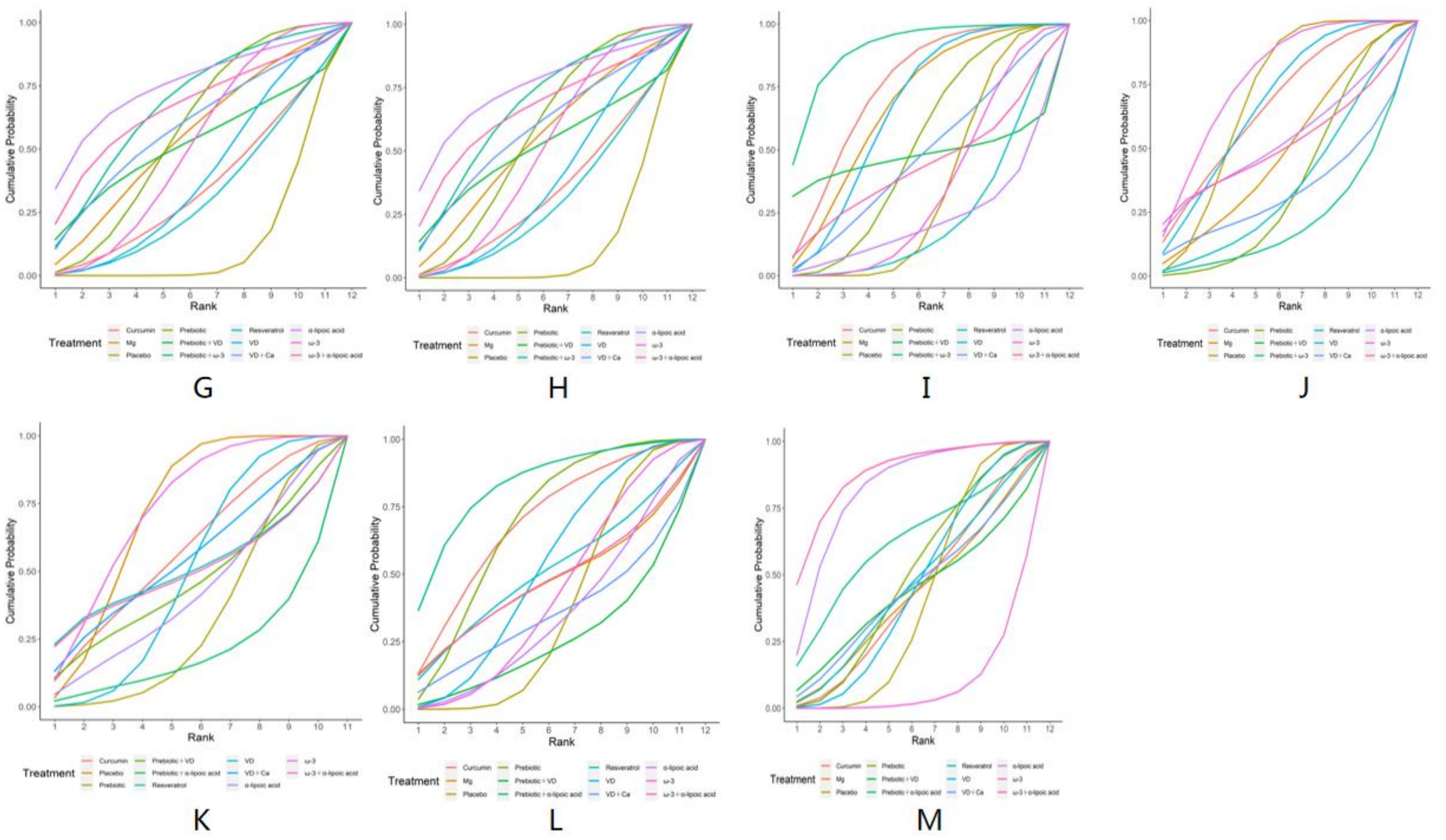

Figure 3 Plots of cumulative ranking probability of outcomes. (A) SBP; (B) DBP; (C) FBG; (D) FINS; (E) HOMA-IR; (F) HbA1c; (G) TGs (H) TC; (I) HDL-C; (J) LDL-C; (K)Weight; (L) WC; (M) BMI. SBP: systolic blood pressure; DBP: diastolic blood pressure; FBG: fasting blood glucose; FINS: fasting insulin level; HOMA-IR: homeostatic model assessment of insulin resistance; HbA1c: hemoglobin A1c; TGs: triglycerides; TC: total cholesterol; HDL-C: high-density lipoprotein cholesterol; LDL-C: low-density lipoprotein cholesterol; WC: waist circumference; BMI: body mass index; VD: vitamin D; Ca:calcium; Mg: magnesium.

Supplementary Figure 4

Comparison-adjusted funnel plot

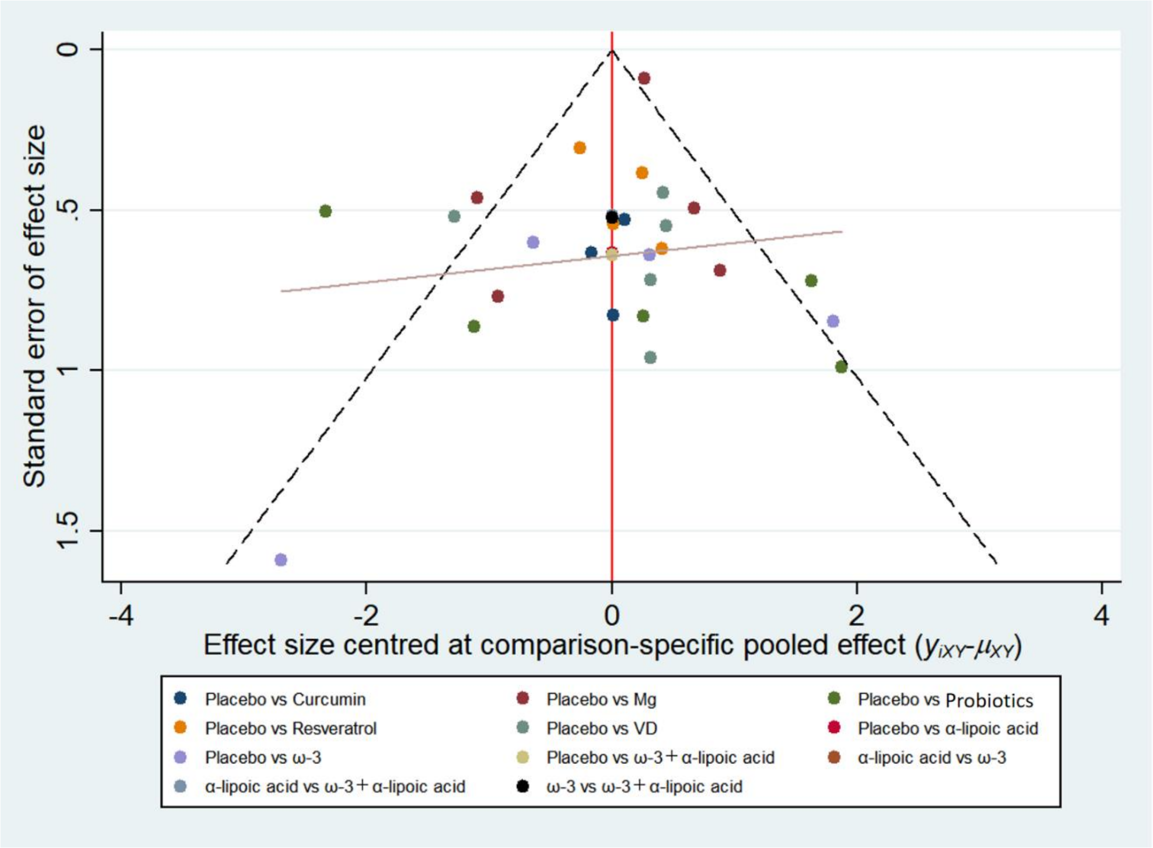

Note: VD: vitamin D; Ca:calcium; Mg: magnesium.
